# Long non-coding RNA LINC00665 promotes gemcitabine resistance of Cholangiocarcinoma cells via regulating EMT and stemness properties through miR-424-5p/BCL9L axis

**DOI:** 10.1038/s41419-020-03346-4

**Published:** 2021-01-12

**Authors:** Min Lu, Xinglei Qin, Yajun Zhou, Gang Li, Zhaoyang Liu, Xiwen Geng, Haodi Yue

**Affiliations:** 1 Department of Cardiology, Henan Provincial People’s Hospital, People’s Hospital of Zhengzhou University, People’s Hospital of Henan University, School of Clinical Medicine of Henan University, Zhengzhou, 450003 China; 2 Department of Hepatobiliary Surgery, Henan Provincial People’s Hospital, People’s Hospital of Zhengzhou University, People’s Hospital of Henan University, School of Clinical Medicine of Henan University, Zhengzhou, 450003 China; 3 Center for Clinical Single Cell Biomedicine, Henan Provincial People’s Hospital, People’s Hospital of Zhengzhou University, People’s Hospital of Henan University, School of Clinical Medicine of Henan University, Zhengzhou, 450003 China

**Keywords:** Cell signalling, Cancer therapeutic resistance

## Abstract

Gemcitabine is the first-line chemotherapy drug for cholangiocarcinoma (CCA), but acquired resistance has been frequently observed in CCA patients. To search for potential long noncoding RNAs (lncRNAs) involved in gemcitabine resistance, two gemcitabine resistant CCA cell lines were established and dysregulated lncRNAs were identified by lncRNA microarray. Long intergenic non-protein coding RNA 665 (LINC00665) were found to rank the top 10 upregulated lncRNAs in our study, and high LINC00665 expression was closely associated with poor prognosis and chemoresistance of CCA patients. Silencing LINC00665 in gemcitabine resistant CCA cells impaired gemcitabine tolerance, while enforced LINC00665 expression increased gemcitabine resistance of sensitive CCA cells. The gemcitabine resistant CCA cells showed increased EMT and stemness properties, and silencing LINC00665 suppressed sphere formation, migration, invasion and expression of EMT and stemness markers. In addition, Wnt/β-Catenin signaling was activated in gemcitabine resistant CCA cells, but LINC00665 knockdown suppressed Wnt/β-Catenin activation. B-cell CLL/lymphoma 9-like (BCL9L), the nucleus transcriptional regulators of Wnt/β-Catenin signaling, plays a key role in the nucleus translocation of β-Catenin and promotes β-Catenin-dependent transcription. In our study, we found that LINC00665 regulated BCL9L expression by acting as a molecular sponge for miR-424-5p. Moreover, silencing BCL9L or miR-424-5p overexpression suppressed gemcitabine resistance, EMT, stemness and Wnt/β-Catenin activation in resistant CCA cells. In conclusion, our results disclosed the important role of LINC00665 in gemcitabine resistance of CCA cells, and provided a new biomarker or therapeutic target for CCA treament.

## Introduction

Cholangiocarcinoma (CCA) is a group of epithelial tumors arising from different locations within the biliary tree with features of cholangiocyte differentiation. It is the second most common primary hepatic malignancy, accounting for 15–20% new diagnosed cases^1^. According to the anatomical location, cholangiocarcinoma can be classified into intrahepatic (iCCA), perihilar (pCCA), and distal (dCCA) subtypes. Among them, pCCA (50%) and dCCA (40%) represent the majority of cholangiocarcinoma cases, while iCCA is less than 10% of total^2^. Surgical treatment is the preferred cure method for this disease, however only a small subset (~35%) of patients with early-stage CCA are amendable to surgical resection, and the 5-year survival rates for iCCA, pCAA, and dCCA patients are only 63%, 30%, and 23%, respectively^2^. For patients with advanced or unresectable cholangiocarcinoma, the 5-year survival rate is less than 10%^3^.

The current standard-of-care chemotherapy regimen of gemcitabine and cisplatin is often used, but the efficiency is limited due to poor response and chemoresistance, with a median overall survival of less than one year^4^. Gemcitabine (2′,2′-difluoro-2′-deoxycytidine, dFdC), a deoxycytidine analog, can be incorporated into the DNA double-strand during DNA synthesis, thus stops the chain elongation and blocks cell cycle progression^5^. As the first-line chemotherapy drug, intrinsic or acquired resistance of gemcitabine has been frequently observed in CCA patients. Besides, there is evidence that many pathways such as Wnt/β-Catenin, NF-кB, AKT, and MAPK are enrolled in gemcitabine resistance^5^. Moreover, gemcitabine resistance of cholangiocarcinoma is also proved to associate with the acquisition of epithelial-mesenchymal transition (EMT) phenotype and the existence of cancer stem-like cells (CSC) in tumor mass^6,7^. These cholangiocarcinoma cells are highly prone to metastasis and refractory to gemcitabine treatment.

Long non-coding RNAs (LncRNAs) are a widespread class of RNA transcripts longer than 200 nucleotides that are not translated into proteins. Despite they have no protein-coding potential, lncRNAs are typically transcribed and spliced like other protein-coding genes, though in a more tissue-specific manner^8^. Initially, lncRNAs are simply regarded as the consequence of transcriptional noise, but recent studies indicate that lncRNAs are enrolled in many cellular processes such as regulation of gene expression, chromatin structure regulation, and epigenetic modification^9^. LncRNAs also play an important role in the initiation, progression, and chemoresistance of cancer, including cholangiocarcinoma. For example, Low BAP1 expression is proved to be closely associated with increased gemcitabine sensitivity in cholangiocarcinoma, and exogenous modulation of a BAP1 dependent lncRNA NEAT-1 evidently influence gemcitabine sensitivity and tumor cell phenotype^10^. Long intergenic non-protein coding RNA 665 (LINC00665) is a lncRNA located in chromosome 19, and recent studies indicate that LINC00665 is dysregulated and enrolled in the tumorigenesis and chemoresistance in a variety of cancers^11,12^. Moreover, transcriptome sequencing studies of cholangiocarcinoma patients reveal that LINC00665 is upregulated in cholangiocarcinoma tissues, but the exact role of this lncRNA is uncertain^13,14^.

In the present study, we established two gemcitabine-resistant CCA cell lines, and used microarray to search for lncRNAs involved in gemcitabine resistance. We found that LINC00665 was upregulated in gemcitabine-resistant CCA cell lines and associated with poor prognosis and chemoresistance of CCA patients. Silencing LINC00665 impaired gemcitabine tolerance of resistant CCA cells, while LINC00665 overexpression increased gemcitabine resistance in sensitive CCA cells. Silencing LINC00665 also suppressed gemcitabine-induced EMT, stemness, and Wnt/β-Catenin activation. Moreover, LINC00665 regulated BCL9L expression by acting as a molecular sponge for miR-424-5p, while enforced miR-424-5p expression or silencing BCL9L abolished the effects mediated by LINC00665 in resistant CCA cells. Our results provided a feasible strategy for searching gemcitabine-resistant related lncRNAs and identified a new target for overcoming gemcitabine resistance in CCA patients.

## Results

### LINC00665 is upregulated in gemcitabine-resistant CCA cell lines and associated with poor prognosis and chemoresistance of CCA patients

**Fig. 1:**
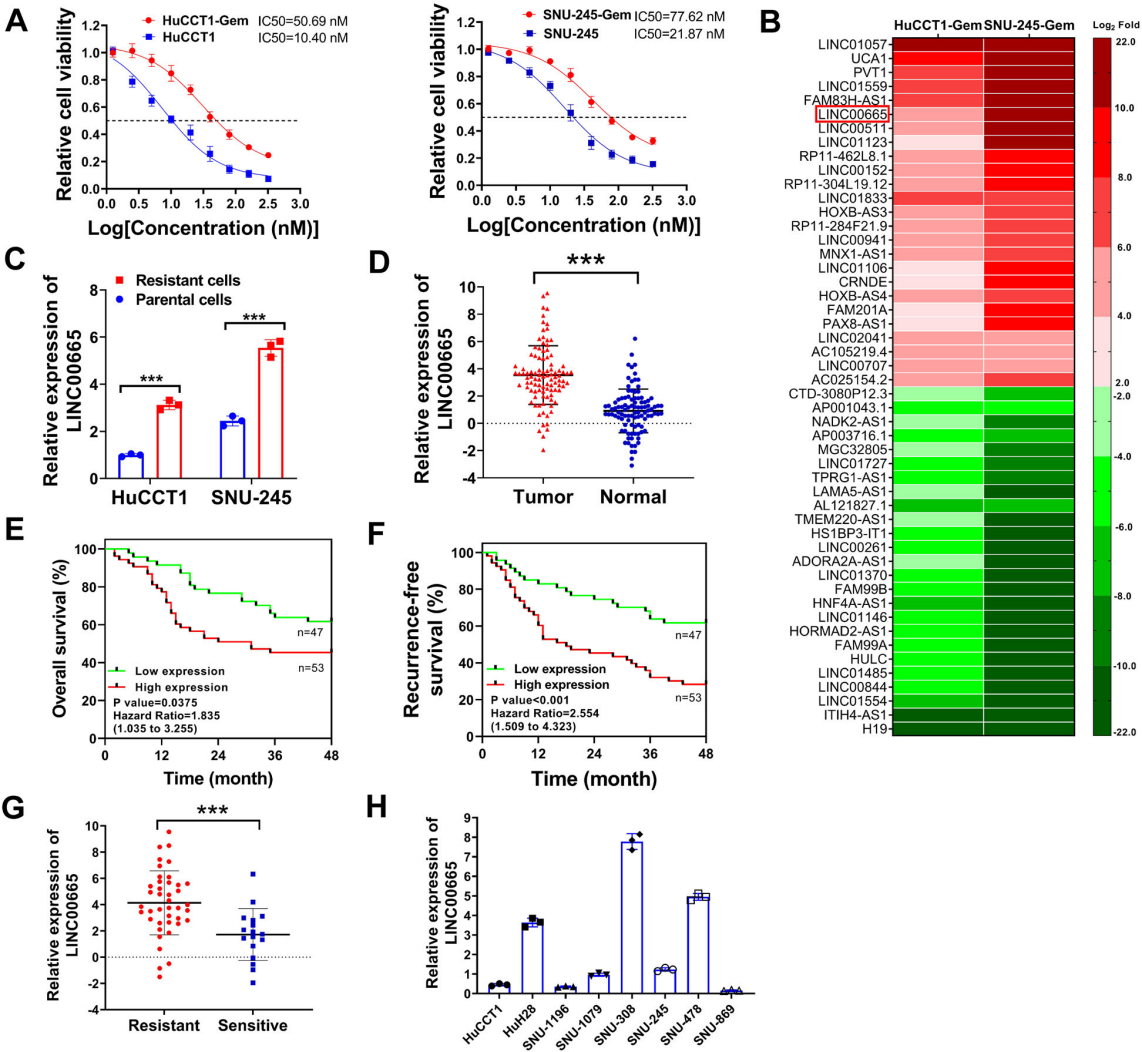
LINC00665 is upregulated and associated with poor prognosis and chemoresistance in CCA patients. **A** HuCCT1, HuCCT1-Gem, SNU-245, and SNU-245-Gem cells were seeded in 96-well plates (5000 cells/well) and exposed to 0, 1.25, 2.50, 5.0, 6.25, 10, 20, 40, 80, 160, and 320 nM gemcitabine for 6 d, then assayed for cell viability. **B** heat map shows the top 50 dysregulated lncRNAs in HuCCT1-Gem and SNU-245-Gem cells identified by lncRNA microarray. **C** relative expression of LINC00665 in parental or resistant HuCCT1 and SNU-245 cells was evaluated by qRT-PCR. **D** relative expression of LINC00665 in 100 cholangiocarcinoma samples (Tumor) and paired adjacent non-cancerous tissues (Normal) was evaluated by qRT-PCR. **E**, **F** Kaplan–Meier survival analysis of CCA patients according to LINC00665 expression. Overall survival (**E**) and recurrence-free survival (**F**) were shown. **G** relative expression of LINC00665 in chemotherapy-resistant or sensitive CCA patients was shown. **H** relative expression of LINC00665 in CCA cell lines. ^***^P ≤ 0.05.

The chemotherapy regimen of gemcitabine and cisplatin is often used in CCA patients with advanced or unresectable disease, but the efficiency is often limited due to the acquired chemoresistance of CCA cells. To disclose the possible role of lncRNAs in chemoresistance of CCA cells, two gemcitabine-resistant CCA cell lines (HuCCT1-Gem and SNU-245-Gem) were established by exposing the parental HuCCT1 and SNU-245 cells to increasing concentrations of gemcitabine for 8 months. Gemcitabine-resistant CCA cell lines (HuCCT1-Gem and SNU-245-Gem) showed an obvious increased tolerance of gemcitabine compared with parental cell lines, with increased half-maximal inhibitory concentration (IC50) of gemcitabine from 10.40 nM to 50.69 nM (HuCCT1 *vs*. HuCCT1-Gem) and 21.87 nM to 77.62 nM (SNU-245 *vs*. SNU-245-Gem) (Fig. 1A). When cultured in soft agar and treated with a series of concentrations of gemcitabine, HuCCT1-Gem and SNU-245-Gem formed more colonies in the highest gemcitabine concentration group, while HuCCT1 and SNU-245 showed nearly no colonies (Figure S1A and S1B). These results further confirmed the increasing gemcitabine tolerance of HuCCT1-Gem and SNU-245-Gem cells. To screen for lncRNAs that are involved in gemcitabine resistance of CCA cells, lncRNA microarrays were conducted to evaluate the dysregulated lncRNAs (|Log2 fold change | ≥2) in the two pairs of CCA cell lines (HuCCT1 *vs*. HuCCT1-Gem, and SNU-245 *vs*. SNU-245-Gem). As shown in Supplementary Table 3, 744 lncRNAs were dysregulated in HuCCT1-Gem cells compared with HuCCT1, and 538 lncRNAs were dysregulated in SNU-245-Gem cells compared with SNU-245. A total of 108 aberrant expressed lncRNAs were found both in HuCCT1-Gem and SNU-245-Gem cells, indicating their potential role in gemcitabine resistance of CCA cells (Supplementary Table 4). The top 50 dysregulated lncRNAs were shown in Fig. 1B. Among them, lncRNAs PVT1, H19, and UCA1 were proved to involve in gemcitabine resistance in other cancers and CCA progression^15–20^, suggested that our screen strategy for gemcitabine resistance-related lncRNAs were feasible.

The top 10 upregulated or downregulated lncRNAs were further examined in our study, and we finally come to long intergenic non-protein coding RNA 665 (LINC00665). LINC00665 ranked the top 10 upregulated lncRNAs in our screen (Fig. 1B), and several other studies suggested the tumorigenesis and chemoresistance function of this lncRNA in a variety of cancers^11,12,21,22^. The expression of LINC00665 in these two pair cell lines was validated by qRT-PCR, and our results demonstrated the apparently upregulation of LINC00665 in gemcitabine resistant CCA cells (Fig. 1C). Previous transcriptome-sequencing studies also indicated that LINC00665 was overexpressed in tumor tissues of CCA patients^13,14,23^, thus we further checked the expression of LINC00665 in 100 pairs of CCA samples and matched adjacent-normal tissues collected from Henan Provincial People’s Hospital. Our results revealed that LINC00665 was significantly upregulated in CCA patients, corresponding with previous results (Fig. 1D). In addition, we designed the in situ hybridization probes for LINC00665 and evaluated its expression by in situ hybridization analysis in CCA samples. We found that LINC00665 were significantly increased in CCA tissues compared with normal matched adjacent-normal tissues, and mainly located in the cytoplasm (Figure S1C). The correlation of LINC00665 expression with clinicopathological characteristics of CCA patients was further analyzed, and CCA patients were divided into LINC00665 high expression group and low expression group by using the median of LINC00665 expression as a cut-off value. We found that high expression of LINC00665 was positively associated with a higher TNM stage, lymph node metastasis, and distant metastasis in CCA patients (Table 1). In Kaplan-Meier survival analysis, CCA patients with high LINC00665 expression had significantly shorter overall survival time and recurrence-free survival time (Fig. 1E and F). Notably, more than half of the CCA patients enrolled in our study had received conventional chemotherapy before surgery, and typically these chemotherapy regimens were based on gemcitabine (with cisplatin derivatives, capecitabine, or S-1). Patients who had stable disease or tumor regression (partial response) after receiving gemcitabine-based chemotherapy were considered as chemotherapy-sensitive, while the rest of these patients were regarded as chemotherapy-resistant. We divided those patients into chemotherapy-resistant or sensitive groups according to their response to the chemotherapy drugs, and we found that high expression of LINC00665 was more commonly appeared in the resistant group, while most sensitive patients showed lower levels of LINC00665 (Fig. 1G). We also evaluated the expression of LINC00665 in several frequently used CCA cell lines, and found that LINC00665 was highly expressed in HuH28, SNU-308, and SNU-478 cells (Fig. 1H). Though parental HuCCT1 and SNU-245 cells had lower expression levels of LINC00665, expression of LINC00665 in the gemcitabine resistant counterparts of them was significantly increased (Fig. 1C).

**Table 1:** Correlation between LINC00665 expression and clinicopathological characteristics in cholangiocarcinoma patients.

Characteristics	*n*	LINC00665 expression		*P* value
		Low	High	
Age (Years)				0.911
≤60	41	19	22	
>60	59	28	31	
Sex				0.975
Male	49	23	26	
Female	51	24	27	
Tumor size (mm)				0.093
≥ 30	66	23	43	
<30	34	24	10	
TNM stage				**0.022**
I–II	39	27	12	
III–IV	61	20	41	
Histological grade				0.268
Well	69	37	32	
Moderate-Poor	31	10	21	
Lymph node metastasis				**0.005**
Positive	58	18	40	
Negative	42	19	23	
Distant metastasis				**0.020**
Positive	45	14	31	
Negative	55	33	22	
Tumor location				0.810
Intrahepatic	23	13	10	
Perihilar	37	14	23	
Distal	50	20	30	

### LINC00665 knockdown impairs gemcitabine tolerance in resistant CCA cells

**Fig. 2:**
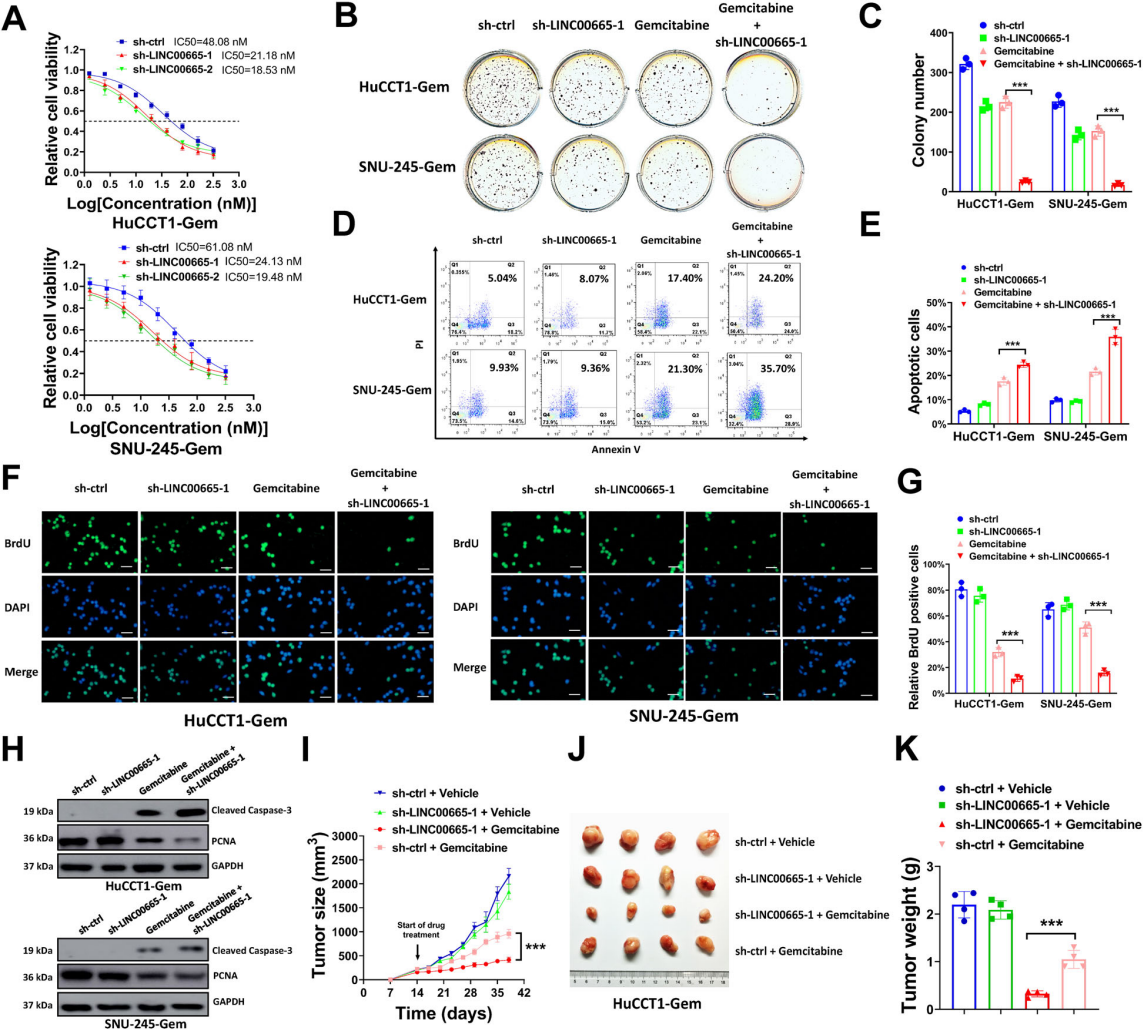
LINC00665 knockdown impairs gemcitabine tolerance in resistant CCA cells. **A** HuCCT1-Gem and SNU-245-Gem cells transduced with sh-LINC00665-1, sh-LINC00665-2, or sh-ctrl were seeded in 96-well plates (5000 cells/well) and exposed to 0, 1.25, 2.50, 5.0, 6.25, 10, 20, 40, 80, 160, and 320 nM gemcitabine for 6 d, then assayed for cell viability. **B**, **C** HuCCT1-Gem, and SNU-245-Gem cells transduced with sh-LINC00665-1 or sh-ctrl were seeded in soft agar (8000 cells/well), then treated with 10 nM gemcitabine or equal volume of PBS. Represent image of plates (**B**) and number of colonies (**C**) were shown. **D**–**H**, HuCCT1-Gem and SNU-245-Gem cells transduced with sh-LINC00665-1 or sh-ctrl were treated with 10 nM gemcitabine or equal volume of PBS, then used for flow cytometry analysis (**D**, **E**), Brdu assay (**F**, **G**), and western blot (**H**). **I**–**K** HuCCT1-Gem cells (2 × 10^6^) transduced with sh-LINC00665-1 or sh-ctrl were subcutaneous injected into nude mice, then treated with gemcitabine (100 mg/kg) or an equal volume of PBS (Vehicle control) for three weeks. Tumor growth curves (**I**), represent images (**J**) and tumor weight (**K**) were shown. Scale bars = 100 μm for BrdU assay. ^***^P ≤ 0.05.

To disclose the potential role of LINC00665 in gemcitabine resistant CCA cells, we knocked down LINC00665 expression using two small hairpin RNA (shRNA) specifically targeting LINC00665 (sh-LINC0065-1 and sh-LINC00665-2). As shown in Figure S2, the expression of LINC00665 was dramatically downregulated in HuCCT1-Gem and SNU-245-Gem cells when transduced with sh-LINC0065-1 or sh-LINC00665-2, indicating these two shRNAs were available for LINC00665 knockdown. Gemcitabine tolerance was evaluated by measuring gemcitabine IC50 of these cells. As we expected, silencing LINC00665 significantly decreased the gemcitabine IC50 in HuCCT1-Gem and SNU-245-Gem cells, suggesting that LINC00665 knockdown impaired gemcitabine tolerance (Fig. 2A). This was further evaluated in soft agar assay, and we found that silencing LINC00665 or treated with low dose gemcitabine (10 nM) slightly suppressed colony formation of HuCCT1-Gem and SNU-245-Gem cells, but the combination of them had strengthened repression on colony formation, indicating the LINC00665 knockdown had additional effects on gemcitabine cytotoxicity (Fig. 2B and C). Gemcitabine is a deoxycytidine analog, whose cytotoxic activity is based on hindering DNA synthesis, and subsequently suppressing cell growth and inducing apoptosis. To further evaluate the influence of LINC00665 knockdown on gemcitabine resistance, we conducted flow cytometry, BrdU assay, western blot, and tumor xenograft formation assay. Our results revealed that silencing LINC00665 alone had little impact on cell apoptosis and growth of HuCCT1-Gem and SNU-245-Gem cells, with no obvious increase in PI and Annexin-V double-positive subsets (Fig. 2D and E) and BrdU positive cells (Fig. 2F and G). However, LINC00665 knockdown significantly increased the cytotoxicity of gemcitabine, with apparently enlarged PI and Annexin-V double-positive subsets (Fig. 2D and E) and BrdU positive cells (Fig. 2F and G) compared with gemcitabine treatment alone. Cleaved caspase-3 is the marker of cell apoptosis, and proliferating cell nuclear antigen (PCNA) is the marker of cell growth. The protein expression of cleaved Caspase-3 and PCNA was evaluated by western blot, and we found that gemcitabine treatment increased cleaved Caspase-3 expression and decreased PCNA level, and this effect was further strengthened by silencing LINC00665 (Fig. 2H). In tumor formation assay, gemcitabine group showed a moderate suppression on tumor growth of HuCCT1-Gem cells in nude mice, but this was significantly enforced by silencing LINC00665 (Fig. 2I–K). Taken together, our results suggested that LINC00665 knockdown increased the cytotoxic activity of gemcitabine on cell apoptosis and growth, thus impaired gemcitabine tolerance of resistant CCA cells.

### LINC00665 overexpression increases gemcitabine tolerance in sensitive CCA cells in vitro and in vivo

**Fig. 3:**
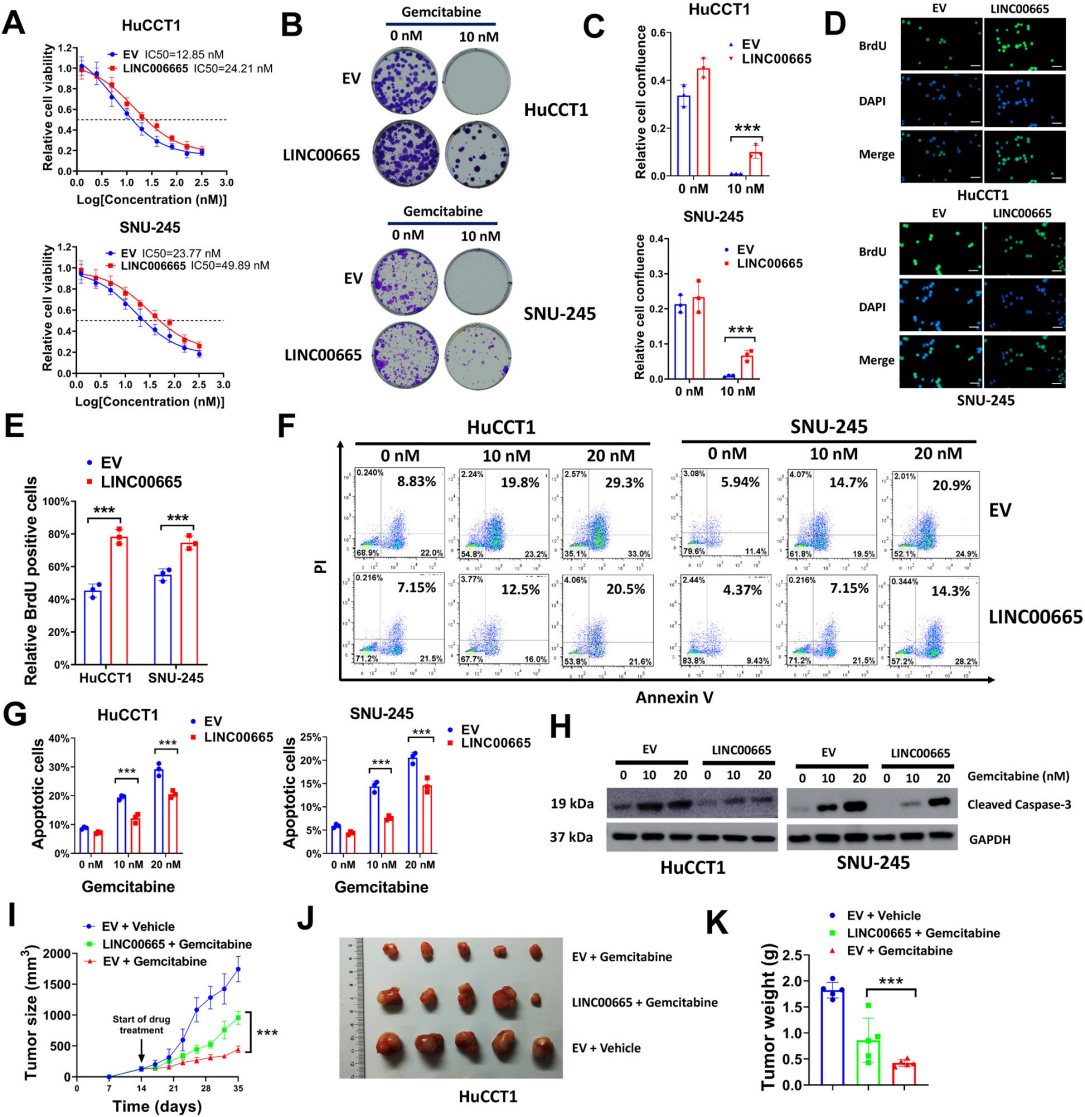
Enforced LINC00665 expression increases gemcitabine tolerance in sensitive CCA cells in vitro and in vivo. **A** HuCCT1 and SNU-245 cells transduced with LINC00665 expression lentivirus or EV were seeded in 96-well plates (5000 cells/well) and exposed to 0, 1.25, 2.50, 5.0, 6.25, 10, 20, 40, 80, 160, and 320 nM gemcitabine for 6 d, then assayed for cell viability. **B**, **C** HuCCT1 and SNU-245 cells (3000/well) transduced with LINC00665 expression lentivirus or EV were seeded in 6-well plates for colony formation assay, meanwhile treated with 0 or 10 nM gemcitabine. Represent plates (**B**) and relative cell confluence (**C**) were shown. **D**, **E** HuCCT1 and SNU-245 cells transduced with LINC00665 expression lentivirus or EV were treated with gemcitabine (10 nM) for 24 h, then cells were used for BrdU assay. Represent images (**D**) and relative BrdU positive cells were shown (**E**). **F**–**H** HuCCT1 and SNU-245 cells transduced with LINC00665 expression lentivirus or EV were treated with gemcitabine (0 nM, 10 nM, or 20 nM) for 3 days, then cells were stained with Annexin V-FITC and PI for flow cytometry (**F**, **G**), or collected cell lysates for western blot (**H**). **I**–**K** HuCCT1 cells (2 × 10^6^) transduced with LINC00665 expression lentivirus or EV were subcutaneous injected into nude mice, then treated with gemcitabine (100 mg/kg) or equal volume of PBS (Vehicle control) for three weeks. Tumor growth curves (**I**), represent images (**J**), and tumor weight (**K**) were shown. Scale bars = 100 μm for BrdU assay. ^***^P ≤ 0.05.

To determine whether LINC00665 upregulation could increase gemcitabine resistance of CCA cells, we overexpressed LINC00665 in parental HuCCT1 and SNU-245 cells by transducing them with LINC00665 expression lentivirus vector. HuCCT1 and SNU-245 cells transduced with LINC00665 expression lentivirus vector had significantly increased LINC00665 levels compared with empty vector (EV) control (Figure S3). Next, we found that LINC00665 overexpression increased the gemcitabine IC50 of HuCCT1 and SNU-245 cells by nearly two-fold, indicating that LINC00665 overexpression could increase gemcitabine tolerance in sensitive CCA cells (Fig. 3A). This was further confirmed by colony formation assay, as our results demonstrated that LINC0065 overexpression promoted colony formation of HuCCT1 and SNU-245 cells under gemcitabine treatment condition (Fig. 3B and C). In BrdU assay, HuCCT1 and SNU-245 cells were transduced with EV or LINC00665 expression lentivirus, then treated with 10 nM gemcitabine. We found that the percentage of BrdU positive cells was increased in LINC00665 overexpressed cells (Fig. 3D and E). Moreover, LINC00665 overexpression also suppressed gemcitabine-induced apoptosis of HuCCT1 and SNU-245 cells, as indicated in flow cytometry analysis (Fig. 3F and G). This was further verified by western blot, as the expression of cleaved Caspase-3 was decreased in LINC00665 overexpression cells (Fig. 3H). The influence of LINC00665 overexpression on gemcitabine resistance was also evaluated by tumor formation assay in vivo. The tumor growth of HuCCT1 cells was significantly suppressed by gemcitabine treatment, but LINC00665 overexpression partially reversed this effect (Fig. 3I–K). Collectively, our results demonstrated that LINC00665 overexpression increased gemcitabine tolerance in sensitive CCA cells in vitro and in vivo.

### Silencing LINC00665 suppresses gemcitabine-induced EMT and stemness properties in resistant CCA cells

**Fig. 4:**
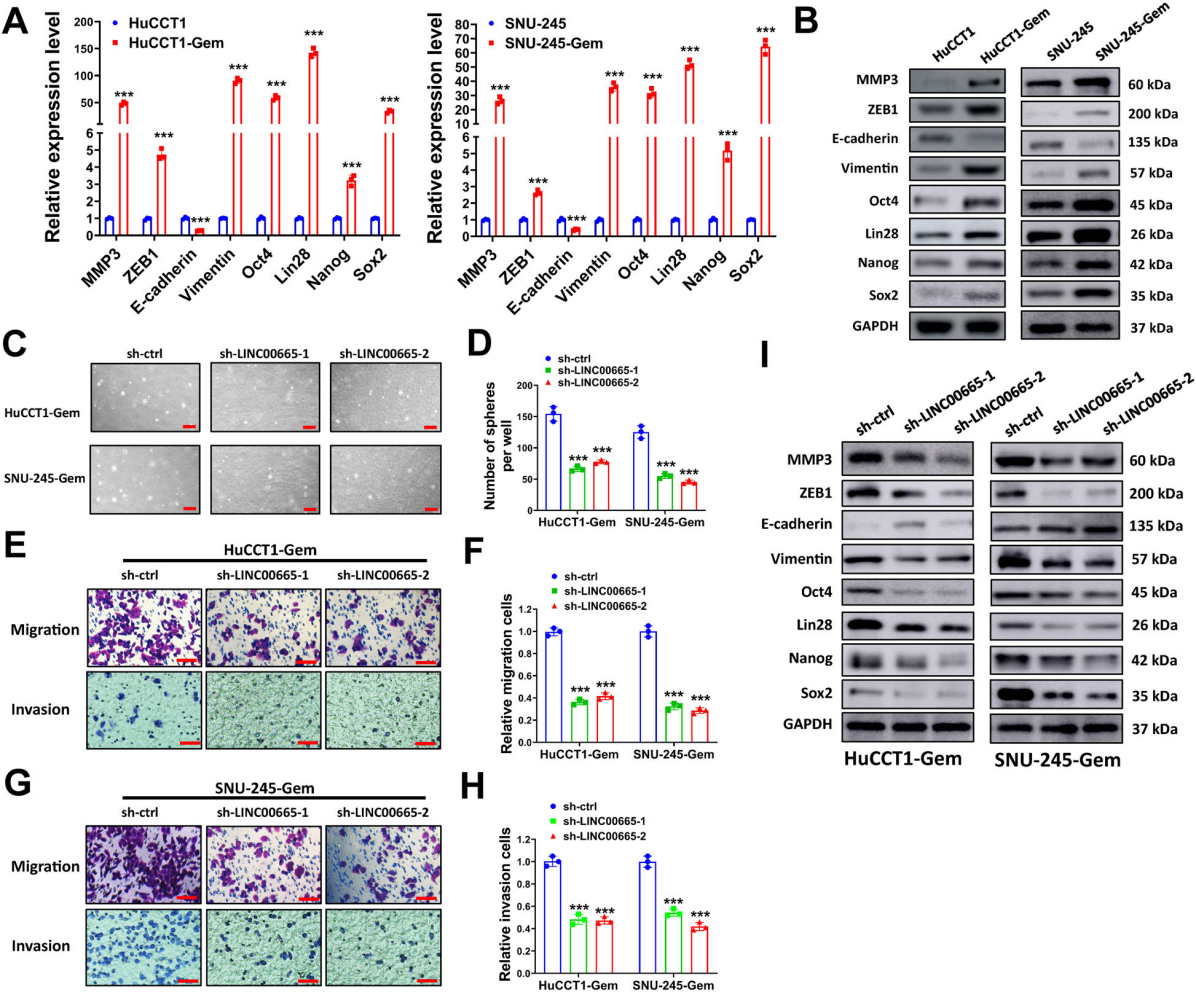
Silencing LINC00665 suppresses EMT and stemness properties in gemcitabine-resistant CCA cells. **A**, **B** relative expression of EMT markers (MMP3, ZEB1, E-cadherin, and Vimentin) and CSC markers (Oct4, Lin28, Nanog and Sox2) in resistant or sensitive CCA cells were evaluated by qRT-PCR (**A**) or western blot (**B**). **C**–**H** HuCCT1-Gem and SNU-245-Gem cells transduced with sh-LINC00665-1, sh-LINC00665-2, or sh-ctrl were used for sphere formation assay (**C**, **D**), and transwell cell migration and invasion assays (**E**–**G**). Represent images of tertiary spheres (**C**) and migration or invasion cells (**E**, **G**), and a number of spheres (**D**) and relative migration or invasion cells (**F**, **H**) were shown. **I** HuCCT1-Gem and SNU-245-Gem cells were transduced with sh-LINC00665-1, sh-LINC00665-2, or sh-ctrl, then cell lysates were collected for western blot. Scale bars = 100 μm for sphere formation assay, and scale bars = 50 μm for transwell cell migration and invasion assay. ^***^P ≤ 0.05.

Accumulated studies suggest that acquiring of EMT phenotype and/or existence of a subpopulation of cancer stem-like cells in tumor mass are associated with gemcitabine resistance in cancers^24,25^. We speculated that long time exposure to gemcitabine could induce EMT phenotype and enrich cancer stem-like cell subsets, thus we conducted sphere formation assay, transwell cell invasion, and migration assays to evaluated EMT and stemness properties in gemcitabine resistant CCA cells. In our study, HuCCT1-Gem and SNU-245-Gem cells formed more spheres compared with parental HuCCT1 and SNU-245 cells (Figure S4A and S4B). Moreover, HuCCT1-Gem and SNU-245-Gem cells also showed increased cell migration and invasion (Figure S4C–S4E). Finally, the expression of EMT markers (MMP3, ZEB1, E-cadherin, and Vimentin) and CSC markers (Oct4, Lin28, Nanog, and Sox2) were evaluated by qRT-PCR and western blot. We found that the mRNA and protein expression of MMP3, ZEB1, Vimentin, Oct4, Lin28, Nanog, and Sox2 was upregulated in HuCCT1-Gem and SNU-245-Gem cells while E-cadherin was downregulated compared with parental HuCCT1 and SNU-245 cells (Fig. 4A and B). These results indicated that long time gemcitabine treatment-induced EMT phenotype and stemness properties of HuCCT1-Gem and SNU-245-Gem cells. To explore the potential function of LINC00665 upregulation in gemcitabine-induced EMT phenotype and stemness properties, we silenced LINC00665 in HuCCT1-Gem and SNU-245-Gem cells and evaluated the changes in sphere formation, transwell cell migration and invasion, and expression of EMT and CSC makers. Silencing LINC00665 evidently decreased the sphere formation of HuCCT1-Gem and SNU-245-Gem cells (Fig. 4C and D). In addition, transwell migration and invasion cells were dramatically reduced after LINC00665 knockdown (Fig. 4E–H). In western blot analysis, the expression of EMT and CSC makers MMP3, ZEB1, Vimentin, Oct4, Lin28, Nanog, and Sox2 was decreased in HuCCT1-Gem and SNU-245-Gem cells while E-cadherin was increased after LINC00665 knockdown. To further confirm the influence of LINC00665 on EMT and stemness properties, we also overexpressed LINC00665 in gemcitabine-sensitive CCA cells. We found that LINC00665 overexpression facilitated sphere formation, transwell migration, and invasion of HuCCT1 and SNU-245 cells (Figure S4F–J). Above all, these results indicated that silencing LINC00665 suppressed gemcitabine-induced EMT and stemness properties in resistant CCA cells.

### Silencing LINC00665 represses Wnt/β-Catenin signaling and BCL9L expression in gemcitabine-resistant CCA cells

**Fig. 5:**
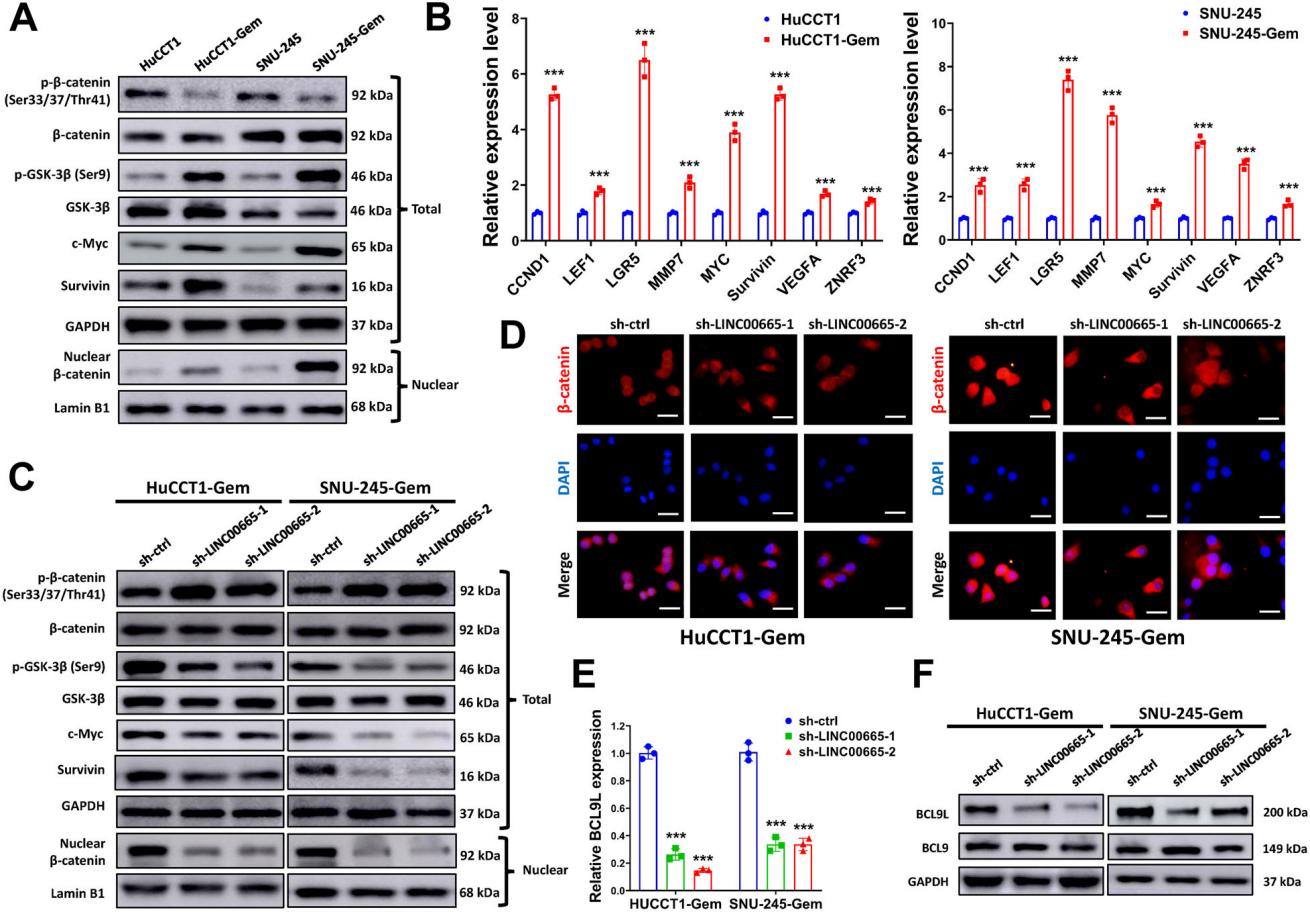
Silencing LINC00665 represses Wnt/β-Catenin signaling and BCL9L expression in gemcitabine-resistant CCA cells. **A**, **B** expression of indicated genes in Wnt/β-Catenin pathway (**A**) downstream targets (**B**) was evaluated by western blot or qRT-PCR. **C** HuCCT1-Gem and SNU-245-Gem cells were transduced with sh-LINC00665-1, sh-LINC00665-2, or sh-ctrl, then the expression of indicated genes in Wnt/β-Catenin pathway was evaluated by western blot. **D** immunofluorescent staining showed the subcellular location of β-Catenin in HuCCT1-Gem and SNU-245-Gem cells after transducing with sh-LINC00665-1, sh-LINC00665-2, or sh-ctrl. DAPI was used to stain the nucleus. Scale bars = 50 μm. **E**, **F** expression of BCL9L in HuCCT1-Gem and SNU-245-Gem cells transduced with sh-LINC00665-1, sh-LINC00665-2, or sh-ctrl was evaluated by qRT-PCR (**E**) and western blot (**F**). ^***^P ≤ 0.05.

Emerging evidence indicates that Wnt/β-Catenin signaling is involved in gemcitabine-resistance of cancer cells^26^, maintaining of cancer stem-like cell properties^27^, and regulation of EMT^28^. In our study, we found that Wnt/β-Catenin signaling was activated in HuCCT1-Gem and SNU-245-Gem cells, with increased phosphorylation of GSK-3β, decreased phosphorylation, and increased nucleus translocation of β-Catenin (Fig. 5A). Moreover, the conventional downstream genes of Wnt/β-Catenin signaling were upregulated in HuCCT1-Gem and SNU-245-Gem cells (Fig. 5B). To disclose the connection between LINC00665 and Wnt/β-Catenin signaling, we knocked down LINC00665 in HuCCT1-Gem and SNU-245-Gem cells and evaluated the variation of Wnt/β-Catenin pathway. Indeed, LINC00665 knockdown suppressed nucleus translocation of β-Catenin and phosphorylation of GSK-3β, and increased phosphorylation of β-Catenin (Fig. 5C). At the same time, the expression of downstream target genes in Wnt/β-Catenin signaling such as c-Myc and Survivin were reduced (Fig. 5C and Figure S5A-S5B). The subcellular location of β-Catenin in HuCCT1-Gem and SNU-245-Gem cells was evaluated by immunofluorescent staining. As shown in Fig. 5D, HuCCT1-Gem and SNU-245-Gem cells had more nucleus accumulation of β-Catenin, but this was abolished by LINC00665 knockdown, suggesting that silencing LINC00665 repressed the nucleus translocation of β-Catenin, and subsequently suppressed Wnt/β-Catenin activation.

Wnt/β-Catenin signaling is tightly regulated by a variety of genes such as cytoplasmic signaling regulators APC, AXIN1, and AXIN2, and nucleus transcriptional regulators BCL9, BCL9L, CBP, SPDEF, TCF-3, TCF-4, and TCF-7. As silencing LINC00665 suppressed the activation of Wnt/β-Catenin signaling in resistant CCA cells, we supposed that some genes involved in regulating Wnt/β-Catenin signaling pathway might be influenced by LINC00665 knockdown. Thus, the expression of a variety Wnt/β-Catenin regulators was assessed by qRT-PCR (Figure S5C and S5D). Though some genes were slightly downregulated by LINC00665 knockdown, we were surprised to find that the expression of BCL9L was dramatically decreased (Fig. 5E, Figure S5C and S5D). This was further confirmed by western blot, as LINC00665 knockdown decreased protein expression of BCL9L (Fig. 5F). BCL9L (B-cell CLL/lymphoma 9-like, B9L or BCL9-2), the mammalian homologs of the *Drosophila* gene *legless*, plays a key role in nucleus translocation of β-Catenin and promotes β-Catenin-dependent transcription^29^. Besides, BCL9L is involved in Wnt-mediated regulation of stem cell traits and EMT in cancers^30,31^. Therefore, we supposed that silencing LINC00665 might suppress Wnt/β-Catenin activation by downregulating BCL9L.

### LINC00665 regulates BCL9L expression by acting as a molecular sponge for miR-424-5p

The connection between lncRNAs and protein-coding messenger RNAs by competing endogenous RNAs (ceRNAs) network has been well established and demonstrated for many years^32^. Therefore, we speculated that LINC00665 might act as a molecular sponge and indirectly regulated BCL9L expression through ceRNA network. The potential microRNAs that interacted with LINC00665 (score≥0.9) were predicted by DIANA-LncBase v.2^33^. In addition, TargetScanHuman 7.2^34^ was used to predicted conservative microRNA targeting sites for BCL9L, as indicated in Supplementary Table 5. Nine microRNAs (miR-28-5p, miR-129-5p, miR-136-5p, miR-410-3p, miR-424-5p, miR-485-5p, miR-665, miR-708-5p, and miR-3064-5p) were predicted to interact with LINC00665 and BCL9L both (Supplementary Table 6). To verify their interaction with LINC00065, we overexpressed LINC00665 in HuCCT1 and SNU-245 cells and evaluated the expression of these microRNAs (Figure S6A and S6B). Among them, miR-129-5p and miR-424-5p were dramatically decreased by LINC00665. The interaction between BCL9L and these nine microRNAs were tested by transfecting HuCCT1 and SNU-245 cells with these microRNA mimics (Figure S6C and S6D). The expression of BCL9L was significantly downregulated by miR-136-5p, miR-410-3p, and miR-424-5p. Collectively, miR-424-5p was finally predicted to interact with LINC00665 and BCL9L both in our study.

**Fig. 6:**
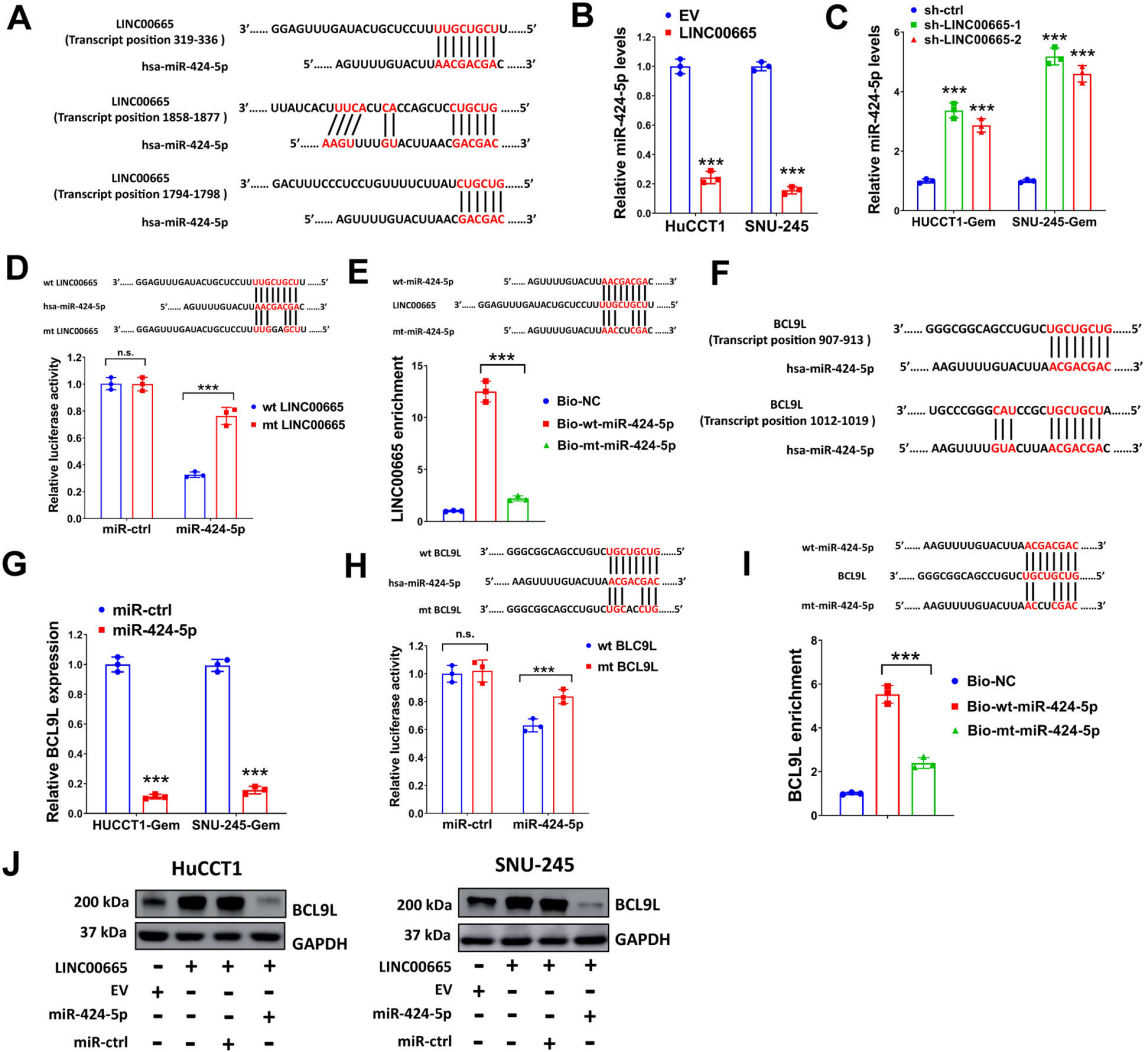
LINC00665 regulates BCL9L expression by acting as a molecular sponge for miR-424-5p. **A** the putative binding sites for LINC00665 and miR-424-5p were shown. **B** HuCCT1 and SNU-245 cells transduced with LINC00665 expression lentivirus or EV, then relative miR-424-5p expression was evaluated by qRT-PCR. **C** HuCCT1-Gem and SNU-245-Gem cells were transduced with sh-LINC00665-1, sh-LINC00665-2, or sh-ctrl, then relative miR-424-5p expression was evaluated by qRT-PCR. **D** HEK293T cells transduced with wt LINC00665 or mt LINC00665 expression pMIR-REPORT vector were used for luciferase reporter assay. Cells were co-transfected with miR-424-5p or miR-ctrl expression vector as indicated. **E** Relative expression of LINC00665 in the bound RNAs pulled down by biotinylated wild-type (wt) miR-424-5p, mutant (mt) miR-424-5p, and non-targeting control (NC) were evaluated by qRT-PCR. **F** the putative binding sites for BCL9L and miR-424-5p were shown. **G** HuCCT1-Gem and SNU-245-Gem cells transduced with miR-424-5p expression lentivirus or miR-ctrl, then relative BCL9L expression was evaluated by qRT-PCR. **H** HEK293T cells transduced with wt BCL9L or mt BCL9L expression pMIR-REPORT vector were used for luciferase reporter assay. Cells were co-transfected with miR-424-5p or miR-ctrl expression vector as indicated. **I** Relative expression of BCL9L in the bound RNAs pulled down by biotinylated wild-type (wt) miR-424-5p, mutant (mt) miR-424-5p, and non-targeting control (NC) were evaluated by qRT-PCR. **J** HuCCT1 and SNU-245 cells were transduced with LINC00665 expression lentivirus, EV, miR-424-5p, or miR-ctrl as indicated, then expression of BCL9L was evaluated by western blot. ^***^P ≤ 0.05.

The putative binding sites of LINC00665 and miR-424-5p predicted by DIANA-LncBase v.2 were shown Fig. 6A. As indicated above, enforced LINC00665 expression reduced miR-424-5p levels (Fig. 6B). On the contrary, silencing LINC00665 in HuCCT1-Gem and SNU-245-Gem cells enhanced miR-424-5p expression (Fig. 6C). The lentivirus expression vector of miR-424-5p and BCL9L were constructed and their expression were verified in resistant CCA cells (Figure S6E and S6F). In our study, the interaction between LINC00665 and miR-424-5p was further verified by luciferase reporter assay and pull-down assay. As shown in Fig. 6D, miR-424-5p overexpression reduced luciferase activity of wt LINC00665, while a 2-bp mutation in the predicted binding sites of LINC00665 (mt LINC00665) partially reversed this effect. In pull-down assay, LINC00665 was successfully pulled down by biotin-labeled wt-miR-424-5p, but miR-424-5p with 2-bp mutation in the binding sites (mt-miR-424-5p) failed (Fig. 6E). These results demonstrated that LINC00665 might have direct interaction with miR-424-5p. The putative binding sites for BCL9L and miR-424-5p predicted by TargetScanHuman 7.2 were shown in Fig. 6F. As indicated above, miR-424-5p suppressed BCL9L expression in HuCCT1-Gem and SNU-245-Gem cells (Fig. 6G). The interaction between BCL9L and miR-424-5p were verified by luciferase reporter assay and pull-down assay. As the same with LINC00665, miR-424-5p overexpression reduced luciferase activity of wt BCL9L but failed in mt BCL9L (Fig. 6H). In addition, BCL9L was successfully pulled down by biotin-labeled wt-miR-424-5p but not mt- miR-424-5p (Fig. 6I). The interaction between LINC00665, miR-424-5p, and BCL9L was further validated by western blot. We found that LINC00665 overexpression increased BCL9L protein levels, but this was abolished by restoring miR-424-5p expression in HuCCT1 and SNU-245 cells (Fig. 6J). Taken together, our results suggested that LINC00665 might regulate BCL9L expression by acting as a molecular sponge for miR-424-5p.

### Enforced miR-424-5p expression or silencing BCL9L suppresses gemcitabine resistance, EMT, stemness, and Wnt/β-Catenin signaling activation in resistant CCA cells

**Fig. 7:**
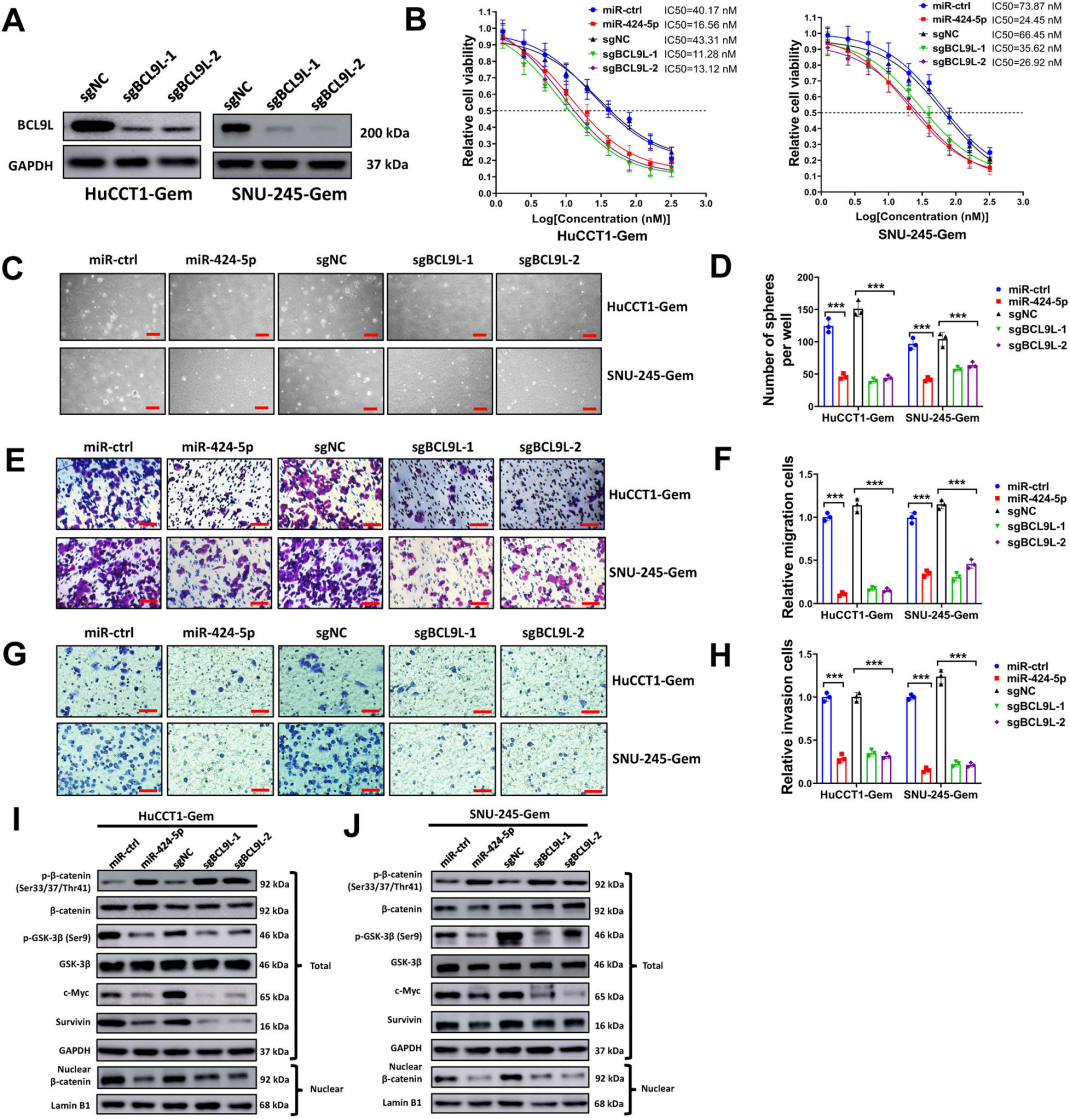
Enforced miR-424-5p expression or silencing BCL9L suppresses gemcitabine resistance, EMT, stemness, and Wnt/β-Catenin signaling activation in resistant CCA cells. **A** HuCCT1-Gem and SNU-245-Gem cells were transduced with sgBCL9L-1, sgBCL9L-2, or sgNC, then BCL9L expression was evaluated by western blot. **B** HuCCT1-Gem and SNU-245-Gem cells were transduced sgBCL9L-1, sgBCL9L-2, sgNC, miR-424-5p or miR-ctrl, then cells were seeded in 96-well plates (5000 cells/well) and exposed to 0, 1.25, 2.50, 5.0, 6.25, 10, 20, 40, 80, 160, and 320 nM gemcitabine for 6 d, and assayed for cell viability. **C**–**J** HuCCT1-Gem and SNU-245-Gem cells were transduced sgBCL9L-1, sgBCL9L-2, sgNC, miR-424-5p or miR-ctrl, the cells were used for sphere formation assay (**C**, **D**), transwell cell migration (**E**, **F**) and invasion (**G**, **H**) assay, and western blot analysis of indicated genes in Wnt/β-Catenin pathway (**I**, **J**). Scale bars = 100 μm for sphere formation assay, and scale bars = 50 μm for transwell cell migration and invasion assay. ^***^P ≤ 0.05.

Though we confirmed the interaction between LINC00665, miR-424-5p, and BCL9L in CCA cells, whether miR-424-5p/BCL9L axis was critical for the effects mediated by LINC00665 was unknown. Thus, we overexpressed miR-424-5p or knocked down BCL9L in gemcitabine-resistant CCA cells, then evaluated for gemcitabine tolerance, cell apoptosis, sphere formation, transwell cell migration and invasion, and Wnt/β-Catenin activation. In order to knock down BCL9L, we used CRISPR/Cas9 system and designed two short guide RNAs (sgRNA) specifically targeting BCL9L (sgBCL9L-1 and sgBCL9L-2). These two sgRNAs successfully downregulated the expression of BCL9L in HuCCT1-Gem and SNU-245-Gem cells (Fig. 7A). In our study, we found that miR-424-5p overexpression or silencing BCL9L increased gemcitabine sensitivity of resistant CCA cells, with decreased gemcitabine IC50 (Fig. 7B). Moreover, miR-424-5p overexpression or silencing BCL9L evidently increased apoptosis of HuCCT1-Gem and SNU-245-Gem cells under gemcitabine treatment (Figure S7A and S7B). Furthermore, miR-424-5p overexpression or silencing BCL9L reduced sphere formation (Fig. 7C and D), and repressed transwell migration and invasion (Fig. 7E–H) of HuCCT1-Gem and SNU-245-Gem cells. In tumor xenograft formation assay, we found that miR-424-5p overexpression or silencing BCL9L alone slightly suppressed tumor formation of HuCCT1-Gem cells, but dramatically increased gemcitabine sensitivity and reduced tumor growth when treated with gemcitabine (Figure S7C–E). The activation of Wnt/β-Catenin signaling was evaluated by western blot, and we found that miR-424-5p overexpression or silencing BCL9L suppressed nucleus translocation of β-Catenin and phosphorylation of GSK-3β, and increased phosphorylation of β-Catenin (Fig. 7I and J). Besides, the well-recognized downstream target genes c-Myc and Survivin were downregulated by miR-424-5p overexpression or silencing BCL9L. Above all, these results suggested that miR-424-5p/BCL9L axis was critical for LINC00665 mediated gemcitabine tolerance, EMT, stemness, and Wnt/β-Catenin signaling activation.

## Discussion

LINC00665 is frequently dysregulated in multiple cancers, and there are more and more studies focused on the potential function of LINC00665 in cancer progression and chemoresistance in recent years. For example, LINC00665 is significantly upregulated in lung adenocarcinoma pateints and predicted poor prognosis, while enforcing LINC00665 expression promotes proliferation and metastasis of lung adenocarcinoma cells by acting as ceRNA for miR-98 and subsequently activating ERK signaling^35^. In non-small-cell lung cancer, upregulation of LINC00665 confers gefitinib resistance by increasing EGFR expression and activating downstream AKT signaling, thus antagonizes gefitinib induced cell apoptosis and proliferation arrest^11^. In breast cancer, LINC00665 overexpression facilitates proliferation, migration and invasion of breast cancer cells by upregulating LIN28B and inducing EMT through ceRNA regulating of miR-379-5p^21^. Upregulation of LINC00665 are also demonstrated in prostate and gastric patients, and LINC00665 overexpression accelerates tumorigenesis and progression^12,22^. In our study, we found that LINC00665 was upregulated and predicted poor prognosis in CCA patients, and silencing LINC00665 impaired gemcitabine tolerance, repressed EMT and stemness, and inactived Wnt/β-Catenin signaling through miR-424-5p/BCL9L axis. Though the majority of studies and our results indicate the upregulation and oncogenic function of LINC00665 in cancers, some other studies provide contrary consequences. In glioma, LINC00665 is downregulated in patient samples and cell lines, and overexpression of LINC00665 is associated with inhibiting malignant behaviors of glioma cells^36^. Besides, by analyzing RNA-seq and microRNA expression profiles of serous ovarian cancer, LINC00665 is found to positively correlate with infiltration of macrophages and dendritic cells, thus possesses the potential to inhibit Treg and prevent T-cell exhaustion^37^. These contrary results reveal the complexity and heterogeneity between cancers of different origin and the diverse functions of LINC00665 in different cancers.

In our study, we found that silencing LINC00665 suppressed BCL9L expression, and subsequently decreased nucleus translocation of β-Catenin and Wnt signaling activation, therefore impaired gemcitabine-induced EMT and stemness of resistant CCA cells. To date, the function of BCL9L in cancers mainly attributes to its interaction with β-Catenin and facilitating TCF-mediated transcription, thus activates canonical Wnt signaling and its downstream EMT and stemness related genes. For example, silencing BCL9L suppresses EMT and nucleus translocation of β-Catenin in carcinoma cells, while forced expression of BCL9L induces EMT of nontransformed cells^38^. In colon cancer, conditional ablation of BCL9/BCL9L suppresses the expression of stem cell markers and generation of ulcerated colon epithelium in intestinal epithelium, and transcriptional profiles indicates that BCL9/BCL9L regulates a subset of Wnt/β-Catenin target genes controlling EMT and stemness^30^. Moreover, simultaneously BCL9/BCL9L depletion completely suppresses β-Catenin driven intestinal and hepatocellular transformation in mouse models^39^. BCL9L overexpression is positively correlated with poor overall survival in hepatocellular carcinoma patients, and silencing BCL9L, but not BCL9, reduced Wnt signaling, and suppressed cell growth and induced apoptosis of Wnt-inactive hepatocellular carcinoma cells^40^. In pancreatic cancer, BCL9L knockdown decreases cell proliferation, migration, invasion, and liver metastasis, and increases E-cadherin expression even in the presence of TGF-β, suggesting a role of BCL9L in regulating EMT^31^. These results supported our data that downregulation of BCL9L suppressed Wnt/β-Catenin mediated EMT and stemness, and eventually impaired gemcitabine tolerance of resistant CCA cells.

Gemcitabine is broadly used to treat a variety of solid tumors such as pancreatic cancer, breast cancer, ovarian cancer, non-small cell lung cancer, and cholangiocarcinoma. Despite its relatively common use, molecular mechanisms concern with gemcitabine resistance are not fully understood. In our study, two gemcitabine resistant CCA cell line was constructed by exposing to increasing concentrations of gemcitabine for nearly 8 months. These two resistant cell lines exhibited increasing EMT and stemness properties and Wnt/β-Catenin activity. Silencing LINC00665 suppressed EMT, stemness and Wnt/β-Catenin signaling, thus impaired gemcitabine tolerance of these resistant CCA cells. Our data indicates that Wnt/β-Catenin controlled EMT and stemness plays an important role in acquired gemcitabine resistance of these CCA cells. Indeed, EMT, cancer stemness and Wnt/β-Catenin signaling are demonstrated to correlate with gemcitabine resistance. For example, Wnt/β-Catenin signaling regulates ZEB1 expression in mantle cell lymphoma, and silencing ZEB1 suppresses cell proliferation and xenograft growth, and increases gemcitabine sensitivity of mantle cell lymphoma cells^41^. Besides, masitinib increases gemcitabine cytotoxicity in gemcitabine-refractory pancreatic cancer cell lines, and this was partially due to downregulating Wnt/β-Catenin signaling^42^. In genetic engineered mouse models of pancreatic cancer, restrain EMT by depletion of Snail or Twist does not affect metastasis, but enhanced gemcitabine sensitivity and increases overall survival of mice, highlights the importance of EMT in gemcitabine resistance^24^. Moreover, two new pancreatic cancer cell lines show more sphere formation and increasing expression stem markers, and their intrinsic gemcitabine resistance is attributed to their cancer stemness properties^43^.

In summary, our results demonstrated that upregulation of LINC00665 was involved in gemcitabine-resistance of CCA cells, and this was due to ceRNA regulation of miR-424-5p/BCL9L axis, thus activated Wnt/β-Catenin signaling and its downstream EMT and stemness related genes, and eventually promoted EMT and stemness properties of CCA cells. Our findings suggest that LINC00665 might be a potential biomarker or therapeutic targets for CCA treatment with gemcitabine-based chemotherapy.

## Materials and methods

### Patient samples

Written informed consent was obtained from all participants in our study. The use and collection of tissue samples were reviewed and approved by the ethics committee of the Henan Provincial People’s Hospital. A total of 100 frozen cholangiocarcinoma samples and paired adjacent normal bile duct tissues were collected from Henan Provincial People’s Hospital from March 2013 to October 2015. Tumor stages were evaluated according to the TNM staging system. The demographic and clinicopathological features of these patients were collected from the hospital database and follow-up was continued for up to 48 months after surgery.

### Cell culture

CCA cell lines HuCCT1 and HuH28 were purchased from Japan Health Science Research Resources Bank, and CCA cell lines SNU-1196, SNU-1079, SNU-308, SNU-245, SNU-478 and SNU-869 were purchased from Korea Cell Line Bank. Human HEK293T was obtained from American Type Culture Collection (ATCC). All cell lines were cultured with RPMI-1640 medium (Invitrogen, USA) supplemented with 10% fetal bovine serum (Hyclone, USA) and 1% PenStrep (100 U/mL Penicilium and 100 μg/mL Streptomycin) in a humid atmosphere containing 5% CO_2_ at 37 °C. Gemcitabine resistant cell lines HuCCT1-Gem and SNU-245-Gem were constructed by exposing to increasing dosages of gemcitabine (Selleck Chemicals, USA) for 8 months, and then persistently cultured in medium containing 10 nmol/l gemcitabine.

### LncRNA microarray and data analysis

The lncRNA expression microarray for two paired cell lines HuCCT1-Gem, HuCCT1, SNU-245-Gem and SNU-245 was performed using Agilent Array platform (Kangchen Bio-tech, Shanghai, China). The differently expressed lncRNAs in HuCCT1-Gem versus HuCCT1, and SNU-245-Gem versus SNU-245 were calculated and identified using the GeneSpring GX v11.5.1 software package (Agilent Technologies, Santa Clara, CA, USA). Different expressed lncRNA was defined as |Log_2_ fold change | ≥2.

### Quantitative real-time polymerase chain reaction (qRT-PCR)

Total RNA from tissue samples and culture cells was extracted using TRIzol reagent (Invitrogen, USA). Complementary DNA was generated by RevertAid First Strand cDNA Synthesis Kit (Thermo Fisher Scientific, USA). The expression of microRNAs were quantified by TaqMan microRNA Reverse Transcription Kit (Applied Biosystem, USA). RT-qPCR was performed using SYBR Premix Ex Taq kit (Takara, Japan) on ABI 7900 Real Time PCR system. GAPDH or U6 RNA was used as internal control. Each sample was done in triplicates, and the primers as listed in Supplementary Table 1.

### Cell viability assay

Cell viability assay was evaluated using CellTiter-Glo Luminescent Cell Viability Assay kit (Promega #G7572) according to the manufacturer’s instruction. In brief, balanced the cells and CellTiter-Glo reagents to room temperature, then mixed thoroughly and incubated in darkroom for 10 min. Then record the luminescence signal on a microplate reader. Each sample was done in triplicates.

### Flow cytometry

Digested the cells with 0.05% trypsin, then washed for three times with phosphate-buffered saline (PBS). Resuspended 1 × 10^6^ cells with binding buffer, then incubated with 5 μL Annexin V-FITC and 10 μL propidium iodide (PI) (Sigma-Aldrich, USA) for 15 min at room temperature avoiding night. The fluorescence signal at 488/530 was recorded by flow cytometry.

### BrdU incorporation assay

Cells were seeded in 6-well plates, then added BrdU (10 μmol/L) and re-incubated for another 4 h. The cells with BrdU incorporated into DNA were detected by incubating with BrdU Mouse mAb (Cell signaling #5292, 1: 1000) at room temperature for 2 h. Then stained with anti-mouse IgG (Alexa Fluor 488 Conjugate) (Cell signaling #4408, 1: 500) and DAPI (Invitrogen, USA) for nucleus. Mounted the cells in LSM 5 Pa Laser Scanning Microscope (Zeiss Germany, Oberkochen, Germany).

### Transwell cell migration and invasion assay

Cells (1 × 10^6^/well) were seeded in the upper chamber (Costar Corp, USA) without serum, and the lower chamber was filled with medium containing 20% FBS. To evaluate cell migration, cells were allowed to translocate toward the lower chamber for 48 h, then cells were fixed by 4% paraformaldehyde for 15 min and stained with crystal violet. To evaluate cell invasion, the filter was pre-coated with Matrigel (BD Biosciences, USA), and cells were allowed to invade through the filter for 48 h, then stained with Giemsa dye. All experiments were carried out in triplicates.

### Immunofluorescence

Cells were fixed by 4% paraformaldehyde for 15 min, then permeabilized with 0.5% Triton X-100. Blocked the cells with 1% gelatin in PBS for 1 h, then incubated with primary antibody β-Catenin (BD610153, BD Biosciences) at 4 °C overnight. Then cells were then incubated with Rhodamine-conjugated goat anti-mouse secondary antibody at room temperature for 1 h. Washed cells with PBS and then incubated with DAPI (Invitrogen, USA) for 30 min at room temperature. Mounted the cells in LSM 5 Pa Laser Scanning Microscope (Zeiss Germany, Oberkochen, Germany).

### Luciferase reporter assay

LINC00665 or the 3′ UTR of BCL9L were cloned into pMIR-REPORT plasmid. Quickchange site-directed mutagenesis kit (Agilent Technologies, USA) was used to generate mutations at the predicted binding sites in LINC00665 and BCL9L. HEK 293 T cells were co-transfected with pMIR-REPORT plasmid containing LINC00665 or the 3′ UTR of BCL9L, miR-424-5p expression plasmid or miR-ctrl, and a renilla luciferase plasmid at a ratio of 2: 2: 1. Dual-Luciferase Reporter Assay System (Promega, USA) was used to evaluate luciferase activity. All assays were done in triplicates.

### RNA in situ hybridization analysis

RNA in situ hybridization analysis was performed according previous reports^44^. In brief, slides were deparaffinized and rehydrated by xylene and an ethanol gradient, then digested with proteinase K for 20 min at 37 °C. The slides were fixed by 4% paraformaldehyde and rehydrated in an ethanol gradient. The 10 pmol digoxin-labeled LINC00665 DNA probe was used to incubate with slides in a humidified hybridization chamber at 50 °C overnight. Slides were washed accordingly and blocked with blocking buffer (Roche, Mannheim, Germany) at room temperature for 30 min, then incubated with anti-Digoxigenin-AP Fab fragments (Roche, Mannheim, Germany, 1: 250 diluted). Then slides were washed accordingly and stained with freshly diluted NBT-BCIP detection solution (Roche, Mannheim, Germany) at 50 °C for 30 min. Images were obtained using a LSM 5 Pa Laser Scanning Microscope (Zeiss Germany, Oberkochen, Germany).

### Plasmid constructs and transfection

LINC00665 and BCL9L expression plasmids were constructed by inserting the cDNA sequence into pCDH-CMV-MCS-EF1-Puro (System Biosciences #CD510B-1) lentiviral vector. The pCDH-CMV-MCS-EF1-Puro empty plasmid was used as empty vector control (EV). Short hairpin RNA (shRNA) constructs for LINC00665 (sh-LINC00665-1 and sh-LINC00665-2) were constructed by cloning the DNA sequence targeting LINC00665 into the pLKO.1 plasmid. Empty pLKO.1 plasmid was used as sh-ctrl. Two short guide RNAs (sgBCL9L-1 and sgBCL9L-2) targeting human BCL9L were designed using the Optimized CRISPR Design web tool (http://crispr.mit.edu/). SgBCL9L-1 and sgBCL9L-2 plasmids were constructed by cloning these two short guide RNAs into lentiCRISPRv2 vector (Addgene, plasmid #52961). SgNC was generated by cloning a sgRNA with none-targeting sequence into the lentiCRISPRv2 vector. The shRNA sequences targeting LINC00665 and sgRNA sequences targeting BCL9L were listed in Supplementary Table 2. The expression plasmid for miR-424-5p was constructed by cloning the mature sequence into pCMV-MIR lentiviral vector (OriGene #PCMVMIR). The empty pCMV-MIR lentiviral plasmid was used as miR-ctrl. For lentivirus transfection, cells were incubated with virus particles overnight with 8 μg/mL polybrene (Sigma-Aldrich, USA). MicroRNA mimics were synthesized by Ribobio (Guangzhou, China) and transient transfection of these microRNA mimics was performed using lipofectamine 3000 (Invitrogen, USA) according to standard protocol.

### Soft agar assay, colony formation assay, and sphere formation assay

Soft agar assay was conducted as previously described^45^. Briefly, cells (8000/well) were seeded in 0.4% top agar in 6-well plates, and cultured for 3 weeks. Then colonies were stained with 1 mg/ml thiazolyl blue tetrazolium bromide (MTT, Sigma-Aldrich, USA) for 3 h. Colony formation was evaluated by seeding 3000 cells per well in 6-well plates, and cultured for 2 weeks without disturbance. Then cells were fixed by 4% paraformaldehyde for 15 min and stained with crystal violet for 2 h at room temperature. Sphere formation was evaluated by seeding cells into 6-well ultra-low attachment plates (Corning, USA) and cultured with DMEM/F12 (Gibco, USA) supplemented with 2% B27, 10 ng/ml EGF and 10 ng/ml FGF. Cells were reseeded at 1000 cells/well once a week to develop secondary or tertiary spheres.

### Pull-down assay with biotinylated miR-424-5p

Cells were transfected with biotinylated wild-type or mutant miR-424-5p, or miR-ctrl. Then collected cell lysates at 48 h post-transfection, and incubated with Dynabeads M-280 Streptavidin (Invitrogen, USA) at 4 °C overnight. Washed the beads as previously reported^46^. The bound RNAs were extracted by TRIzol reagents and the expression of LINC00665 and BCL9L was evaluated by qRT-PCR.

### Western blot

The nucleus and cytoplasmic fractions of CCA cells were prepared as previously described^25^. Cell lysates of the entire cells or nucleus fractions were collected by digesting with RIPA buffer and protease inhibitors (Sigma-Aldrich, USA). Protein concentration was determined by BCA kit (Thermo Fisher, USA). A total of 30 μg protein was loaded on 10% or 15% SDS-PAGE gels and transferred onto nitrocellulose membranes. The membranes were incubated with specific first antibodies and corresponding second antibody. The specific antibodies were listed below: Cleaved Caspase-3 #9664, PCNA #13110; MMP-3 #14351, ZEB1 #70512, E-Cadherin #3195, Vimentin #5741, Oct-4 #2750, LIN28A #3695, Nanog #3580, Sox2 #3579, β-Catenin #8480, Phospho-β-Catenin (Ser33/37/Thr41) # 9561, Phospho-GSK-3β (Ser9) #5558, GSK-3β#12456, c-Myc #9402, Survivin #2803, Lamin B1 #12586, and GAPDH #5174 were purchased from Cell Signaling Technology with a work concentration at 1: 1000. BCL9L #ab233736 was purchased from Abcam with a work concentration at 1: 500. The second antibodies were goat anti-rabbit IgG HRP-linked antibody (Cell signaling #7074; 1:4000) and sheep anti-mouse IgG-HRP (GE/Amershan #NXA931; 1: 5000).

### Tumor xenograft model

All animal experiments were approved by the Animal Care and Experimental Committee of Henan Provincial People’s Hospital. HuCCT1-Gem cells (2 × 10^6^) transduced with sh-LIN00665-1, miR-424-5p, sgBCL9L-1, sg-NC, miR-ctrl or sh-ctrl, or HuCCT1 cells transduced with LINC00665 expression lentivirus or EV, were subcutaneously injected in nude mice for two weeks before visible tumor achieved (6 mm in each dimension). Then the mice were grouped into the following group: (1) Gemcitabine group received 100 mg/kg gemcitabine intraperitoneally every 3 days for 3 weeks; (2) Vehicle group received an equal volume of PBS at the same time. Tumor volume was monitored every three day by caliper and calculated according to the formula (length × width^2^)/2. All mice were sacrificed at the end of gemcitabine treatment and tumors were dissected out and weighed.

### Statistical analysis

Data were analyzed by IBM SPSS Statistics 22.0 or GraphPad Prism 8.0 software. Kaplan–Meier method was used to plot overall survival and recurrent-free survival and evaluated by Logrank test. Gemcitabine IC50 was measured by nonlinear regression model of GraphPad Prism 8.0. Two-tailed Student’s t-test or nonparametric Mann–Whitney *u*-test was used to calculate statistical significance between two comparator groups. The difference of multiple groups was analyzed by one-way analysis. Pearson chi-square was used to analyze the associations between LINC00665 expression and clinicopathological variables. All data was shown as mean ± standard deviation (x ± s.d) with at least three replicates. *P* ≤ 0.05 was considered statistically significant.

## Supplementary information

Supplementary Figure legends

Supplementary Figure 1

Supplementary Figure 2

Supplementary Figure 3

Supplementary Figure 4

Supplementary Figure 5

Supplementary Figure 6

Supplementary Figure 7

Supplementary Table 1

Supplementary Table 2

Supplementary Table 3

Supplementary Table 4

Supplementary Table 5

Supplementary Table 6

## Data Availability

The datasets used and/or analyzed during the current study are available from the corresponding author on reasonable request.
