# Supplementary material for: Long non-coding RNA LINC00665 promotes gemcitabine resistance of Cholangiocarcinoma cells via regulating EMT and stemness properties through miR-424-5p/BCL9L axis
Source: Cell Death Dis. 2021 Jan 12;12(1):72. doi: 10.1038/s41419-020-03346-4 (PMC7803957; doi:10.1038/s41419-020-03346-4)
Supplement: Supplementary file 1 — Supplementary Figure legends [file 41419_2020_3346_MOESM1_ESM.docx]

**Supplementary Figure Legends**

Supplementary Figure 1. Resistant CCA cell lines have enhanced gemcitabine tolerance in soft agar. A-B, HuCCT1-Gem, HuCCT1, SNU-245-Gem or SNU-245 cells were seeded in soft agar (8000 cells/well), then treated with 0, 5, 10 or 20 nM gemcitabine. Represent image of plates (A) and number of colonies (B) were shown. C, CCA samples and paired adjacent-normal tissues were subjected to in situ hybridization analysis for the expression of LINC00665. Represent images were shown. Scale bars = 10 μm.

Supplementary Figure 2. LINC00665 expression is dramatically downregulated by shRNAs targeting LINC00665. HuCCT1-Gem and SNU-245-Gem cells were transduced with sh-LINC00665-1, sh-LINC00665-2 or sh-ctrl, then relative expression of LINC00665 was evaluated by qRT-PCR. ^***^*P* ≤0.05.

Supplementary Figure 3. Transducing with LINC00665 expression lentivirus significantly increased the expression level of LINC00665. HuCCT1 and SNU-245 cells were transduced with LINC00665 expression lentivirus or EV, then relative expression of LINC00665 was evaluated by qRT-PCR. ^***^*P* ≤0.05.

Supplementary Figure 4. Gemcitabine-resistant CCA cells exert enhanced EMT and stemness properties. A-E, HuCCT1-Gem, HuCCT1, SNU-245-Gem or SNU-245 cells were used for sphere formation assay (A-B), transwell cell migration and invasion (C-E) assay. F-J, HuCCT1 and SNU-245 cells were transduced with LINC00665 expression lentivirus or EV control, then cells were used for used for sphere formation assay (F-G), transwell cell migration and invasion (H-J) assay. Scale bars = 100 μm for sphere formation assay, and scale bars = 50 μm for transwell cell migration and invasion assay. ^***^*P* ≤0.05.

Supplementary Figure 5. The influence of LINC00665 knockdown on downstream target genes and pathway regulators of Wnt/β-Catenin signaling. HuCCT1-Gem and SNU-245-Gem cells were transduced with sh-LINC00665-1, sh-LINC00665-2 or sh-ctrl, then the expression of downstream target genes (A-B) and pathway regulators (C-D) of Wnt/β-Catenin signaling was evaluated by qRT-PCR. ^***^*P* ≤0.05.

Supplementary Figure 6. MiR-424-5p is confirmed to interact with LINC00665 and BCL9L both in CCA cells. A-B, HuCCT1 and SNU-245 cells were transduced with LINC00665 expression lentivirus or EV, then relative expression of indicated microRNAs were evaluated by qRT-PCR. C-D, HuCCT1 and SNU-245 cells were transient transfected with indicated microRNA mimics, then relative BCL9L expression was evaluated by qRT-PCR. E, HuCCT1-Gem and SNU-245-Gem cells were transduced with miR-424-5p lentivirus expression vector or miR-ctrl, then relative miR-424-5p expression was evaluated by qRT-PCR. F, HuCCT1-Gem and SNU-245-Gem cells were transduced with wt or mt BCL9L lentivirus expression vector or empty vector ctrl (Ctrl), then relative BCL9L expression was evaluated by qRT-PCR. ^***^*P* ≤0.05.

Supplementary Figure 7. Enforced miR-424-5p expression or silencing BCL9L increased gemcitabine-induced apoptosis and suppressed tumor xenograft growth of resistant CCA cells. A-B, HuCCT1-Gem and SNU-245-Gem cells transduced sgBCL9L-1, sgBCL9L-2, sgNC, miR-424-5p or miR-ctrl were treated with 10 nM gemcitabine for 3 days, then cells were stained with Annexin V-FITC and PI for flow cytometry (A). The Annexin V-FITC and PI positive subsets (B) were shown. C-E, HuCCT1-Gem cells (2×10^6^) transduced with miR-424-5p, sgBCL9L-1 or empty lentivirus control were subcutaneous injected into nude mice, then treated with gemcitabine (100 mg/kg) or equal volume of PBS for three weeks. Tumor growth curves (C), represent images (D) and tumor weight (E) were shown. ^***^*P* ≤0.05.

Supplementary Table 1. Primers of indicated genes used in qRT-PCR.

Supplementary Table 2. shRNA sequences targeting LINC00665 and sgRNA sequences targeting BCL9L.

Supplementary Table 3. Dysregulated lncRNAs in gemcitabine resistant CCA cell lines identified by lncRNA microarray.

Supplementary Table 4. Dysregulated lncRNAs identified in HuCCT1-Gem and SNU-245-Gem cells both.

Supplementary Table 5. Conservative microRNA targeting sites for BCL9L predicted by TargetScanHuman 7.2.

Supplementary Table 6. MicroRNAs predicted to interact with LINC00665 and BCL9L both.
