# Supplementary figures and images for: Long non-coding RNA LINC00665 promotes gemcitabine resistance of Cholangiocarcinoma cells via regulating EMT and stemness properties through miR-424-5p/BCL9L axis
Source: Cell Death Dis. 2021 Jan 12;12(1):72. doi: 10.1038/s41419-020-03346-4 (PMC7803957; doi:10.1038/s41419-020-03346-4)

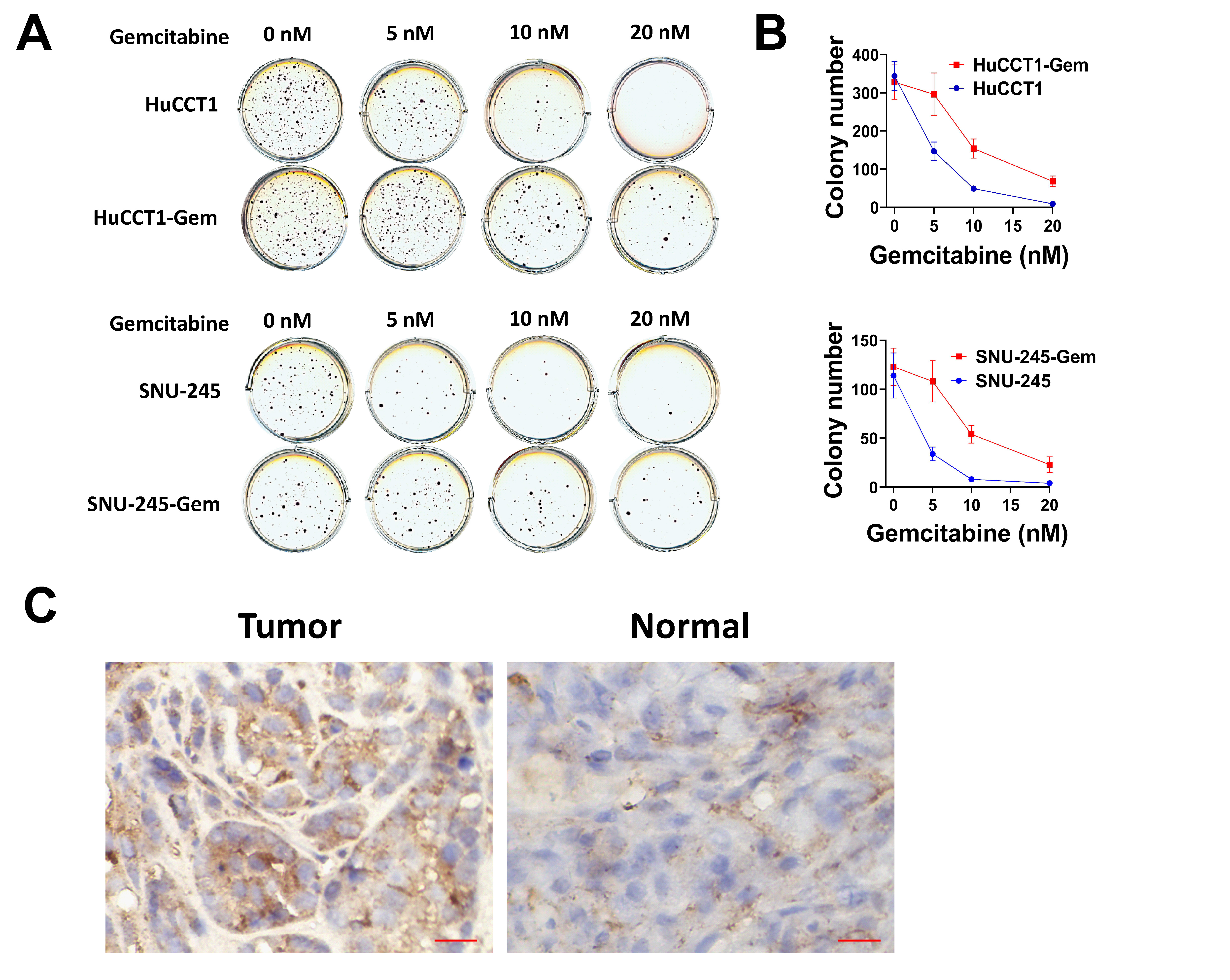

Supplement: Supplementary file 2 — Supplementary Figure 1 [file 41419_2020_3346_MOESM2_ESM.jpg]

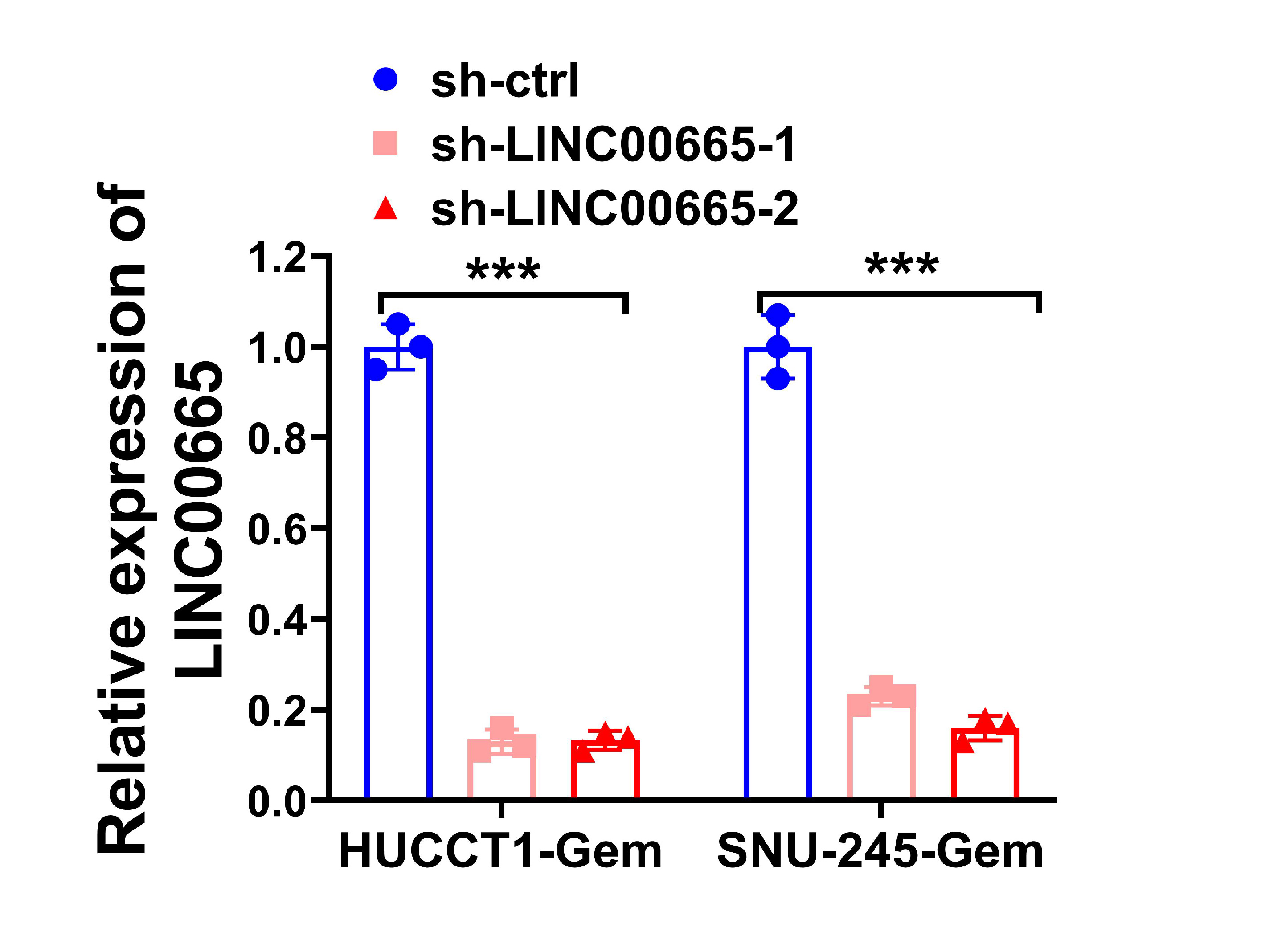

Supplement: Supplementary file 3 — Supplementary Figure 2 [file 41419_2020_3346_MOESM3_ESM.jpg]

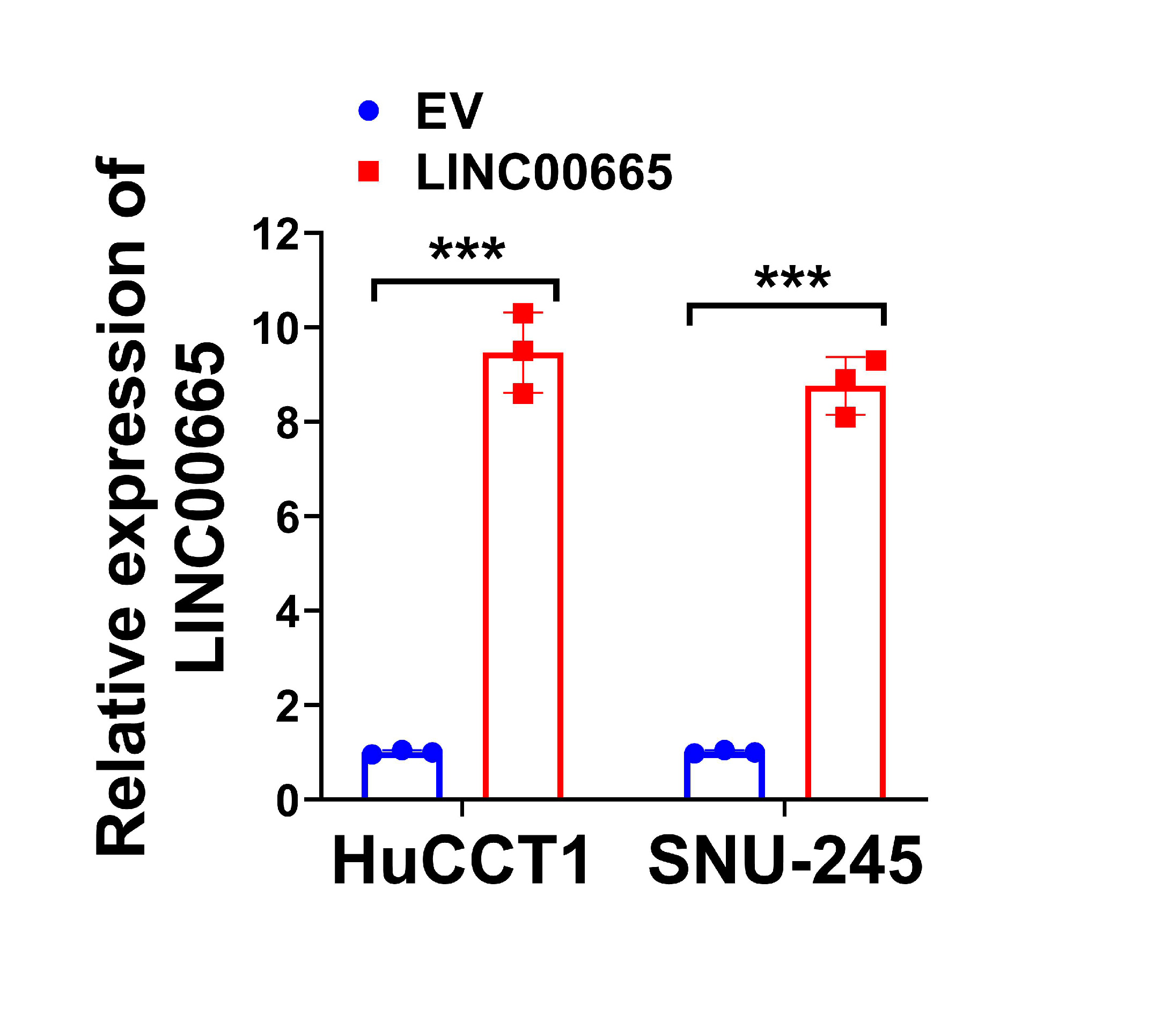

Supplement: Supplementary file 4 — Supplementary Figure 3 [file 41419_2020_3346_MOESM4_ESM.jpg]

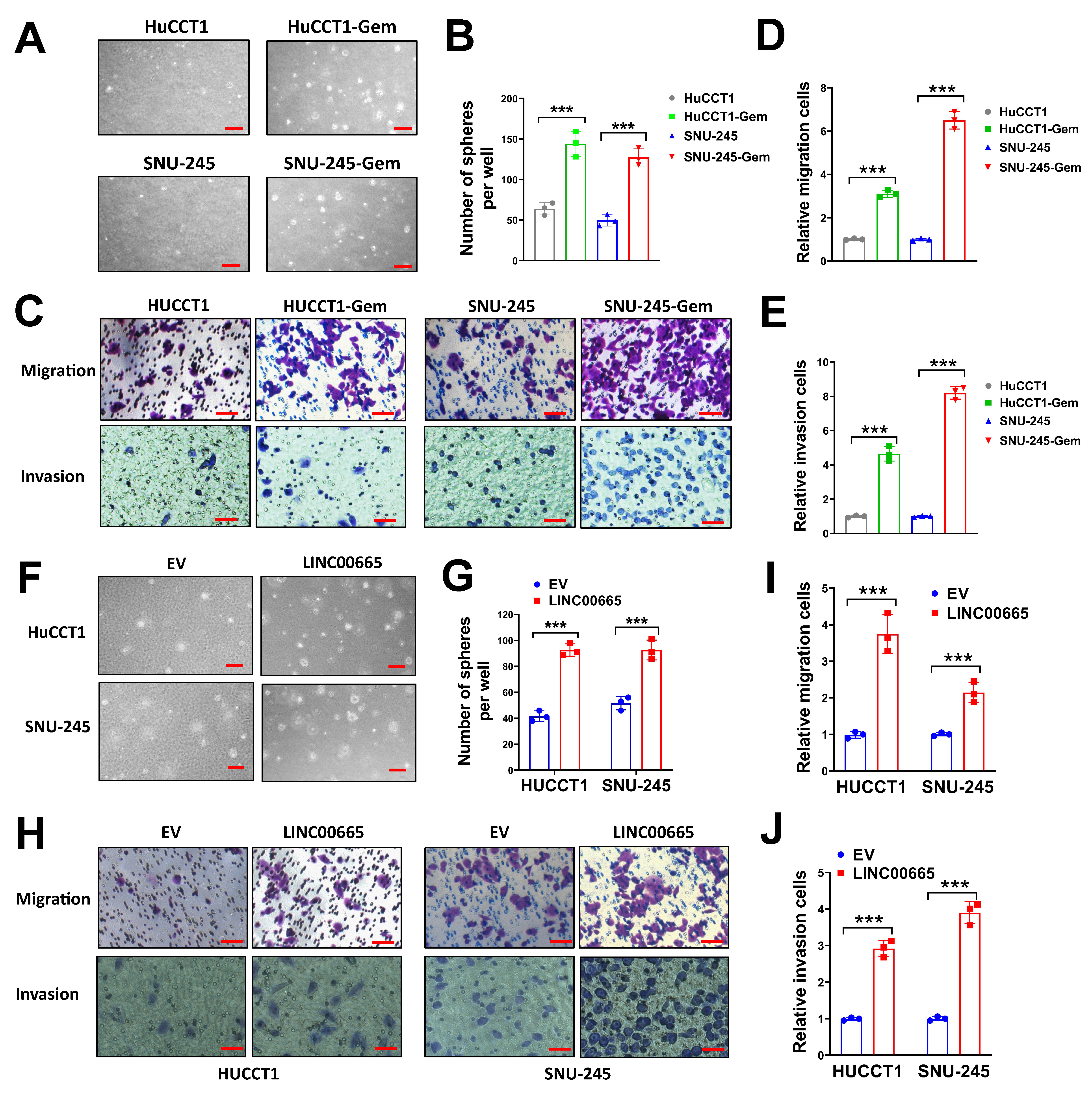

Supplement: Supplementary file 5 — Supplementary Figure 4 [file 41419_2020_3346_MOESM5_ESM.jpg]

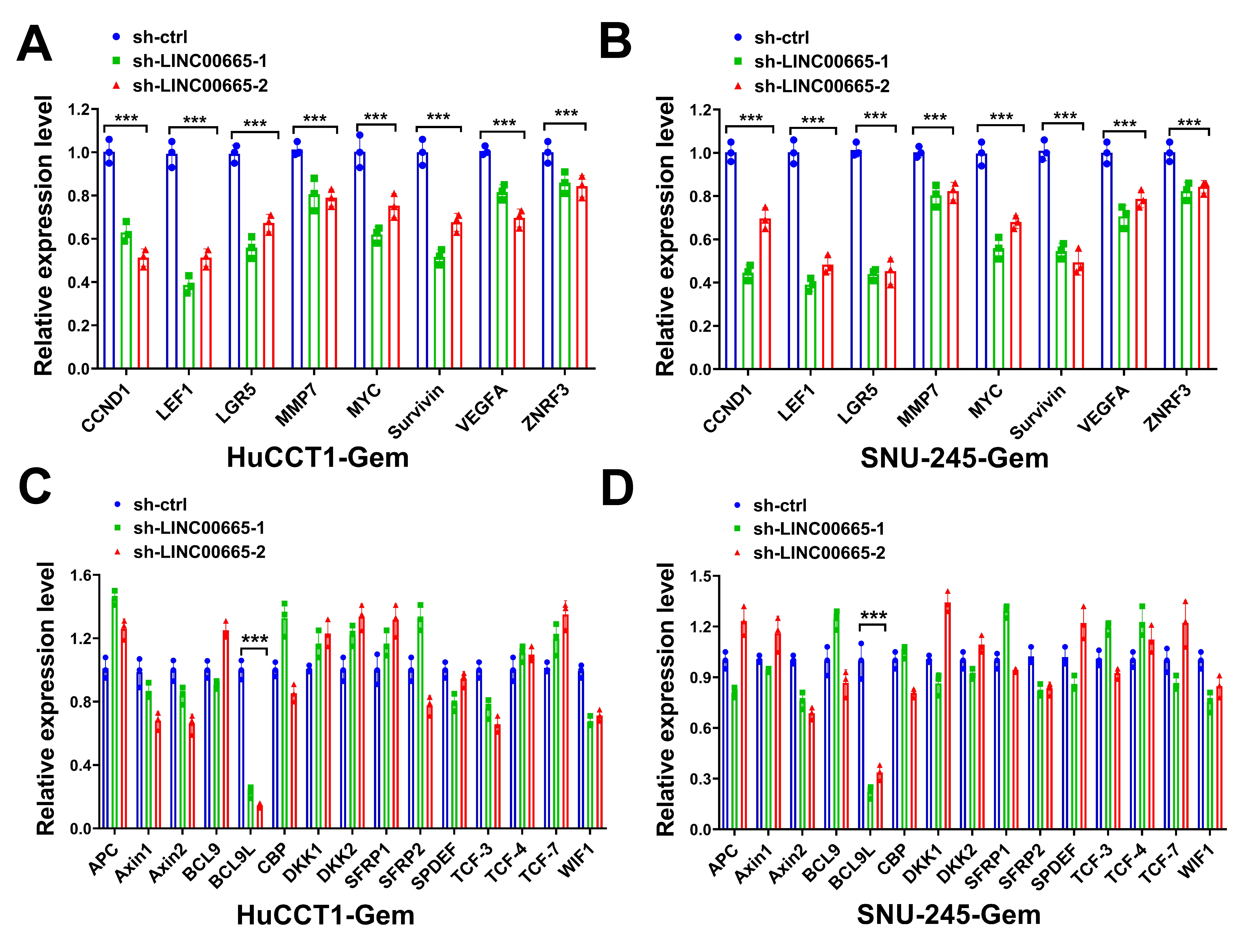

Supplement: Supplementary file 6 — Supplementary Figure 5 [file 41419_2020_3346_MOESM6_ESM.jpg]

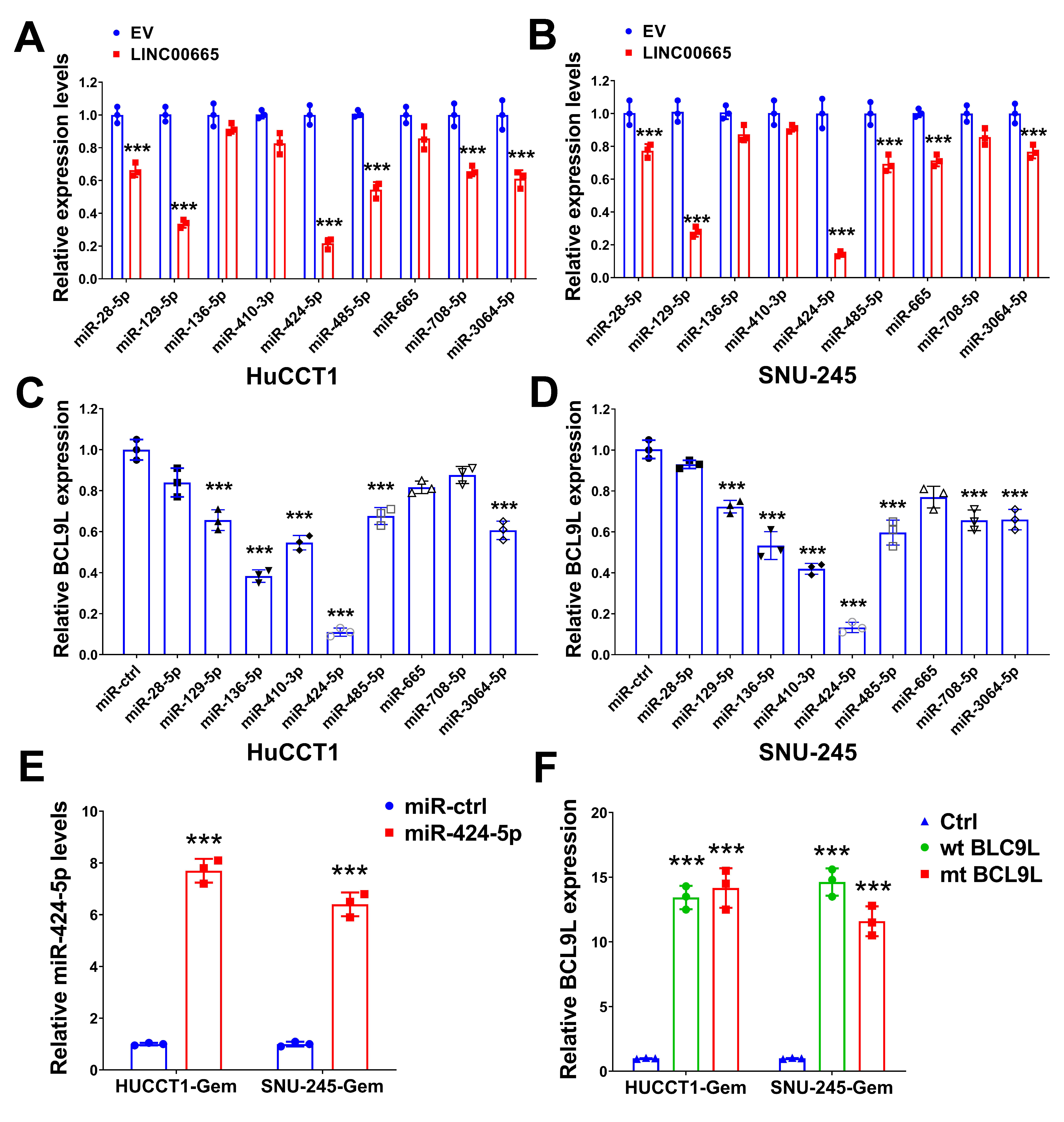

Supplement: Supplementary file 7 — Supplementary Figure 6 [file 41419_2020_3346_MOESM7_ESM.jpg]

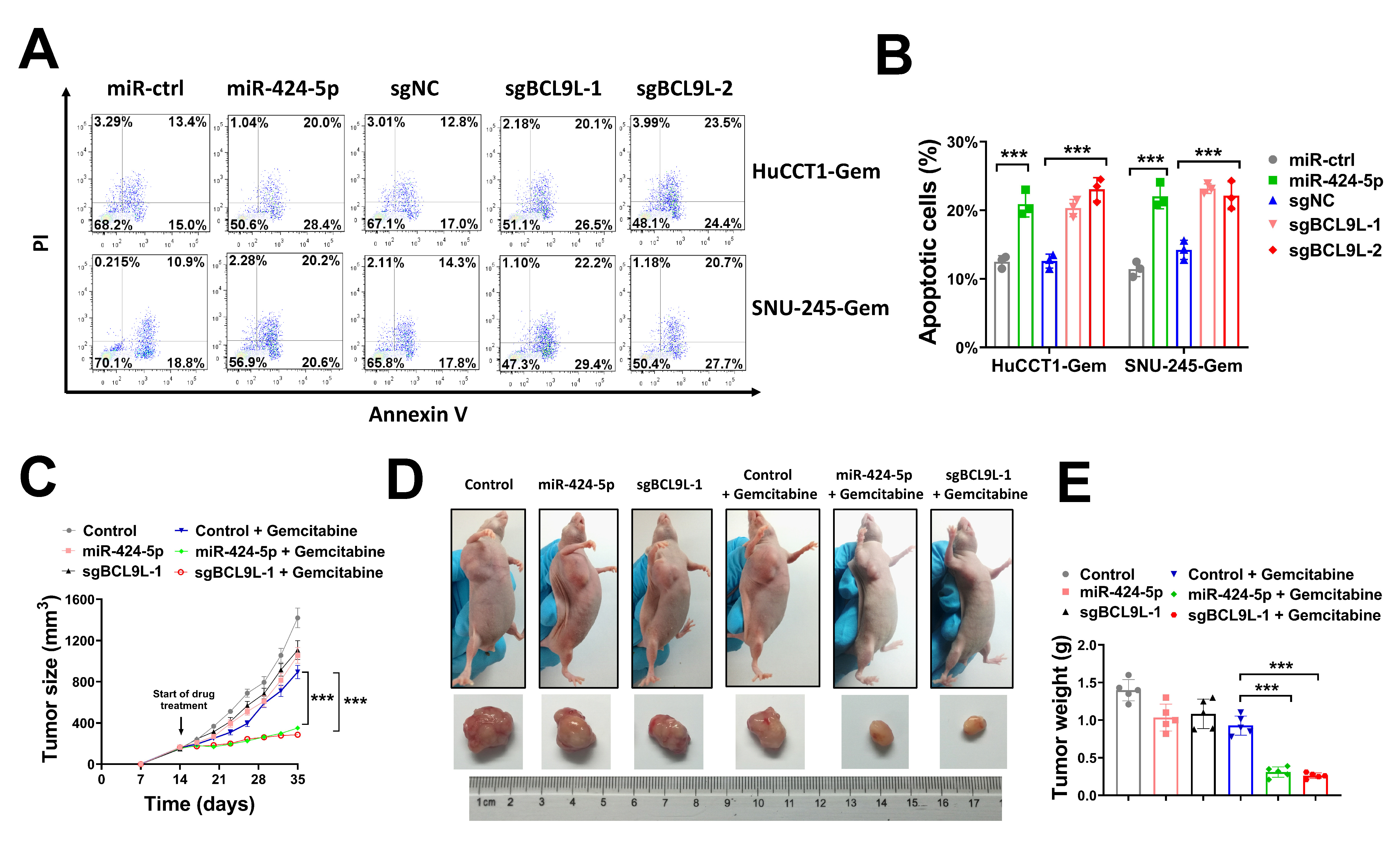

Supplement: Supplementary file 8 — Supplementary Figure 7 [file 41419_2020_3346_MOESM8_ESM.jpg]
