# Supplementary material for: Long non-coding RNA LINC00665 promotes gemcitabine resistance of Cholangiocarcinoma cells via regulating EMT and stemness properties through miR-424-5p/BCL9L axis
Source: Cell Death Dis. 2021 Jan 12;12(1):72. doi: 10.1038/s41419-020-03346-4 (PMC7803957; doi:10.1038/s41419-020-03346-4)
Supplement: Supplementary file 9 — Supplementary Table 1 [file 41419_2020_3346_MOESM9_ESM.docx]

**Supplementary Table 1. Primers of indicated genes used in qRT-PCR.**

| **Gene name** | **Sense primer** | **Anti-senser primer** |
| --- | --- | --- |
| APC | TTCGATTGCCAGCTCCGTTCAG | TTGAGGAGGTGGTGGAGGTGTT |
| AXIN1 | CCGACTTGCTGGACTTCTGGTT | TTGGTGGCTGGCTTGGTCTG |
| AXIN2 | TACCGGAGGATGCTGAAGGC | CCACTGGCCGATTCTTCCTT |
| BCL9 | GGACCCGACCACCTTGACCATA | CCACTTGTTCCTGCTTCCTCCT |
| BCL9L | GCCTGTCTGCTGCTGAGTGATT | ACCGCCTCTTCCTCCTTTCCTT |
| CBP | AACGCATCGGTCGCTTCCTG | GCAGCATCGTCTTCGGTCAGTC |
| CCND1 | TTCAAATGTGTGCAGAAGGA | GGGATGGTCTCCTTCATCTT |
| DKK1 | AACAGCTATCCAAATGCAG | TCACAGGGGAGTTCCATAAA |
| DKK2 | TCCTGCTGCCTGCTCCTACT | CTTCTTACTGCCGCCGAATGC |
| E-cadherin | TACACTGCCCAGGAGCCAGA | TAATCCGGACACTGGTGCCA |
| GAPDH | TGCACCACCAACTGCTTAGC | GGCATGGACTGTGGTCATGAG |
| LEF1 | GCACGAACCCTTCCAACTCTCC | CTGTAATCTCCGCTCCGCTGTG |
| LGR5 | TGTGCTCCTGTCCTTGCCTGT | AGGTGAAGACGCTGAGGTTGGA |
| Lin28 | AAAGGAGACAGGTGCTAC | ATATGGCTGATGCTCTGG |
| LINC00665 | GGTGGATCACGAGGTCAGGAGA | GCTCACTGCAAGCTCTGCCTAC |
| MMP3 | TTCCGCCTGTCTCAAGATGATAT | AAAGGACAAAGCAGGATCACAGTT |
| MMP7 | TGCGACTCACCGTGCTGTGT | GTCCTGAGCCTGTTCCCACTGT |
| MYC | ACCAGAGAAACCTAACAGTGC | CTCTTTCATTTCGGCCAGTTC |
| Nanog | AAGAACTCTCCAACATCCTGAAC | CCTTCTGCGTCACACCATT |
| Oct4 | CGCCGTATGAGTTCTGTG | GGTGATCCTCTTCTGCTTC |
| SFRP1 | ACCTCAGTGCGTGGACATCC | CGGCGTGGCAGTTCTTGTTG |
| SFRP2 | GCCACGGCATCGAATACCAGAA | CGAAGAGCGAGCACAGGAACTT |
| Sox2 | AGTTGGACAGGGAGATGGC | AACCTTCCTTGCTTCCACG |
| SPDEF | CGACAGCGAGGTGGACTCATCA | GTTCATGGCGGGACGGTTCTTG |
| Survivin | TCAACAAGCAAGCGAGACTCCT | GCAAACGCAGCCCTCTTCAAAC |
| TCF-3 | AGGTGTCAGGTGTGGTTGGAGA | AGGTGTGGATGTGGATGAAGCC |
| TCF-4 | GCAGAAGGCAGAGCGTGAGAAG | GGTGGAGGATCAGGAGCTTGGT |
| TCF-7 | CGCTGCCATCAACCAGATCCT | CCTCGACCGCCTCTTCTTCTTC |
| U6 | CGCTTCGGCAGCACATATACTA | CGCTTCACGAATTTGCGTGTCA |
| VEGFA | GACGGACAGACAGACAGACACC | GAAGCGAGAACAGCCCAGAAGT |
| Vimentin | AGTCCACTGAGTACCGGAGAC | CATTTCACGCATCTGGCGTTC |
| WIF1 | GGTACGAAGCCAGCCTCATACA | TCAGATGTCGGAGTTCACCAGA |
| ZEB1 | GTTACCAGGGAGGAGCAGTGAAA | GACAGCAGTGTCTTGTTGTTGTAGAAA |
| ZNRF3 | GCTGAACCAGGGCTCTGAAGAC | ACCAAGGAGACCACGACGAAGA |
