# Supplementary material for: Long non-coding RNA LINC00665 promotes gemcitabine resistance of Cholangiocarcinoma cells via regulating EMT and stemness properties through miR-424-5p/BCL9L axis
Source: Cell Death Dis. 2021 Jan 12;12(1):72. doi: 10.1038/s41419-020-03346-4 (PMC7803957; doi:10.1038/s41419-020-03346-4)
Supplement: Supplementary file 10 — Supplementary Table 2 [file 41419_2020_3346_MOESM10_ESM.docx]

| **Name** | **Sequence** |
| --- | --- |
| SgBCL9L-1 | ACACAGCGCCGCTTACTCCG |
| sgBCL9L-2 | AGCCGTACAGTGGGGACGAA |
| sgNC | ACGGAGGCTAAGCGTCGCAA |
| sh-LINC00665-1 | AATAGCCCAAGACTGAGGACTCACA |
| sh-LINC00665-2 | TGTGAGTCCTCAGTCTTGGGCTATT |

**Supplementary Table 2. shRNA sequences targeting LINC00665 and sgRNA sequences targeting BCL9L.**
