# Supplementary material for: Long non-coding RNA LINC00665 promotes gemcitabine resistance of Cholangiocarcinoma cells via regulating EMT and stemness properties through miR-424-5p/BCL9L axis
Source: Cell Death Dis. 2021 Jan 12;12(1):72. doi: 10.1038/s41419-020-03346-4 (PMC7803957; doi:10.1038/s41419-020-03346-4)
Supplement: Supplementary file 11 — Supplementary Table 3 [file 41419_2020_3346_MOESM11_ESM.docx]

**Supplementary Table 3. Dysregulated lncRNAs in gemcitabine resistant CCA cell lines identified by lncRNA microarray.**

| **HuCCT1-Gem** | | | **SNU-245-Gem** | | |
| --- | --- | --- | --- | --- | --- |
| **Symbol** | **log2 FoldChange** | **p Value** | **Symbol** | **log2 FoldChange** | **p Value** |
| AL161431.1 | 20.43608271 | 9.50E-38 | UCA1 | 19.38596648 | 2.25E-07 |
| AC092336.1 | 19.85015615 | 2.52E-35 | AC013275.2 | 14.84760144 | 3.33E-10 |
| AL773572.1 | 18.29193981 | 2.50E-23 | RP11-532F12.5 | 14.45535715 | 5.84E-12 |
| AC108752.1 | 17.90078474 | 2.24E-12 | AC016735.1 | 14.04944315 | 3.07E-11 |
| LINC01057 | 10.28211252 | 1.58E-09 | SCARNA15 | 13.11263844 | 1.41E-05 |
| UCA1 | 9.612944835 | 5.26E-10 | ZNF888 | 12.65559397 | 2.35E-05 |
| AFAP1-AS1 | 7.676528867 | 1.24E-21 | LINC01123 | 12.17976465 | 2.68E-10 |
| FAM83H-AS1 | 7.210580906 | 2.74E-08 | RP11-284F21.7 | 11.97045906 | 4.80E-11 |
| LINC02441 | 7.151151114 | 4.12E-06 | PVT1 | 11.51122444 | 1.88E-09 |
| PVT1 | 6.979804296 | 2.12E-15 | RP11-429J17.5 | 11.4819038 | 3.53E-07 |
| LINC01559 | 6.89882315 | 4.85E-16 | LINC00511 | 11.25183086 | 4.16E-10 |
| BLACAT1 | 6.527494083 | 1.43E-11 | LINC01559 | 11.14589867 | 4.46E-06 |
| LINC01833 | 6.418589225 | 5.21E-10 | RP11-334E6.12 | 11.09959987 | 2.05E-07 |
| LINC01956 | 6.265149793 | 1.39E-10 | KB-1615E4.2 | 10.89349417 | 2.67E-08 |
| CASC8 | 6.255544111 | 1.78E-07 | LINC00665 | 10.86192752 | 1.12E-05 |
| LINC00665 | 5.93103002 | 1.44E-11 | RP1-102K2.8 | 10.81213999 | 4.76E-06 |
| AC022028.2 | 5.917543646 | 1.42E-10 | RP3-416H24.1 | 10.79041552 | 3.32E-07 |
| RP11-462L8.1 | 5.867049353 | 3.12E-06 | VCAN-AS1 | 10.7591372 | 1.12E-04 |
| AC097478.1 | 5.582032041 | 9.42E-08 | AC006273.5 | 10.70519822 | 4.10E-09 |
| LINC00707 | 5.5565671 | 8.01E-07 | LINC01057 | 10.51173036 | 2.25E-09 |
| HOXB-AS3 | 5.469783891 | 3.15E-05 | MFI2-AS1 | 10.50239645 | 1.05E-07 |
| AC004231.1 | 5.438840923 | 1.23E-09 | RP11-284F21.10 | 10.49836107 | 1.85E-09 |
| LINC00152 | 5.438840923 | 1.39E-10 | RP11-44N21.1 | 10.36448372 | 1.43E-09 |
| LINC01234 | 5.39289273 | 8.01E-07 | FAM83H-AS1 | 10.30082987 | 3.91E-08 |
| RP11-304L19.12 | 5.39289273 | 1.42E-10 | COLCA1 | 10.16758677 | 3.33E-08 |
| LINC02154 | 5.313676849 | 3.96E-07 | MIR4435-1HG | 9.859891619 | 3.35E-09 |
| LINC00113 | 5.29806904 | 4.87E-07 | RP11-350N15.4 | 9.803860837 | 3.61E-06 |
| AC011352.3 | 5.257803977 | 1.17E-06 | SPACA6P | 9.653035652 | 4.93E-07 |
| LINC02041 | 5.224451218 | 4.77E-10 | LINC01106 | 9.635747002 | 8.59E-10 |
| LINC00941 | 5.171772012 | 8.50E-16 | FAM201A | 9.443322247 | 2.38E-09 |
| LINC00494 | 5.134857744 | 3.38E-11 | RP11-404P21.3 | 9.436804672 | 1.62E-07 |
| RP11-284F21.9 | 5.126360775 | 9.42E-08 | BX470102.3 | 9.257720978 | 3.84E-07 |
| AC105219.4 | 5.066932222 | 8.04E-11 | ZFAS1 | 9.095011843 | 6.72E-08 |
| APCDD1L-AS1 | 4.972590842 | 9.07E-07 | CTD-2228K2.7 | 9.028609199 | 1.19E-06 |
| CLDN10-AS1 | 4.943900269 | 2.69E-04 | RP11-554I8.2 | 8.860524656 | 4.23E-05 |
| AL512274.1 | 4.845738393 | 5.91E-20 | RP11-2C24.4 | 8.855134678 | 4.42E-08 |
| MIR200CHG | 4.837422557 | 3.05E-03 | LINC00152 | 8.798109445 | 4.32E-07 |
| AP005233.2 | 4.813089963 | 1.02E-06 | PAX8-AS1 | 8.763288732 | 8.92E-05 |
| AL365181.3 | 4.803247608 | 1.35E-23 | AC009133.12 | 8.749870636 | 7.17E-07 |
| FOXD3-AS1 | 4.788779729 | 2.52E-07 | CRNDE | 8.706040175 | 1.83E-06 |
| AC021151.1 | 4.785010118 | 9.79E-06 | CTD-2012K14.4 | 8.650636822 | 1.34E-04 |
| AC125616.1 | 4.784655828 | 9.60E-05 | RP11-496I9.1 | 8.650601626 | 5.46E-07 |
| AC013275.1 | 4.775549564 | 8.96E-16 | DYNLL1-AS1 | 8.613662351 | 8.09E-07 |
| AC069120.3 | 4.736940481 | 3.71E-08 | AC004540.5 | 8.607808748 | 5.32E-06 |
| HOXB-AS4 | 4.72688327 | 2.01E-03 | AL162759.1 | 8.517168184 | 2.59E-06 |
| AC090192.2 | 4.711260083 | 4.35E-07 | AC159540.1 | 8.468345796 | 3.23E-07 |
| GRM5-AS1 | 4.698430655 | 1.48E-05 | RP11-462L8.1 | 8.381499076 | 4.46E-06 |
| AC080037.1 | 4.696297623 | 2.33E-07 | RP11-465B22.8 | 8.371054173 | 9.24E-07 |
| HOXA-AS3 | 4.690441781 | 7.13E-07 | RP11-675F6.3 | 8.299935913 | 7.87E-05 |
| LINC01436 | 4.678941295 | 5.80E-08 | ZEB1-AS1 | 8.254345058 | 5.42E-07 |
| LINC01105 | 4.621094722 | 1.74E-03 | RP11-304L19.12 | 8.23753704 | 8.06E-05 |
| AC147651.1 | 4.605134951 | 5.30E-10 | RP11-510M2.5 | 8.184890512 | 5.96E-04 |
| LINC02182 | 4.590427251 | 1.05E-06 | RP11-235E17.6 | 8.135947759 | 6.16E-07 |
| AL049555.1 | 4.554448637 | 6.93E-20 | RP4-565E6.1 | 8.009300319 | 1.50E-07 |
| LHFPL3-AS2 | 4.544514104 | 1.93E-10 | CTD-3128G10.6 | 7.946082509 | 2.76E-07 |
| AL049629.1 | 4.530022605 | 1.88E-10 | RP11-54O7.17 | 7.931860941 | 4.48E-07 |
| AC148476.1 | 4.500479943 | 1.77E-07 | RP11-524D16__A.3 | 7.92650804 | 6.76E-06 |
| AC126768.2 | 4.500267354 | 3.08E-05 | KB-1615E4.3 | 7.881348609 | 3.42E-04 |
| AC105118.1 | 4.49981142 | 2.04E-06 | HOXB-AS3 | 7.813976987 | 4.50E-05 |
| AC078909.2 | 4.490293011 | 6.47E-10 | LINC02212 | 7.813274962 | 1.58E-06 |
| AC245884.9 | 4.464826604 | 8.27E-07 | MNX1-AS1 | 7.727580843 | 1.06E-17 |
| MNX1-AS1 | 4.40930659 | 7.42E-18 | LINC00883 | 7.678248299 | 3.69E-06 |
| LINC01121 | 4.39750261 | 1.34E-10 | RP11-112J3.16 | 7.647414159 | 2.14E-07 |
| SLCO4A1-AS1 | 4.389985292 | 4.92E-11 | SNHG7 | 7.591472799 | 1.41E-05 |
| LINC01977 | 4.37905192 | 1.04E-10 | FAM66C | 7.582145905 | 2.18E-07 |
| SSTR5-AS1 | 4.353065108 | 1.03E-06 | AGAP2-AS1 | 7.530289535 | 8.47E-06 |
| AC026368.1 | 4.352175271 | 1.87E-10 | RP11-701H24.4 | 7.491158229 | 5.23E-06 |
| AL136964.1 | 4.341077024 | 1.24E-06 | PWAR6 | 7.481244368 | 4.84E-06 |
| LINC02313 | 4.290585293 | 2.35E-07 | SOX9-AS1 | 7.456956887 | 4.86E-05 |
| LINC00511 | 4.273534544 | 9.24E-24 | ZNF528-AS1 | 7.439388569 | 2.46E-04 |
| RNF144A-AS1 | 4.268822199 | 3.69E-04 | RP11-757F18.5 | 7.400597456 | 4.20E-06 |
| AC010931.2 | 4.26555213 | 8.59E-08 | RP11-394O4.5 | 7.397321582 | 5.98E-05 |
| KCCAT198 | 4.26324237 | 5.00E-06 | SZT2-AS1 | 7.347783224 | 7.88E-06 |
| LINC01615 | 4.260429721 | 2.49E-07 | RP13-890H12.2 | 7.281511818 | 1.85E-06 |
| AC087525.1 | 4.252301534 | 3.61E-05 | RP11-196G18.22 | 7.26418222 | 3.67E-06 |
| LINC00460 | 4.219129453 | 3.10E-06 | AC103702.2 | 7.255468791 | 2.64E-07 |
| AC105219.2 | 4.207109191 | 1.59E-07 | RP11-284F21.9 | 7.231475074 | 1.63E-06 |
| AC103564.1 | 4.189068685 | 2.09E-04 | CTD-2047H16.2 | 7.185208065 | 2.38E-06 |
| AL445647.1 | 4.180318453 | 2.74E-04 | LINC01021 | 7.145135709 | 4.29E-04 |
| LINC01829 | 4.176618385 | 2.23E-07 | LINC01833 | 7.131765806 | 5.21E-10 |
| AL359694.2 | 4.16584435 | 4.54E-07 | PSORS1C3 | 7.122962751 | 2.65E-05 |
| ELFN2 | 4.1158441 | 3.38E-11 | AC144833.1 | 7.120072137 | 7.69E-06 |
| MNX1-AS2 | 4.102861902 | 5.77E-08 | CD27-AS1 | 7.113259488 | 1.20E-05 |
| AC004080.1 | 4.088823099 | 3.44E-05 | RP11-490M8.1 | 7.106570837 | 1.31E-05 |
| AC025154.2 | 4.08796369 | 1.23E-09 | RP11-134G8.10 | 7.085012509 | 2.79E-06 |
| LINP1 | 4.084831607 | 6.37E-06 | LINC01420 | 7.060742203 | 9.29E-06 |
| AC138904.1 | 4.073302834 | 1.27E-07 | NRSN2-AS1 | 7.035298124 | 5.12E-06 |
| AL583722.1 | 4.059311944 | 1.02E-07 | LINC00941 | 7.03454806 | 2.13E-04 |
| EVX1-AS | 4.034831173 | 3.15E-05 | RP11-268J15.5 | 7.026738858 | 4.12E-05 |
| AP000757.2 | 4.021829716 | 1.14E-07 | ACTA2-AS1 | 7.023704962 | 1.52E-04 |
| SOX21-AS1 | 4.018092618 | 1.81E-04 | RP11-589P10.5 | 7.020460059 | 6.72E-05 |
| AC010487.2 | 4.014268916 | 1.20E-07 | RP11-161M6.2 | 6.995102775 | 6.12E-05 |
| AC005381.1 | 4.007329275 | 2.65E-03 | RP11-809O17.1 | 6.968660854 | 2.89E-06 |
| AC069120.1 | 3.994317113 | 2.08E-07 | RP11-244F12.3 | 6.961323725 | 1.06E-05 |
| AL033527.3 | 3.991013405 | 1.27E-07 | PCAT6 | 6.922002334 | 2.43E-05 |
| AC099792.1 | 3.986826566 | 1.16E-04 | H1FX-AS1 | 6.916168381 | 2.24E-04 |
| AC003965.2 | 3.986043167 | 1.98E-07 | PRR7-AS1 | 6.90762319 | 2.66E-06 |
| AL451069.1 | 3.981832794 | 4.18E-05 | CTD-3252C9.4 | 6.89893711 | 4.41E-05 |
| AL138760.1 | 3.980350766 | 3.89E-05 | HOXB-AS4 | 6.853980742 | 1.91E-03 |
| LHFPL3-AS1 | 3.962805484 | 4.71E-04 | RP5-882C2.2 | 6.843556596 | 9.03E-06 |
| AL596223.2 | 3.956399529 | 1.75E-07 | AC005592.2 | 6.688578024 | 1.79E-05 |
| AC099552.1 | 3.941466935 | 1.17E-03 | LINC01011 | 6.678114811 | 2.36E-05 |
| AC141928.1 | 3.912394731 | 4.53E-13 | CTC-338M12.4 | 6.659388687 | 3.32E-05 |
| SYNPR-AS1 | 3.893646394 | 2.78E-04 | AC025154.2 | 6.096485131 | 1.29E-06 |
| AP003469.2 | 3.892091677 | 2.35E-07 | LINC02041 | 5.982723576 | 4.77E-10 |
| LINC00443 | 3.886579152 | 1.09E-04 | AL445649.1 | 5.855220194 | 1.68E-08 |
| AL365226.2 | 3.886093518 | 2.50E-03 | LINC02188 | 5.833822912 | 1.71E-08 |
| AC091806.1 | 3.879923432 | 4.33E-07 | AL596223.2 | 5.736779317 | 1.66E-07 |
| LINC01012 | 3.848151716 | 7.25E-08 | GAS5 | 5.684330671 | 1.19E-13 |
| AC007128.1 | 3.845170155 | 1.58E-03 | AL365181.2 | 5.663522551 | 2.20E-17 |
| AP000864.1 | 3.84178448 | 2.78E-06 | AC105219.4 | 5.629924691 | 8.04E-11 |
| LINC01342 | 3.83305105 | 5.36E-06 | LINC01748 | 5.612235056 | 2.05E-05 |
| AC009097.2 | 3.828878541 | 5.23E-06 | MAFG-AS1 | 5.607927575 | 6.64E-27 |
| AL021807.1 | 3.82385984 | 9.98E-07 | HOXC-AS1 | 5.575508029 | 2.33E-04 |
| LINC01116 | 3.819017984 | 4.80E-08 | HAGLR | 5.317978846 | 2.39E-07 |
| AC107959.3 | 3.815028182 | 1.64E-10 | AP002957.1 | 5.176348975 | 2.03E-05 |
| AL590666.2 | 3.813567891 | 2.05E-15 | AC147651.1 | 5.116816612 | 5.30E-10 |
| AC107464.1 | 3.79374394 | 1.90E-04 | LINC02086 | 5.116389627 | 9.16E-06 |
| AC040174.1 | 3.788987574 | 2.63E-04 | AC011352.1 | 5.026824931 | 9.08E-06 |
| LINC02560 | 3.74646001 | 3.82E-05 | AP000696.1 | 5.009301111 | 5.64E-05 |
| HOXC-AS2 | 3.745165302 | 3.86E-05 | AL391056.1 | 4.992889435 | 2.06E-03 |
| HAGLR | 3.743433687 | 2.52E-07 | GACAT2 | 4.916265725 | 2.88E-07 |
| RUNDC3A-AS1 | 3.740211218 | 6.30E-07 | LINC00707 | 4.908030887 | 5.81E-09 |
| AC128688.2 | 3.730462107 | 3.13E-15 | SLCO4A1-AS1 | 4.877761436 | 4.92E-11 |
| SPACA6P-AS | 3.713773564 | 6.67E-07 | AC026368.1 | 4.730654143 | 1.78E-10 |
| AC023421.2 | 3.706671623 | 1.27E-05 | TFAP2A-AS1 | 4.727701449 | 5.87E-12 |
| AC012531.1 | 3.699758738 | 1.28E-03 | UNC5B-AS1 | 4.674855883 | 7.70E-06 |
| AP003721.1 | 3.687474514 | 4.15E-04 | LINC02119 | 4.660237773 | 5.54E-04 |
| AC106772.1 | 3.673823012 | 3.11E-03 | LINC01117 | 4.593822502 | 6.42E-05 |
| AL109976.1 | 3.669425208 | 5.71E-11 | AC007128.1 | 4.575496725 | 1.50E-03 |
| AC084864.1 | 3.660007777 | 2.04E-06 | DLX6-AS1 | 4.565776709 | 6.92E-06 |
| FEZF1-AS1 | 3.651948518 | 3.86E-06 | AL354984.1 | 4.532653167 | 8.79E-05 |
| LINC01659 | 3.644211144 | 9.88E-06 | AC022424.1 | 4.480969069 | 1.72E-05 |
| HOTTIP | 3.644076463 | 1.17E-03 | AC108860.2 | 4.464499714 | 8.10E-18 |
| LINC00323 | 3.637003313 | 5.15E-07 | AC015921.1 | 4.463717676 | 9.69E-06 |
| NRG1-IT1 | 3.636250422 | 7.03E-04 | NKILA | 4.437325648 | 2.95E-14 |
| SLC2A1-AS1 | 3.622443922 | 9.79E-07 | AP001626.1 | 4.403902749 | 2.00E-08 |
| AL355102.4 | 3.60864078 | 2.03E-10 | AL590652.1 | 4.297806576 | 1.05E-08 |
| AC008875.1 | 3.608462925 | 2.26E-07 | ELFN2 | 4.296803762 | 8.69E-05 |
| ROR1-AS1 | 3.607075372 | 1.31E-05 | RNF144A-AS1 | 4.289792189 | 3.51E-04 |
| SPINT1-AS1 | 3.599739257 | 2.59E-31 | NPSR1-AS1 | 4.272262799 | 8.08E-05 |
| AL445070.1 | 3.597792344 | 2.78E-04 | LEF1-AS1 | 4.261817936 | 3.26E-07 |
| AP000911.1 | 3.579937869 | 6.88E-06 | BX470102.1 | 4.222073365 | 1.35E-08 |
| LINC00592 | 3.551016626 | 1.07E-05 | AL121832.1 | 4.146124523 | 2.24E-05 |
| HOXC-AS1 | 3.545177951 | 2.45E-04 | AC016705.2 | 4.138809633 | 9.15E-15 |
| AC110491.1 | 3.542554774 | 4.39E-04 | AL109976.1 | 4.07713912 | 5.71E-11 |
| AC005256.1 | 3.536687597 | 2.05E-04 | LINC02551 | 3.959727011 | 6.24E-05 |
| LINC01658 | 3.528150449 | 3.72E-04 | AC245100.6 | 3.929642638 | 7.14E-05 |
| AL049836.1 | 3.527636496 | 2.10E-04 | AC026992.2 | 3.904621213 | 2.01E-08 |
| AL133215.2 | 3.526895161 | 5.04E-07 | AL080312.2 | 3.883581527 | 5.73E-04 |
| LINC01583 | 3.524101961 | 7.15E-04 | AL357060.1 | 3.862524229 | 6.52E-05 |
| LINC02261 | 3.515036999 | 1.29E-03 | TTC39A-AS1 | 3.747115402 | 1.75E-06 |
| AC074124.1 | 3.513690194 | 2.41E-03 | CASC15 | 3.724119266 | 5.81E-12 |
| LINC02081 | 3.466730468 | 2.48E-04 | LINC00622 | 3.71767475 | 3.62E-07 |
| AC099518.2 | 3.457009661 | 6.13E-06 | AC244034.2 | 3.714977078 | 5.16E-05 |
| AC114488.1 | 3.435836529 | 6.19E-07 | AC009955.2 | 3.705415376 | 2.00E-05 |
| AC005392.3 | 3.428967676 | 1.60E-03 | AP003419.4 | 3.673061753 | 7.06E-11 |
| AC087623.2 | 3.417865929 | 2.50E-07 | AC023302.1 | 3.661409012 | 1.75E-06 |
| AC133794.1 | 3.406727751 | 4.00E-04 | AC007639.1 | 3.621275519 | 5.90E-05 |
| AL022068.1 | 3.397669993 | 1.46E-07 | AC233280.1 | 3.584807865 | 9.48E-05 |
| AL606763.1 | 3.38158832 | 2.16E-06 | AL353801.1 | 3.580833834 | 5.27E-04 |
| LINC00930 | 3.377382499 | 1.18E-04 | Z69733.1 | 3.580763271 | 3.76E-05 |
| AC098864.1 | 3.370824775 | 2.05E-03 | AL121929.2 | 3.579757133 | 1.39E-06 |
| AC010967.1 | 3.366754672 | 1.62E-05 | LINC00992 | 3.54026758 | 7.75E-11 |
| AC105411.1 | 3.353612295 | 2.13E-06 | DPP10-AS1 | 3.480517595 | 3.26E-07 |
| LINC01159 | 3.345790757 | 7.62E-04 | AL121895.2 | 3.457135854 | 8.01E-06 |
| RNF157-AS1 | 3.34408667 | 1.11E-15 | LINC01600 | 3.425371985 | 2.03E-05 |
| AC087477.4 | 3.342833414 | 3.66E-04 | LINC01411 | 3.422826632 | 6.38E-04 |
| LINC02014 | 3.324769178 | 4.91E-04 | SPRY4-AS1 | 3.417871113 | 1.01E-08 |
| AC090541.1 | 3.324706955 | 2.66E-04 | LINC01133 | 3.413994852 | 3.58E-03 |
| LINC01929 | 3.32397257 | 1.86E-04 | AC108134.1 | 3.39132872 | 5.95E-12 |
| AP003419.4 | 3.305755578 | 7.06E-11 | LINC02280 | 3.368343466 | 1.76E-05 |
| AC138305.1 | 3.301257663 | 1.68E-03 | AC078850.1 | 3.328309148 | 5.40E-04 |
| AC124319.1 | 3.282435362 | 1.01E-06 | AC006960.3 | 3.318524435 | 6.07E-05 |
| AL589765.6 | 3.276636485 | 1.60E-04 | AL109615.3 | 3.307750047 | 2.72E-12 |
| AC106900.2 | 3.275812475 | 2.11E-03 | KIAA0087 | 3.302932965 | 6.12E-04 |
| AC092757.2 | 3.268930975 | 9.79E-08 | AC106772.1 | 3.282543367 | 2.95E-03 |
| AC245884.1 | 3.267986878 | 4.76E-05 | AL121992.1 | 3.251776936 | 6.90E-05 |
| DGCR9 | 3.267292841 | 1.85E-11 | AC011773.4 | 3.243766219 | 7.20E-05 |
| AC244517.7 | 3.256321159 | 1.28E-04 | IBA57-AS1 | 3.228792219 | 1.81E-05 |
| MAFG-AS1 | 3.247134818 | 6.64E-27 | LINC01836 | 3.218032974 | 6.64E-11 |
| AC023090.1 | 3.244370946 | 1.09E-03 | AC008760.1 | 3.199130198 | 4.61E-14 |
| STK32A-AS1 | 3.243347365 | 2.08E-03 | LINC02115 | 3.17939829 | 6.15E-04 |
| AC060766.6 | 3.240346218 | 3.32E-05 | AC009121.1 | 3.167140289 | 5.93E-05 |
| AC026992.1 | 3.238551095 | 3.50E-05 | AL121782.1 | 3.161189128 | 5.98E-05 |
| AC005821.1 | 3.215612609 | 9.51E-05 | AC006273.1 | 3.15797157 | 2.19E-05 |
| AL008718.2 | 3.20694107 | 4.46E-06 | PTGES2-AS1 | 3.151847548 | 8.18E-06 |
| AP000695.2 | 3.205203429 | 4.74E-07 | AC004264.1 | 3.151000272 | 7.40E-06 |
| AP001207.3 | 3.204600868 | 7.42E-04 | AC129492.1 | 3.145419579 | 9.19E-06 |
| AC090921.1 | 3.203593449 | 1.84E-04 | AC116337.3 | 3.144317898 | 8.70E-05 |
| AC073323.1 | 3.200848822 | 2.85E-03 | KCNK15-AS1 | 3.097846294 | 5.31E-04 |
| LINC00992 | 3.186240822 | 7.75E-11 | CTB-178M22.2 | 3.092901527 | 1.97E-05 |
| AC012508.2 | 3.164311025 | 7.64E-04 | AC020661.1 | 3.088838168 | 2.94E-07 |
| AL355472.4 | 3.15757453 | 1.68E-05 | TMEM72-AS1 | 3.064654279 | 6.06E-05 |
| LOXL1-AS1 | 3.145499594 | 1.79E-19 | AL021154.1 | 3.060105875 | 7.16E-04 |
| AL670729.1 | 3.134687389 | 3.75E-06 | AC016773.1 | 3.056314187 | 5.93E-10 |
| AC027312.1 | 3.117957981 | 3.43E-04 | AC026250.1 | 3.027304463 | 1.60E-08 |
| HAGLROS | 3.106147492 | 4.79E-05 | AC124798.1 | 3.016756696 | 9.88E-06 |
| ARHGAP31-AS1 | 3.104981351 | 9.88E-06 | AC005618.1 | 3.014154625 | 3.40E-07 |
| LINC00327 | 3.102507715 | 6.74E-06 | AC010247.2 | 2.992287442 | 2.14E-03 |
| TMEM51-AS1 | 3.102130808 | 1.39E-12 | AC022150.4 | 2.982144753 | 8.65E-09 |
| HRAT92 | 3.098210144 | 7.09E-12 | AC145423.2 | 2.97065558 | 8.66E-06 |
| AL731533.2 | 3.096583083 | 2.36E-06 | AL606489.1 | 2.95387054 | 1.76E-08 |
| AL512363.1 | 3.092624774 | 9.75E-04 | LINC01535 | 2.945827141 | 9.44E-05 |
| AC008514.1 | 3.083948615 | 1.54E-07 | AC005083.1 | 2.935081757 | 2.63E-12 |
| LINC00336 | 3.080939601 | 1.34E-03 | AC040970.1 | 2.93041737 | 6.73E-14 |
| CRNDE | 3.07702532 | 1.19E-13 | DDX11-AS1 | 2.893368622 | 5.08E-12 |
| PICART1 | 3.076514145 | 1.28E-06 | AC097059.2 | 2.891139811 | 6.38E-04 |
| AC015712.1 | 3.067798951 | 1.94E-06 | AC245407.1 | 2.884494725 | 1.72E-05 |
| AP003063.1 | 3.067717981 | 7.21E-04 | AC023908.3 | 2.882103862 | 4.80E-10 |
| SPATA3-AS1 | 3.067665677 | 3.70E-05 | AC004130.1 | 2.863277095 | 8.76E-15 |
| AC010247.2 | 3.063646512 | 2.25E-03 | AC022509.3 | 2.856959649 | 9.30E-06 |
| AC009041.2 | 3.060148877 | 6.64E-07 | AL117335.1 | 2.841331822 | 6.84E-11 |
| LINC02389 | 3.05912868 | 1.41E-03 | AC009005.1 | 2.799547837 | 2.14E-08 |
| LINC01978 | 3.054818575 | 1.07E-10 | AF131215.6 | 2.786802735 | 5.78E-11 |
| AC048344.4 | 3.046588906 | 4.37E-05 | LINC01730 | 2.772356948 | 9.69E-06 |
| C15orf56 | 3.01185643 | 4.20E-07 | LINC01186 | 2.77233054 | 1.89E-05 |
| FAM87A | 3.005049008 | 5.04E-04 | AL592211.1 | 2.772127739 | 6.19E-05 |
| LINC01762 | 3.003404567 | 9.98E-06 | VAC14-AS1 | 2.725905735 | 1.41E-06 |
| LINC00628 | 3.00170306 | 2.58E-05 | AC009549.1 | 2.722308149 | 6.65E-05 |
| AP000757.1 | 3.001247313 | 2.12E-07 | AP001922.5 | 2.720159327 | 1.86E-05 |
| LINC01913 | 3.001220667 | 9.23E-04 | AC239868.2 | 2.716317732 | 1.53E-17 |
| AC018553.1 | 3.001143396 | 2.11E-04 | AC009414.2 | 2.689070637 | 1.63E-08 |
| AL160286.2 | 2.999797342 | 4.69E-04 | AC023449.2 | 2.683924242 | 4.03E-04 |
| Z97200.1 | 2.999512728 | 8.37E-04 | AC087742.2 | 2.66123502 | 7.97E-05 |
| LINC02365 | 2.995546946 | 4.35E-04 | AL137784.2 | 2.617792288 | 3.58E-07 |
| AC108463.3 | 2.991695691 | 1.05E-04 | VIM-AS1 | 2.595285621 | 6.30E-10 |
| LINC00942 | 2.991057369 | 2.62E-04 | LINC00887 | 2.59242651 | 7.73E-05 |
| AC020922.3 | 2.990578171 | 3.65E-05 | AP003390.1 | 2.585301649 | 1.07E-04 |
| AL355388.2 | 2.987071896 | 1.44E-10 | AC006538.1 | 2.581936706 | 2.26E-08 |
| AC027228.2 | 2.96713323 | 7.01E-07 | AC016866.1 | 2.580448978 | 1.29E-06 |
| AC124798.1 | 2.956383928 | 1.04E-05 | AL365203.3 | 2.573035471 | 4.50E-10 |
| AP003354.2 | 2.95530892 | 2.15E-06 | AL022322.1 | 2.572287126 | 8.02E-11 |
| AC020659.1 | 2.951603334 | 7.43E-08 | AL133410.1 | 2.567744024 | 9.25E-09 |
| AL022344.2 | 2.946744737 | 9.39E-04 | LINC01948 | 2.563330939 | 1.88E-06 |
| DNAH17-AS1 | 2.934732222 | 2.25E-04 | AC091544.5 | 2.559947373 | 6.06E-05 |
| AC017002.3 | 2.929549596 | 3.00E-05 | AC102953.2 | 2.507311932 | 6.67E-18 |
| AC005696.4 | 2.925636223 | 9.54E-04 | AC105219.1 | 2.491793383 | 1.62E-08 |
| LINC01711 | 2.923627595 | 2.77E-04 | DDN-AS1 | 2.478240057 | 9.50E-06 |
| AP000424.2 | 2.912424865 | 1.18E-05 | CYTOR | 2.467176958 | 4.64E-17 |
| AC017104.1 | 2.912193041 | 2.63E-11 | AL161772.1 | 2.451872747 | 7.49E-05 |
| LINC02097 | 2.898815281 | 6.17E-06 | AC009542.1 | 2.441010996 | 5.82E-04 |
| LINC01836 | 2.896229677 | 6.64E-11 | AL008582.1 | 2.418977177 | 7.05E-06 |
| AL118505.1 | 2.892999896 | 4.87E-05 | AC092117.1 | 2.413550954 | 3.98E-09 |
| Z92544.2 | 2.892900167 | 5.24E-15 | AC027117.1 | 2.405755456 | 7.86E-05 |
| AL353593.3 | 2.891987632 | 2.21E-04 | AL137145.1 | 2.334168428 | 1.46E-06 |
| PP14571 | 2.8917524 | 9.80E-05 | AL731569.1 | 2.321591725 | 3.93E-10 |
| AL359878.1 | 2.886015823 | 1.37E-09 | LINC01963 | 2.272471838 | 7.43E-14 |
| LINC02298 | 2.883439053 | 7.03E-16 | AP001029.2 | 2.267502475 | 5.03E-05 |
| AC022509.1 | 2.881912781 | 2.77E-03 | TM4SF1-AS1 | 2.263396269 | 9.17E-05 |
| RMST | 2.880669381 | 1.95E-03 | KRT7-AS | 2.254704075 | 8.81E-06 |
| DGCR10 | 2.877479634 | 1.40E-05 | ZMIZ1-AS1 | 2.253607661 | 3.52E-07 |
| C1QTNF1-AS1 | 2.875656128 | 1.30E-04 | FAM66B | 2.238823284 | 5.18E-05 |
| AL031663.3 | 2.874474086 | 1.93E-03 | PTPRG-AS1 | 2.219348462 | 2.22E-05 |
| AC023301.1 | 2.868096971 | 3.56E-03 | AC126118.1 | 2.21793978 | 5.92E-05 |
| AL359881.1 | 2.866756721 | 1.09E-04 | MIR181A2HG | 2.216486463 | 2.57E-07 |
| AL512328.1 | 2.862306376 | 4.75E-04 | AC093690.1 | 2.176887 | 5.75E-05 |
| AC036108.4 | 2.851183478 | 1.69E-04 | AC006449.2 | 2.173123105 | 5.86E-05 |
| AC023449.2 | 2.850982236 | 4.24E-04 | FRMD6-AS1 | 2.172914585 | 6.56E-05 |
| AC091057.3 | 2.850598537 | 1.65E-05 | U91328.4 | 2.154880886 | 6.17E-04 |
| AL365356.5 | 2.839656254 | 2.69E-03 | AC011921.1 | 2.144097285 | 6.86E-05 |
| AC010524.1 | 2.837627519 | 1.43E-04 | AC002398.1 | 2.117047293 | 5.10E-09 |
| AC004540.1 | 2.832951741 | 3.63E-10 | AC084033.3 | 2.109859911 | 8.44E-06 |
| LINC01123 | 2.83057322 | 1.05E-12 | AC010864.1 | 2.097989726 | 1.38E-06 |
| AC244517.1 | 2.827334344 | 2.17E-04 | AC008764.8 | 2.09304676 | 8.41E-11 |
| AC133644.2 | 2.825262058 | 1.74E-04 | AL353801.3 | 2.091601889 | 7.29E-05 |
| LINC02038 | 2.799266993 | 4.85E-06 | AC110048.2 | 2.090207496 | 8.95E-05 |
| CDKN2B-AS1 | 2.78523856 | 7.44E-08 | AC010201.1 | 2.07175581 | 2.30E-05 |
| TGFB2-AS1 | 2.774740472 | 2.67E-05 | AL024498.1 | 2.064843081 | 9.23E-06 |
| AC006557.1 | 2.767794806 | 1.02E-04 | AC234582.1 | 2.042994359 | 1.34E-08 |
| LINC00622 | 2.76412219 | 3.81E-07 | AC092171.2 | 2.039724703 | 2.76E-14 |
| AL355472.3 | 2.762686455 | 1.04E-03 | AL035563.1 | 2.018210232 | 4.86E-09 |
| AC120498.3 | 2.759699981 | 1.25E-03 | AL353593.1 | 2.012931778 | 7.12E-05 |
| AC005393.1 | 2.756556652 | 6.98E-07 | AL162591.1 | -2.00641392 | 1.10E-07 |
| AC016773.1 | 2.750682768 | 5.93E-10 | CDC37L1-AS1 | -2.006751355 | 4.68E-15 |
| AC079779.2 | 2.750345712 | 2.24E-04 | AC036214.2 | -2.01453088 | 2.60E-15 |
| AC025580.1 | 2.734751909 | 3.77E-04 | AC133552.6 | -2.016026528 | 1.79E-18 |
| CYP4F26P | 2.733532521 | 1.48E-04 | LINC02447 | -2.018018732 | 1.11E-12 |
| AC107464.3 | 2.733060188 | 4.18E-05 | AC124242.1 | -2.018548529 | 5.22E-17 |
| NEBL-AS1 | 2.733011366 | 1.37E-04 | AC025857.2 | -2.020775811 | 4.63E-10 |
| AC092683.1 | 2.729872812 | 5.67E-08 | LINC01124 | -2.02779735 | 2.73E-06 |
| MELTF-AS1 | 2.727435227 | 8.12E-20 | RAB11B-AS1 | -2.032453523 | 8.23E-13 |
| LINC01336 | 2.724284085 | 5.99E-07 | AL023581.2 | -2.03967676 | 2.75E-26 |
| LINC02331 | 2.717503932 | 4.82E-06 | AL449106.1 | -2.061336036 | 4.25E-12 |
| AC114811.2 | 2.715230501 | 1.39E-03 | AL137003.2 | -2.065276605 | 2.14E-14 |
| AL390719.2 | 2.701315823 | 2.57E-11 | CALML3-AS1 | -2.113902908 | 5.69E-04 |
| AP000553.1 | 2.69910708 | 3.06E-04 | AC129507.2 | -2.119037126 | 2.42E-08 |
| FLJ12825 | 2.689662849 | 5.41E-06 | ARHGEF26-AS1 | -2.144301522 | 6.89E-06 |
| AC073592.1 | 2.686365333 | 4.21E-04 | AC005920.1 | -2.154593436 | 1.86E-05 |
| PCAT14 | 2.684353029 | 5.16E-04 | CATIP-AS1 | -2.161477336 | 1.43E-06 |
| AL139241.1 | 2.678216669 | 1.13E-04 | LINC02012 | -2.165079423 | 6.09E-04 |
| AP003390.1 | 2.672621827 | 1.13E-04 | AP001267.3 | -2.166593369 | 2.06E-08 |
| AL513217.1 | 2.6720654 | 9.51E-04 | AC063943.3 | -2.195549029 | 2.23E-08 |
| AC243772.2 | 2.666959899 | 4.77E-05 | SMIM2-AS1 | -2.196322966 | 7.50E-05 |
| AL645608.8 | 2.666214212 | 2.84E-09 | ENTPD3-AS1 | -2.212450884 | 8.88E-17 |
| AC139149.1 | 2.661340152 | 1.24E-07 | AC010273.2 | -2.212453206 | 5.37E-06 |
| SZT2-AS1 | 2.652719363 | 1.60E-03 | LINC02004 | -2.213849762 | 3.15E-07 |
| AL355997.1 | 2.637345433 | 1.32E-04 | Z98884.1 | -2.216466544 | 2.32E-05 |
| AL137847.1 | 2.634145696 | 3.79E-04 | AP001528.1 | -2.22230591 | 1.96E-07 |
| AC026356.2 | 2.613476199 | 4.50E-07 | SUCLG2-AS1 | -2.229037049 | 3.84E-09 |
| CYP4A22-AS1 | 2.612541864 | 3.66E-06 | AC003075.1 | -2.229912934 | 3.69E-09 |
| AC108676.1 | 2.610722352 | 4.76E-07 | AC018926.2 | -2.24135849 | 4.41E-08 |
| STAU2-AS1 | 2.60503082 | 1.09E-03 | LINC01549 | -2.244880888 | 4.78E-04 |
| AL137145.2 | 2.598849028 | 1.08E-05 | AC009831.1 | -2.248395511 | 2.01E-15 |
| AC139887.4 | 2.598651653 | 2.95E-05 | AC012640.4 | -2.263546101 | 1.64E-08 |
| AC011944.1 | 2.598221327 | 1.58E-03 | AC108673.2 | -2.268763661 | 1.70E-10 |
| AC127496.1 | 2.595594591 | 1.06E-03 | FGF14-AS2 | -2.290329169 | 3.17E-17 |
| AC023908.3 | 2.593893476 | 4.80E-10 | AC079949.1 | -2.298327161 | 5.38E-05 |
| STAM-AS1 | 2.576868461 | 4.82E-08 | AL157756.1 | -2.321247483 | 3.08E-12 |
| AC245041.1 | 2.576024237 | 3.82E-04 | AP003119.2 | -2.366316776 | 8.78E-05 |
| AC105446.1 | 2.574450315 | 2.53E-03 | AL450384.1 | -2.370291294 | 3.43E-06 |
| AC116407.2 | 2.573107651 | 2.53E-07 | AP001528.2 | -2.371637969 | 1.80E-09 |
| AC092611.2 | 2.569954232 | 1.54E-04 | LINC00864 | -2.381333408 | 1.49E-03 |
| GAPLINC | 2.562234409 | 2.16E-03 | AL596325.2 | -2.382094 | 3.05E-05 |
| AL117335.1 | 2.55719864 | 6.84E-11 | AC079949.2 | -2.386199759 | 9.28E-06 |
| AC008105.1 | 2.557128988 | 1.44E-10 | AF111167.2 | -2.387452966 | 7.94E-11 |
| AL391845.2 | 2.554414575 | 3.43E-05 | LINC01564 | -2.39719885 | 1.39E-04 |
| AP000866.5 | 2.5537392 | 1.86E-04 | CPB2-AS1 | -2.4153704 | 1.66E-08 |
| AC009148.1 | 2.551450836 | 1.58E-07 | AC024361.2 | -2.416957374 | 4.32E-10 |
| IL12A-AS1 | 2.548471123 | 2.49E-03 | PRKAG2-AS1 | -2.432821732 | 3.49E-12 |
| AC010615.2 | 2.547500092 | 9.92E-04 | AP003555.3 | -2.436037258 | 5.24E-05 |
| ATP2A1-AS1 | 2.539237574 | 3.80E-05 | AC138123.2 | -2.463947998 | 5.49E-05 |
| AC027288.3 | 2.538451028 | 3.24E-03 | AC016924.1 | -2.477134059 | 2.13E-05 |
| C10orf91 | 2.538431096 | 1.36E-04 | AC114947.2 | -2.510913016 | 6.29E-11 |
| C10orf55 | 2.53748466 | 3.86E-03 | AL109741.1 | -2.519841278 | 1.64E-08 |
| AC012358.1 | 2.529900724 | 3.37E-03 | LINC01277 | -2.541721397 | 6.66E-14 |
| SERTAD4-AS1 | 2.526236237 | 1.30E-03 | RRS1-AS1 | -2.611169045 | 2.66E-07 |
| AC017083.2 | 2.524871742 | 3.01E-05 | AL357055.3 | -2.666883994 | 3.00E-20 |
| CAPN10-AS1 | 2.519471469 | 1.91E-15 | AC026202.2 | -2.674027639 | 2.67E-18 |
| AC092757.3 | 2.512054214 | 2.57E-06 | AC083900.1 | -2.684083162 | 8.95E-11 |
| AP000525.1 | 2.511200396 | 1.58E-03 | USP30-AS1 | -2.684974343 | 2.58E-07 |
| AC079315.1 | 2.50932189 | 2.67E-06 | LINC01725 | -2.686694855 | 2.52E-09 |
| AF131215.6 | 2.508122462 | 5.78E-11 | AC104785.1 | -2.694687885 | 6.86E-13 |
| LINC00483 | 2.507994019 | 9.91E-04 | AC073842.1 | -2.699292009 | 1.33E-04 |
| AC011330.2 | 2.505199186 | 4.54E-05 | AL139383.1 | -2.708319497 | 4.61E-08 |
| LINC01671 | 2.503809576 | 1.42E-04 | C2-AS1 | -2.71393564 | 2.46E-06 |
| AC004466.3 | 2.499239895 | 1.64E-04 | LIVAR | -2.724793611 | 2.70E-05 |
| AP006284.1 | 2.497060511 | 3.14E-11 | AL031055.1 | -2.726094596 | 1.37E-16 |
| LINC00865 | 2.496705562 | 5.17E-04 | LINC00337 | -2.737325751 | 3.82E-12 |
| WFDC21P | 2.493734796 | 1.04E-03 | AC022784.3 | -2.740161035 | 1.55E-10 |
| AC026356.1 | 2.492259098 | 2.10E-09 | AC114810.1 | -2.745539783 | 4.52E-17 |
| KRTAP5-AS1 | 2.49019142 | 4.81E-06 | AC036176.1 | -2.751475865 | 9.31E-25 |
| AC245100.7 | 2.489764648 | 8.94E-04 | AC239800.2 | -2.751595259 | 1.04E-03 |
| AC127024.6 | 2.4896222 | 1.45E-04 | AC027117.2 | -2.754165346 | 7.82E-05 |
| AL591845.1 | 2.489178229 | 4.42E-08 | AC015908.2 | -2.754596438 | 7.76E-05 |
| AL035448.1 | 2.489147562 | 3.80E-03 | AC104411.1 | -2.788204528 | 1.42E-08 |
| LINC01106 | 2.487325045 | 1.73E-10 | AC002401.3 | -2.805330311 | 2.55E-08 |
| AC026740.1 | 2.48654827 | 3.37E-06 | AC116036.2 | -2.830073572 | 4.78E-10 |
| AL122125.1 | 2.477087457 | 1.25E-04 | AC104170.1 | -2.852967925 | 3.99E-12 |
| SLC12A9-AS1 | 2.475722481 | 6.94E-07 | AC002401.2 | -2.877095692 | 1.72E-05 |
| AC233280.1 | 2.472281286 | 9.98E-05 | MAGI2-AS3 | -2.884028215 | 5.06E-14 |
| AC099850.3 | 2.467146566 | 6.95E-08 | PAQR9-AS1 | -2.925699748 | 9.15E-05 |
| NFIA-AS2 | 2.462215115 | 7.18E-04 | AL451060.1 | -3.043146504 | 1.87E-06 |
| AL590235.1 | 2.460005499 | 9.20E-08 | NDUFA6-AS1 | -3.078590961 | 1.65E-14 |
| AC132872.2 | 2.456908273 | 1.04E-06 | AC011365.1 | -3.145530615 | 8.27E-06 |
| HEXA-AS1 | 2.455126018 | 3.80E-05 | LINC01816 | -3.219013765 | 1.48E-14 |
| AC099521.2 | 2.452446171 | 2.08E-04 | AL034374.2 | -3.222437889 | 4.74E-09 |
| PAX8-AS1 | 2.452243491 | 3.69E-05 | AC005225.2 | -3.248487424 | 5.30E-09 |
| AC008764.8 | 2.448864709 | 8.41E-11 | AC015917.2 | -3.28804924 | 6.50E-12 |
| AL359881.2 | 2.447589687 | 1.19E-03 | MIR99AHG | -3.37086488 | 5.95E-18 |
| AC010201.2 | 2.447169057 | 1.43E-09 | LINC02073 | -3.471476902 | 4.88E-09 |
| AC093151.2 | 2.445561588 | 2.89E-04 | LINC02388 | -3.479013239 | 6.92E-09 |
| AC090260.1 | 2.44009365 | 3.26E-04 | AC099684.2 | -3.503121195 | 5.29E-10 |
| AL121772.1 | 2.439890992 | 4.25E-07 | AC016999.1 | -3.530357671 | 2.75E-07 |
| AC092171.4 | 2.437141793 | 1.26E-09 | AC012313.9 | -3.606731655 | 3.01E-27 |
| AL591895.1 | 2.435893032 | 3.21E-10 | AP001065.2 | -3.737698911 | 7.25E-18 |
| LINC01311 | 2.434488635 | 2.96E-08 | AP003119.3 | -3.774741238 | 1.93E-14 |
| AC073842.2 | 2.432850668 | 6.84E-06 | AL137798.1 | -3.778790831 | 3.34E-03 |
| GS1-24F4.2 | 2.428314785 | 3.45E-04 | AC009093.5 | -3.856582493 | 5.55E-14 |
| AP001271.1 | 2.428032038 | 6.48E-04 | AC012065.3 | -3.87845611 | 5.83E-18 |
| AC007750.1 | 2.426139929 | 4.20E-04 | AC254562.2 | -3.937975236 | 7.85E-15 |
| AC073283.1 | 2.423630366 | 2.68E-03 | TUSC8 | -3.994841443 | 9.60E-06 |
| FIRRE | 2.422292355 | 5.15E-04 | LINC02484 | -4.172384852 | 1.90E-06 |
| AC027702.1 | 2.422283044 | 5.03E-07 | LINC00907 | -4.212538177 | 7.12E-18 |
| AP000424.1 | 2.416972097 | 3.47E-05 | AC008592.4 | -4.216970162 | 3.38E-18 |
| AL353751.1 | 2.415566475 | 3.36E-03 | AC137056.1 | -4.230583358 | 5.28E-10 |
| AF131215.5 | 2.411928003 | 1.87E-09 | AC087045.2 | -4.243930871 | 2.32E-17 |
| FAM66C | 2.408846179 | 3.63E-13 | AL121992.3 | -4.260184877 | 3.87E-14 |
| AC087645.2 | 2.408629928 | 1.09E-04 | AC090877.2 | -4.333801968 | 7.80E-15 |
| GAS5 | 2.406641292 | 1.25E-13 | KLHL6-AS1 | -4.384702905 | 3.94E-12 |
| AC092667.1 | 2.405320209 | 5.08E-04 | AC083841.1 | -4.440189491 | 4.92E-11 |
| AC134682.1 | 2.403492582 | 1.91E-04 | AL136040.1 | -4.486720322 | 6.18E-10 |
| AC109361.2 | 2.402287437 | 1.01E-03 | LINC02266 | -4.515522123 | 3.10E-07 |
| BX255925.1 | 2.400584162 | 2.47E-07 | AC009166.1 | -4.534146061 | 5.27E-12 |
| AL163953.1 | 2.397721146 | 9.65E-04 | AC068631.1 | -4.619252077 | 7.56E-28 |
| AC139100.2 | 2.397664331 | 1.09E-05 | AL023755.1 | -4.644512089 | 5.87E-14 |
| AC127164.1 | 2.397036473 | 4.10E-05 | AC092384.2 | -4.652814443 | 8.90E-15 |
| AC019205.2 | 2.395898195 | 1.07E-10 | AC087392.1 | -4.743397477 | 7.41E-14 |
| AL357079.1 | 2.395519658 | 1.27E-06 | AL772337.1 | -4.953089171 | 1.30E-08 |
| AP001178.3 | 2.394633022 | 2.29E-04 | LINC02506 | -4.968347899 | 2.31E-05 |
| AL445490.1 | 2.388427196 | 2.61E-03 | AL356056.3 | -4.978914211 | 4.13E-26 |
| AP000808.2 | 2.382382517 | 1.18E-03 | AC079061.1 | -5.213532395 | 1.61E-14 |
| AC068189.1 | 2.379404566 | 2.03E-03 | AP001043.1 | -5.25019403 | 3.92E-27 |
| U47924.3 | 2.375057417 | 1.40E-05 | AC005035.1 | -5.271992132 | 6.19E-14 |
| AC098614.4 | 2.371263933 | 1.79E-04 | AL354872.2 | -5.321089683 | 9.50E-26 |
| AC025171.4 | 2.370736229 | 1.98E-06 | AL133419.1 | -5.441184718 | 3.69E-07 |
| AC022784.1 | 2.365147291 | 1.84E-03 | LINC02428 | -5.727581699 | 8.27E-09 |
| AC008741.2 | 2.362690179 | 2.55E-15 | AC006205.2 | -5.785150185 | 6.37E-14 |
| AC004923.4 | 2.360778423 | 5.97E-06 | LINC02289 | -5.786624495 | 8.27E-13 |
| AL445472.1 | 2.360744796 | 8.43E-08 | AL390778.2 | -5.788151993 | 2.25E-08 |
| AC003070.1 | 2.360281762 | 1.09E-06 | LINC02037 | -5.904661837 | 1.40E-08 |
| AL158151.4 | 2.358859739 | 1.83E-03 | LINC02499 | -5.933204092 | 2.69E-08 |
| LINC00315 | 2.358280859 | 2.40E-04 | AP003716.1 | -6.081755582 | 1.13E-26 |
| LINC01133 | 2.354479208 | 3.77E-03 | AC022784.6 | -6.336447973 | 2.63E-17 |
| LINC01589 | 2.351494482 | 1.11E-04 | AL121827.1 | -6.581307412 | 6.13E-11 |
| AL033384.2 | 2.351444088 | 1.34E-04 | RP5-834N19.1 | -6.677699424 | 1.14E-05 |
| AL607028.1 | 2.348072844 | 2.10E-04 | RP11-700H6.4 | -6.683216409 | 6.28E-04 |
| AP002360.4 | 2.347431722 | 2.48E-06 | LINC01430 | -6.735249354 | 2.31E-06 |
| Z97653.1 | 2.346968204 | 1.04E-03 | RP11-19D2.1 | -6.760990485 | 2.97E-04 |
| AC110597.1 | 2.346524198 | 3.27E-06 | AP006216.5 | -6.785868475 | 7.13E-06 |
| LINC01914 | 2.345132317 | 3.40E-03 | AP001065.15 | -6.790531297 | 2.81E-05 |
| C9orf163 | 2.344735254 | 1.52E-05 | AC008549.1 | -6.854094984 | 8.68E-09 |
| MIR4435-2HG | 2.343919796 | 1.81E-24 | RP11-15I11.3 | -6.860907811 | 2.17E-04 |
| AC016722.3 | 2.341348911 | 1.53E-05 | RP11-1136G11.8 | -6.872011931 | 3.71E-06 |
| VIM-AS1 | 2.335757059 | 6.30E-10 | AC004160.4 | -6.957383976 | 5.03E-07 |
| AC008750.3 | 2.333802214 | 1.18E-03 | CTD-2350C19.2 | -6.981253715 | 1.46E-04 |
| AL731567.1 | 2.327217516 | 4.24E-05 | RP11-589M4.1 | -7.134920891 | 3.06E-05 |
| AL022476.1 | 2.326346827 | 2.20E-06 | RP11-279F6.1 | -7.149605111 | 3.78E-04 |
| AC096637.2 | 2.325713416 | 1.89E-03 | LINC01558 | -7.168528253 | 5.52E-04 |
| AP001628.1 | 2.320464277 | 3.42E-04 | RP11-799B12.2 | -7.179936772 | 1.51E-06 |
| GATA2-AS1 | 2.320426808 | 1.34E-05 | RP11-390F4.3 | -7.218498113 | 8.97E-05 |
| AL365203.3 | 2.315731924 | 4.50E-10 | RP11-250B2.3 | -7.279257127 | 2.97E-05 |
| LINC02345 | 2.314355715 | 2.75E-03 | LINC01093 | -7.284513492 | 5.53E-09 |
| LINC01943 | 2.311502171 | 6.19E-07 | GS1-21A4.1 | -7.304438704 | 3.90E-07 |
| LINC01152 | 2.309701358 | 3.74E-05 | RP11-118B18.2 | -7.321777295 | 1.39E-05 |
| AC011462.4 | 2.304370139 | 1.02E-09 | RP11-344P13.4 | -7.345643281 | 9.60E-06 |
| LINC02519 | 2.300433059 | 4.12E-04 | HPN-AS1 | -7.393295716 | 7.19E-06 |
| AL445228.2 | 2.296788306 | 2.48E-04 | RP11-96D1.6 | -7.41247536 | 2.72E-05 |
| AL021392.1 | 2.286297278 | 5.30E-08 | AC005339.2 | -7.431980652 | 1.45E-04 |
| AC009269.5 | 2.283728741 | 2.89E-03 | RP5-1103B4.3 | -7.457237692 | 4.65E-07 |
| AL137060.1 | 2.281640108 | 8.10E-07 | AC104809.2 | -7.471709859 | 3.09E-06 |
| AL691482.3 | 2.27842509 | 1.05E-05 | RP11-172E9.2 | -7.530224457 | 1.03E-05 |
| AC116351.1 | 2.277036007 | 1.34E-05 | HAO2-IT1 | -7.542552944 | 1.89E-07 |
| AL121895.1 | 2.276919158 | 9.03E-04 | RP11-326C3.2 | -7.598735156 | 1.02E-04 |
| FLJ31356 | 2.270941101 | 1.41E-04 | CTD-3080P12.3 | -7.606137454 | 3.52E-07 |
| AL021154.1 | 2.248348879 | 7.54E-04 | RP11-513G11.3 | -7.62954933 | 3.76E-06 |
| AC093297.2 | 2.237326513 | 5.13E-08 | RP11-74C13.4 | -7.659883635 | 5.45E-04 |
| AL117379.1 | 2.235745121 | 1.12E-11 | RP11-261N11.8 | -7.679556393 | 5.75E-07 |
| DLGAP1-AS3 | 2.233147275 | 3.46E-03 | LINC00313 | -7.804042566 | 1.93E-04 |
| AC011481.1 | 2.231896719 | 1.93E-06 | RP11-469A15.2 | -7.849420641 | 4.73E-05 |
| AC015987.1 | 2.226732245 | 1.30E-03 | RP11-456H18.2 | -7.861051578 | 1.98E-05 |
| HIF1A-AS2 | 2.2178784 | 2.02E-03 | AC007319.1 | -7.880220032 | 1.26E-04 |
| AP001347.1 | 2.214545526 | 1.96E-04 | RP11-383B4.4 | -7.941552354 | 1.37E-07 |
| AC002550.2 | 2.21389446 | 2.37E-07 | RP4-669P10.16 | -7.99724799 | 1.28E-05 |
| C2orf48 | 2.200034994 | 3.08E-04 | RP11-573D15.8 | -8.025751507 | 2.18E-07 |
| PRR7-AS1 | 2.194724241 | 5.42E-07 | AC004019.13 | -8.043296119 | 5.09E-08 |
| AC092111.1 | 2.19238798 | 7.02E-07 | AC008592.8 | -8.08815162 | 4.24E-07 |
| AC120498.10 | 2.187930262 | 1.50E-03 | RP11-548B3.3 | -8.129062701 | 3.16E-05 |
| NEXN-AS1 | 2.183344922 | 2.42E-04 | RP4-680D5.8 | -8.171769353 | 1.24E-05 |
| AC090409.1 | 2.181891558 | 4.02E-04 | LINC01727 | -8.249218398 | 5.00E-11 |
| AC139749.1 | 2.181508407 | 4.90E-05 | RP11-191G24.1 | -8.283687689 | 1.24E-05 |
| CCND2-AS1 | 2.174806436 | 2.88E-03 | FAM83A-AS1 | -8.440027065 | 3.27E-06 |
| AC007497.1 | 2.173975051 | 2.14E-03 | FTCD-AS1 | -8.454268353 | 6.42E-07 |
| LINC00239 | 2.171474736 | 5.61E-06 | RP11-548L20.1 | -8.485758033 | 1.43E-07 |
| LINC00944 | 2.171229089 | 1.85E-03 | NADK2-AS1 | -8.594249503 | 1.18E-06 |
| SH3PXD2A-AS1 | 2.170135221 | 2.53E-03 | RP11-706C16.7 | -8.600151162 | 2.86E-05 |
| Z69733.1 | 2.169491911 | 3.96E-05 | RP11-181C3.1 | -8.672653065 | 9.98E-06 |
| BAALC-AS1 | 2.167899416 | 3.79E-08 | RP11-21L23.2 | -8.693473102 | 2.80E-07 |
| AC009275.1 | 2.163830163 | 2.71E-05 | RP11-192H23.5 | -8.695604723 | 5.65E-04 |
| AC104794.4 | 2.163608364 | 2.70E-03 | RP11-6N17.6 | -8.855167765 | 9.44E-06 |
| CR559946.2 | 2.162911239 | 1.06E-04 | C4B-AS1 | -8.860915775 | 1.18E-04 |
| AC090515.2 | 2.160102262 | 2.74E-08 | RP11-669E14.4 | -8.862596634 | 6.14E-06 |
| LINC02421 | 2.157986842 | 1.75E-03 | TPRG1-AS1 | -8.933465275 | 3.17E-06 |
| AC004988.1 | 2.155106062 | 1.42E-03 | RP11-38L15.8 | -9.195608175 | 2.57E-06 |
| AL445183.2 | 2.153163025 | 2.02E-03 | RP5-888M10.2 | -9.24646838 | 1.80E-08 |
| AC120036.5 | 2.152038914 | 3.30E-04 | LINC00238 | -9.282854486 | 2.96E-09 |
| LINC01572 | 2.148904632 | 2.85E-05 | RP11-115C10.1 | -9.418393766 | 6.82E-05 |
| AL121772.3 | 2.147617624 | 3.52E-08 | MGC32805 | -9.452709575 | 3.16E-07 |
| AL121760.1 | 2.143926464 | 9.90E-04 | RP11-499P20.2 | -9.482831149 | 2.95E-07 |
| AC245041.2 | 2.14102723 | 1.03E-04 | CTD-2194A8.2 | -9.548590355 | 9.15E-08 |
| AC078778.1 | 2.131961973 | 4.36E-07 | AC016768.1 | -9.56952613 | 4.02E-09 |
| FBXL19-AS1 | 2.126161851 | 1.83E-11 | RP11-6B4.1 | -9.620892957 | 4.88E-08 |
| AC023794.5 | 2.109931265 | 2.89E-03 | CTD-2619J13.8 | -9.662697543 | 1.12E-07 |
| AC114488.2 | 2.107211347 | 9.96E-05 | LINC01314 | -9.680875796 | 5.33E-09 |
| AL365203.1 | 2.105143391 | 1.09E-03 | RP11-153K11.3 | -9.73065493 | 6.38E-08 |
| AL118511.1 | 2.102807052 | 1.15E-04 | AP006285.6 | -9.850791442 | 7.72E-10 |
| AC020907.4 | 2.102547108 | 2.99E-06 | RP11-484N16.1 | -10.13129022 | 1.67E-07 |
| AC138150.2 | 2.102210168 | 2.49E-07 | RP11-612B6.2 | -10.14811161 | 2.40E-05 |
| AC004520.1 | 2.100892453 | 6.23E-07 | RP11-115J16.1 | -10.27791714 | 3.27E-09 |
| LINC00565 | 2.099696861 | 7.99E-04 | CTC-297N7.9 | -10.29778375 | 6.45E-08 |
| MEG3 | 2.097985452 | 2.18E-03 | CTC-537E7.3 | -10.29834151 | 7.69E-10 |
| AGAP2-AS1 | 2.097659447 | 2.27E-10 | AC004862.6 | -10.30557485 | 4.36E-06 |
| LINC01136 | 2.095738549 | 1.69E-03 | AC004538.3 | -10.34508375 | 8.74E-09 |
| AL133367.1 | 2.094208745 | 3.62E-09 | RP13-650J16.1 | -10.35418359 | 6.64E-08 |
| AC005062.1 | 2.092495755 | 2.66E-04 | TMEM220-AS1 | -10.50646134 | 4.86E-08 |
| LINC00853 | 2.092185657 | 7.68E-07 | LAMA5-AS1 | -10.5149019 | 2.23E-05 |
| AL731569.1 | 2.089432553 | 3.93E-10 | AL161668.5 | -10.99894276 | 2.14E-07 |
| AL449403.2 | 2.088139679 | 4.43E-04 | RP11-238F2.1 | -11.07833274 | 5.66E-09 |
| AC124312.3 | 2.08607582 | 3.39E-04 | RP11-256L6.3 | -11.11925418 | 1.85E-09 |
| AL121839.2 | 2.082397371 | 9.60E-11 | U91324.1 | -11.19980062 | 5.73E-11 |
| AC245595.1 | 2.08140128 | 1.21E-04 | RP11-108O10.2 | -11.21575303 | 8.79E-08 |
| AL512413.1 | 2.079699885 | 3.55E-04 | CTD-2540B15.11 | -11.24839083 | 6.58E-08 |
| AL022322.1 | 2.079215345 | 8.02E-11 | AC104809.4 | -11.33056855 | 1.13E-09 |
| FZD10-AS1 | 2.078517819 | 1.66E-05 | C4A-AS1 | -11.34818085 | 4.71E-08 |
| AL359091.4 | 2.076050877 | 8.10E-04 | AC068535.3 | -11.53819463 | 8.13E-10 |
| LINC00304 | 2.067696448 | 1.42E-03 | AP006285.7 | -11.55965394 | 1.07E-11 |
| AL391056.1 | 2.064061679 | 2.17E-03 | RP11-290F5.1 | -11.58048267 | 1.14E-09 |
| SAPCD1-AS1 | 2.062795551 | 2.82E-05 | HS1BP3-IT1 | -11.61633345 | 3.70E-08 |
| TESC-AS1 | 2.058870905 | 1.92E-03 | RP11-168L7.1 | -11.68149677 | 6.08E-12 |
| AC037487.1 | 2.046498223 | 4.74E-04 | LINC00261 | -11.76301315 | 9.20E-07 |
| AL031717.1 | 2.041747824 | 1.70E-04 | AP001048.4 | -11.7793249 | 5.33E-07 |
| FAM66D | 2.040111595 | 1.00E-03 | RP11-109D20.2 | -11.83456722 | 3.62E-05 |
| AC006042.1 | 2.032962769 | 9.88E-08 | FLJ22763 | -12.29580097 | 6.15E-10 |
| UBE2Q1-AS1 | 2.023829843 | 2.49E-04 | RP11-434D9.1 | -12.32749214 | 1.29E-09 |
| FAM201A | 2.020146151 | 4.06E-08 | FAM99B | -12.40106836 | 4.48E-14 |
| AC011294.1 | 2.017122916 | 3.42E-03 | RP11-484D2.4 | -12.41462728 | 6.71E-08 |
| AL008721.2 | 2.010870721 | 6.21E-08 | RP11-130L8.2 | -12.49162761 | 4.05E-08 |
| LINC00704 | 2.003410284 | 8.33E-04 | LINC01370 | -12.63596716 | 1.02E-10 |
| DRAIC | -2.047639642 | 1.56E-04 | RP11-798K3.2 | -12.63990659 | 1.81E-06 |
| AC021205.3 | -2.052426065 | 3.35E-10 | HNF4A-AS1 | -12.91786531 | 3.13E-10 |
| AL121845.4 | -2.053211936 | 2.06E-06 | RP11-42O15.3 | -12.95573935 | 1.17E-10 |
| AL133467.1 | -2.067114507 | 7.54E-04 | AC099668.5 | -12.96043381 | 7.62E-10 |
| AL031316.1 | -2.069408593 | 3.33E-05 | ADORA2A-AS1 | -13.58897027 | 3.77E-12 |
| AC005702.1 | -2.076705788 | 1.06E-12 | RP11-1151B14.3 | -13.63333325 | 5.00E-11 |
| LINC02198 | -2.097733514 | 2.38E-07 | HORMAD2-AS1 | -13.71834069 | 1.74E-11 |
| AC005920.4 | -2.101465566 | 2.48E-07 | RP11-119D9.1 | -13.72479724 | 6.29E-09 |
| AC025271.4 | -2.104305107 | 5.40E-08 | AC132217.4 | -13.92972246 | 3.38E-05 |
| AC007319.1 | -2.112000632 | 2.60E-05 | DBH-AS1 | -14.00928009 | 5.29E-11 |
| KANSL1-AS1 | -2.112628946 | 2.61E-09 | FAM99A | -14.7405029 | 2.63E-13 |
| AL078590.3 | -2.114666627 | 7.90E-08 | LINC01146 | -14.85611262 | 1.78E-11 |
| AC105942.1 | -2.131310735 | 2.56E-12 | RP4-763G1.2 | -15.12417834 | 2.43E-13 |
| AC112496.1 | -2.13595453 | 4.03E-08 | AP000355.2 | -15.26758254 | 3.90E-13 |
| AL021068.1 | -2.136772896 | 6.02E-06 | RP11-116D2.1 | -15.38954336 | 4.48E-12 |
| LINC01537 | -2.142172422 | 4.19E-06 | H19 | -15.42593789 | 4.89E-10 |
| AF111167.2 | -2.148707669 | 7.94E-11 | RP4-710M16.2 | -15.82807216 | 1.78E-13 |
| AC254633.1 | -2.149019891 | 7.74E-10 | RP11-122K13.7 | -15.97559785 | 1.16E-08 |
| AL109936.2 | -2.15033733 | 1.89E-09 | ITIH4-AS1 | -16.94634542 | 1.81E-15 |
| MGC27382 | -2.15035026 | 6.53E-06 | RP11-372E1.4 | -17.20379758 | 1.04E-15 |
| AL161669.1 | -2.151279725 | 1.09E-04 | RP11-328K4.1 | -17.80632932 | 2.74E-16 |
| B4GALT1-AS1 | -2.162926353 | 1.34E-10 | LINC01018 | -18.04310796 | 2.57E-14 |
| AL354707.1 | -2.174613784 | 5.14E-06 | CTC-505O3.2 | -18.18766757 | 1.37E-14 |
| AC024361.2 | -2.175261637 | 4.32E-10 | LINC01485 | -18.47199383 | 1.90E-12 |
| AL359715.2 | -2.179492736 | 1.28E-10 | HULC | -19.22391259 | 6.39E-10 |
| WARS2-IT1 | -2.209579272 | 2.35E-06 | CTD-3098H1.2 | -19.69799712 | 7.34E-09 |
| LINC00526 | -2.210431553 | 5.49E-23 | LINC00844 | -19.94897572 | 5.04E-16 |
| AL359232.1 | -2.21256224 | 3.84E-08 | RP11-622A1.2 | -20.11926536 | 9.06E-13 |
| LINC00870 | -2.224241449 | 2.74E-06 | LINC01554 | -20.52184268 | 2.31E-14 |
| AC037198.3 | -2.226111097 | 1.12E-05 |  |  |  |
| AL136040.1 | -2.23804829 | 6.18E-10 |  |  |  |
| AC009560.1 | -2.256109023 | 2.27E-10 |  |  |  |
| AC231981.1 | -2.256870632 | 4.39E-16 |  |  |  |
| AC114947.2 | -2.259821714 | 6.29E-11 |  |  |  |
| LINC00885 | -2.274980367 | 1.26E-04 |  |  |  |
| AC009159.3 | -2.281060896 | 3.93E-15 |  |  |  |
| AC022784.8 | -2.28312571 | 3.62E-06 |  |  |  |
| GK-AS1 | -2.28708041 | 1.68E-09 |  |  |  |
| AC129507.1 | -2.290302138 | 1.52E-07 |  |  |  |
| AC007953.1 | -2.292523322 | 3.50E-04 |  |  |  |
| AC007207.2 | -2.302578033 | 7.37E-04 |  |  |  |
| AL354811.1 | -2.304233227 | 3.41E-05 |  |  |  |
| LINC01474 | -2.332036589 | 3.93E-07 |  |  |  |
| AL121820.2 | -2.340967584 | 1.03E-03 |  |  |  |
| AC010735.2 | -2.344328425 | 2.51E-05 |  |  |  |
| MAFTRR | -2.344972127 | 2.26E-07 |  |  |  |
| AP003721.4 | -2.348238172 | 3.03E-13 |  |  |  |
| AL365259.1 | -2.351221704 | 2.62E-03 |  |  |  |
| AC068338.2 | -2.355835599 | 2.18E-18 |  |  |  |
| ELOVL2-AS1 | -2.356839359 | 2.07E-04 |  |  |  |
| AC025857.2 | -2.364307699 | 4.63E-10 |  |  |  |
| AL021328.1 | -2.367431357 | 7.17E-04 |  |  |  |
| LINC01128 | -2.367531434 | 6.24E-17 |  |  |  |
| UNQ6494 | -2.369399227 | 4.62E-07 |  |  |  |
| OGFR-AS1 | -2.370091663 | 1.53E-29 |  |  |  |
| AC027682.6 | -2.373694872 | 4.65E-06 |  |  |  |
| AL157786.1 | -2.374086367 | 1.32E-05 |  |  |  |
| LINC00365 | -2.396411245 | 6.67E-06 |  |  |  |
| AC026369.3 | -2.399463559 | 2.88E-05 |  |  |  |
| LINC00574 | -2.408447772 | 1.16E-06 |  |  |  |
| AC083900.1 | -2.415674846 | 8.95E-11 |  |  |  |
| AF127577.4 | -2.419912469 | 5.70E-16 |  |  |  |
| DKFZp779M0652 | -2.431631739 | 6.14E-08 |  |  |  |
| LINC02482 | -2.432794337 | 2.69E-09 |  |  |  |
| AC087482.1 | -2.441841072 | 3.82E-04 |  |  |  |
| AL163636.1 | -2.45490891 | 7.43E-12 |  |  |  |
| AC055720.2 | -2.455047396 | 4.32E-15 |  |  |  |
| AC092809.2 | -2.467962942 | 7.86E-04 |  |  |  |
| LINC01268 | -2.476681524 | 6.41E-07 |  |  |  |
| AL772337.3 | -2.47684766 | 2.44E-05 |  |  |  |
| DHRS4-AS1 | -2.480248278 | 5.82E-16 |  |  |  |
| LINC00987 | -2.486213596 | 2.13E-04 |  |  |  |
| CTD-3080P12.3 | -2.486702556 | 3.07E-03 |  |  |  |
| AC104958.2 | -2.488462224 | 6.66E-07 |  |  |  |
| AC104971.2 | -2.496869404 | 3.92E-07 |  |  |  |
| AP000919.4 | -2.497480946 | 6.81E-06 |  |  |  |
| AC010203.2 | -2.501948613 | 5.17E-08 |  |  |  |
| AL391832.2 | -2.529862771 | 6.37E-06 |  |  |  |
| AL445493.3 | -2.52989032 | 6.11E-08 |  |  |  |
| AC004825.2 | -2.530343191 | 4.66E-13 |  |  |  |
| AC144652.1 | -2.539284865 | 2.09E-12 |  |  |  |
| EP300-AS1 | -2.546930114 | 7.94E-07 |  |  |  |
| AC116036.2 | -2.547066215 | 4.78E-10 |  |  |  |
| AL157373.2 | -2.547336692 | 2.31E-09 |  |  |  |
| AP002026.1 | -2.551020563 | 5.31E-06 |  |  |  |
| AC053503.2 | -2.556050346 | 4.52E-08 |  |  |  |
| AC024145.1 | -2.556071302 | 1.71E-05 |  |  |  |
| AC110995.1 | -2.55894002 | 3.14E-06 |  |  |  |
| THRB-AS1 | -2.579383914 | 1.03E-11 |  |  |  |
| LINC00242 | -2.580089882 | 1.27E-15 |  |  |  |
| LINC01612 | -2.601526755 | 1.74E-03 |  |  |  |
| AC005674.1 | -2.601965718 | 2.57E-09 |  |  |  |
| AL137798.1 | -2.606062642 | 3.52E-03 |  |  |  |
| LINC02289 | -2.611465169 | 8.70E-13 |  |  |  |
| LINC01354 | -2.62995273 | 4.05E-07 |  |  |  |
| LINC02139 | -2.634692353 | 2.09E-09 |  |  |  |
| AC007364.1 | -2.643340509 | 6.45E-10 |  |  |  |
| AC021491.4 | -2.756831725 | 1.13E-11 |  |  |  |
| MGC32805 | -2.75836131 | 9.20E-04 |  |  |  |
| TMC3-AS1 | -2.765864485 | 1.60E-15 |  |  |  |
| AF165147.1 | -2.787373626 | 1.03E-09 |  |  |  |
| AL445686.2 | -2.789018542 | 2.70E-08 |  |  |  |
| AC009974.1 | -2.798960726 | 1.06E-10 |  |  |  |
| AL359715.4 | -2.800484387 | 4.89E-17 |  |  |  |
| HM13-AS1 | -2.804062979 | 1.30E-13 |  |  |  |
| AC090164.3 | -2.811567912 | 1.04E-11 |  |  |  |
| AL031722.1 | -2.822731191 | 4.73E-15 |  |  |  |
| TAT-AS1 | -2.838712523 | 4.14E-05 |  |  |  |
| AC109454.3 | -2.840339836 | 2.70E-03 |  |  |  |
| AP000473.1 | -2.862245984 | 1.39E-15 |  |  |  |
| AC244021.1 | -2.87177297 | 3.48E-08 |  |  |  |
| NADK2-AS1 | -2.87706062 | 2.93E-11 |  |  |  |
| LINC02453 | -2.890401245 | 6.85E-21 |  |  |  |
| AP001065.1 | -2.898178479 | 2.52E-05 |  |  |  |
| AGAP1-IT1 | -2.954358142 | 9.18E-14 |  |  |  |
| AP000253.1 | -2.964832526 | 5.99E-22 |  |  |  |
| LAMA5-AS1 | -2.986733157 | 9.88E-12 |  |  |  |
| AC012409.1 | -2.989522073 | 4.75E-11 |  |  |  |
| LINC02015 | -3.080445037 | 9.05E-07 |  |  |  |
| LINC00535 | -3.107667279 | 6.06E-19 |  |  |  |
| LINC01482 | -3.113801038 | 1.16E-09 |  |  |  |
| AC015689.1 | -3.149342433 | 1.43E-10 |  |  |  |
| AC099684.2 | -3.152809076 | 5.29E-10 |  |  |  |
| OSTN-AS1 | -3.17309999 | 2.86E-04 |  |  |  |
| FAM222A-AS1 | -3.187389645 | 2.95E-11 |  |  |  |
| POU6F2-AS1 | -3.206006206 | 1.16E-06 |  |  |  |
| AC005280.1 | -3.213688431 | 7.46E-16 |  |  |  |
| AC008760.2 | -3.216344161 | 2.95E-09 |  |  |  |
| AC103740.1 | -3.220434826 | 4.61E-11 |  |  |  |
| AC010336.2 | -3.236555104 | 1.43E-13 |  |  |  |
| AC097639.1 | -3.244381373 | 7.51E-22 |  |  |  |
| AC012313.9 | -3.24605849 | 3.01E-27 |  |  |  |
| AC004832.5 | -3.254067141 | 3.59E-28 |  |  |  |
| AC016395.1 | -3.301379651 | 9.79E-07 |  |  |  |
| AL023581.2 | -3.32975757 | 2.75E-26 |  |  |  |
| LINC02028 | -3.365646141 | 9.60E-13 |  |  |  |
| AC005550.3 | -3.365685945 | 1.18E-06 |  |  |  |
| AC007495.1 | -3.371300324 | 4.65E-06 |  |  |  |
| AL360181.1 | -3.40095093 | 1.22E-10 |  |  |  |
| SPATA41 | -3.452901395 | 4.66E-23 |  |  |  |
| AL160408.3 | -3.474589108 | 8.05E-07 |  |  |  |
| TMEM220-AS1 | -3.494640959 | 4.49E-15 |  |  |  |
| AC026469.1 | -3.50392985 | 1.76E-19 |  |  |  |
| AC105105.2 | -3.504156291 | 2.58E-06 |  |  |  |
| ADORA2A-AS1 | -3.51022343 | 2.42E-06 |  |  |  |
| LINC01687 | -3.522364613 | 1.14E-03 |  |  |  |
| AC105202.1 | -3.537778373 | 8.93E-19 |  |  |  |
| AC015908.3 | -3.567868592 | 1.61E-15 |  |  |  |
| AC105105.3 | -3.633187044 | 7.42E-13 |  |  |  |
| AL139385.1 | -3.634725029 | 4.87E-20 |  |  |  |
| AC005180.2 | -3.642080394 | 1.45E-16 |  |  |  |
| ALDH1L1-AS2 | -3.669228059 | 3.63E-10 |  |  |  |
| AC090150.1 | -3.675913993 | 6.15E-08 |  |  |  |
| AC007666.1 | -3.761731044 | 1.08E-20 |  |  |  |
| AC137056.1 | -3.807525022 | 5.28E-10 |  |  |  |
| AC099684.1 | -3.846620786 | 3.89E-15 |  |  |  |
| AC138356.1 | -3.867139319 | 1.23E-19 |  |  |  |
| AC004862.1 | -3.900678404 | 7.49E-07 |  |  |  |
| AL161630.1 | -3.935633492 | 4.40E-07 |  |  |  |
| BX547991.1 | -3.936611429 | 1.17E-13 |  |  |  |
| AL391095.2 | -3.939537866 | 1.97E-06 |  |  |  |
| AL080248.1 | -3.94681833 | 1.72E-07 |  |  |  |
| AL592494.1 | -3.961589862 | 1.37E-16 |  |  |  |
| F11-AS1 | -3.966225547 | 2.41E-16 |  |  |  |
| AP001781.2 | -3.977483093 | 3.28E-13 |  |  |  |
| AC112206.2 | -3.991267727 | 3.45E-05 |  |  |  |
| AC083841.1 | -3.996170542 | 4.92E-11 |  |  |  |
| AL161645.1 | -4.051359163 | 2.05E-15 |  |  |  |
| AC099509.1 | -4.073582652 | 4.57E-16 |  |  |  |
| LINC02418 | -4.075599941 | 3.69E-08 |  |  |  |
| LINC01146 | -4.159884987 | 9.18E-17 |  |  |  |
| HULC | -4.197684921 | 4.26E-07 |  |  |  |
| AL355096.1 | -4.235781933 | 3.62E-09 |  |  |  |
| AL355974.2 | -4.285141938 | 1.81E-18 |  |  |  |
| AL354872.2 | -4.288980715 | 9.50E-26 |  |  |  |
| TPRG1-AS1 | -4.327413458 | 1.11E-23 |  |  |  |
| AC020978.5 | -4.332149666 | 1.09E-23 |  |  |  |
| AP000355.1 | -4.366265748 | 2.79E-06 |  |  |  |
| AP000851.2 | -4.413393539 | 4.03E-08 |  |  |  |
| AC244100.3 | -4.417149141 | 4.97E-22 |  |  |  |
| AC115619.1 | -4.437299685 | 8.17E-10 |  |  |  |
| AL356056.3 | -4.48102279 | 4.13E-26 |  |  |  |
| AC022816.1 | -4.535315356 | 3.82E-10 |  |  |  |
| LINC02275 | -4.544875238 | 2.62E-09 |  |  |  |
| LINC00844 | -4.555333172 | 4.09E-06 |  |  |  |
| AC068631.1 | -4.557326869 | 7.56E-28 |  |  |  |
| AC018467.1 | -4.582174007 | 2.54E-10 |  |  |  |
| FP700125.1 | -4.620841546 | 9.69E-12 |  |  |  |
| AC243836.1 | -4.694489872 | 1.90E-21 |  |  |  |
| LINC01727 | -4.724296558 | 5.00E-11 |  |  |  |
| LINC01485 | -4.953703075 | 2.16E-13 |  |  |  |
| AC004160.1 | -4.972106618 | 2.59E-24 |  |  |  |
| LINC01780 | -4.988040138 | 1.48E-35 |  |  |  |
| AC007298.2 | -4.993241151 | 2.55E-13 |  |  |  |
| LINC01767 | -5.010923967 | 5.30E-23 |  |  |  |
| AL161740.1 | -5.030414488 | 1.30E-21 |  |  |  |
| HS1BP3-IT1 | -5.082535089 | 2.31E-18 |  |  |  |
| AL161668.3 | -5.096606793 | 6.82E-24 |  |  |  |
| AC092071.1 | -5.144924894 | 2.76E-09 |  |  |  |
| AP006285.1 | -5.199134263 | 4.26E-07 |  |  |  |
| LINC01702 | -5.258656345 | 2.19E-10 |  |  |  |
| LINC00261 | -5.259942704 | 2.72E-10 |  |  |  |
| LINC01370 | -5.284546736 | 3.06E-06 |  |  |  |
| AC119424.1 | -5.301070182 | 7.98E-16 |  |  |  |
| AJ009632.2 | -5.306571938 | 2.50E-12 |  |  |  |
| AC079360.1 | -5.312173455 | 2.81E-21 |  |  |  |
| LINC02027 | -5.357369414 | 2.04E-09 |  |  |  |
| AL359915.1 | -5.358738776 | 1.43E-21 |  |  |  |
| AC137723.1 | -5.386612888 | 3.36E-32 |  |  |  |
| AP003716.1 | -5.473580024 | 1.13E-26 |  |  |  |
| LINC01595 | -5.479178056 | 3.14E-05 |  |  |  |
| LINC01625 | -5.518975374 | 4.67E-20 |  |  |  |
| LINC02362 | -5.527481836 | 9.98E-30 |  |  |  |
| HORMAD2-AS1 | -5.535420208 | 2.50E-25 |  |  |  |
| FAM99B | -5.614102498 | 1.20E-07 |  |  |  |
| AC007423.1 | -5.699991524 | 2.62E-12 |  |  |  |
| AP001043.1 | -5.725174627 | 3.92E-27 |  |  |  |
| LINC01639 | -5.742348066 | 9.69E-13 |  |  |  |
| AP001783.1 | -5.750487269 | 2.65E-18 |  |  |  |
| AP006216.2 | -5.75512186 | 1.24E-13 |  |  |  |
| AC010280.2 | -5.755364736 | 4.57E-36 |  |  |  |
| AC099508.2 | -5.782870568 | 1.38E-21 |  |  |  |
| AC021074.3 | -5.784388641 | 3.73E-09 |  |  |  |
| FAM99A | -5.989921911 | 3.72E-07 |  |  |  |
| CR936218.2 | -6.018498232 | 8.87E-23 |  |  |  |
| HNF4A-AS1 | -6.027438927 | 5.28E-17 |  |  |  |
| AL592182.2 | -6.041021061 | 1.04E-25 |  |  |  |
| LINC01831 | -6.197034652 | 4.75E-15 |  |  |  |
| LINC01554 | -6.212016577 | 1.45E-16 |  |  |  |
| AL121827.1 | -6.923176671 | 6.13E-11 |  |  |  |
| AC104809.1 | -7.203296606 | 1.81E-16 |  |  |  |
| AC079598.1 | -7.605385148 | 2.00E-23 |  |  |  |
| CPS1-IT1 | -7.953567249 | 9.35E-34 |  |  |  |
| ITIH4-AS1 | -13.55844 | 4.12E-06 |  |  |  |
| H19 | -16.55881 | 8.04E-11 |  |  |  |
