# Supplementary material for: Long non-coding RNA LINC00665 promotes gemcitabine resistance of Cholangiocarcinoma cells via regulating EMT and stemness properties through miR-424-5p/BCL9L axis
Source: Cell Death Dis. 2021 Jan 12;12(1):72. doi: 10.1038/s41419-020-03346-4 (PMC7803957; doi:10.1038/s41419-020-03346-4)
Supplement: Supplementary file 12 — Supplementary Table 4 [file 41419_2020_3346_MOESM12_ESM.docx]

**Supplementary Table 4. Dysregulated lncRNAs identified in HuCCT1-Gem and SNU-245-Gem cells both.**

|  | **HuCCT1-Gem** | | **SNU-245-Gem** | |
| --- | --- | --- | --- | --- |
| **Symbol** | **log2 Fold Change** | **p Value** | **log2 Fold Change** | **p Value** |
| LINC01057 | 10.28211252 | 1.58E-09 | 10.51173036 | 2.25E-09 |
| UCA1 | 9.612944835 | 5.26E-10 | 19.38596648 | 2.25E-07 |
| FAM83H-AS1 | 7.210580906 | 2.74E-08 | 10.30082987 | 3.91E-08 |
| PVT1 | 6.979804296 | 2.12E-15 | 11.51122444 | 1.88E-09 |
| LINC01559 | 6.89882315 | 4.85E-16 | 11.14589867 | 4.46E-06 |
| LINC01833 | 6.418589225 | 5.21E-10 | 7.131765806 | 5.21E-10 |
| LINC00665 | 5.93103002 | 1.44E-11 | 10.86192752 | 1.12E-05 |
| RP11-462L8.1 | 5.867049353 | 3.12E-06 | 8.381499076 | 4.46E-06 |
| LINC00707 | 5.5565671 | 8.01E-07 | 4.908030887 | 5.81E-09 |
| HOXB-AS3 | 5.469783891 | 3.15E-05 | 7.813976987 | 4.50E-05 |
| LINC00152 | 5.438840923 | 1.39E-10 | 8.798109445 | 4.32E-07 |
| RP11-304L19.12 | 5.39289273 | 1.42E-10 | 8.23753704 | 8.06E-05 |
| LINC02041 | 5.224451218 | 4.77E-10 | 5.982723576 | 4.77E-10 |
| LINC00941 | 5.171772012 | 8.50E-16 | 7.03454806 | 2.13E-04 |
| RP11-284F21.9 | 5.126360775 | 9.42E-08 | 7.231475074 | 1.63E-06 |
| AC105219.4 | 5.066932222 | 8.04E-11 | 5.629924691 | 8.04E-11 |
| HOXB-AS4 | 4.72688327 | 2.01E-03 | 6.853980742 | 1.91E-03 |
| AC147651.1 | 4.605134951 | 5.30E-10 | 5.116816612 | 5.30E-10 |
| MNX1-AS1 | 4.40930659 | 7.42E-18 | 7.727580843 | 1.06E-17 |
| SLCO4A1-AS1 | 4.389985292 | 4.92E-11 | 4.877761436 | 4.92E-11 |
| AC026368.1 | 4.352175271 | 1.87E-10 | 4.730654143 | 1.78E-10 |
| LINC00511 | 4.273534544 | 9.24E-24 | 11.25183086 | 4.16E-10 |
| RNF144A-AS1 | 4.268822199 | 3.69E-04 | 4.289792189 | 3.51E-04 |
| ELFN2 | 4.1158441 | 3.38E-11 | 4.296803762 | 8.69E-05 |
| AC025154.2 | 4.08796369 | 1.23E-09 | 6.096485131 | 1.29E-06 |
| AL596223.2 | 3.956399529 | 1.75E-07 | 5.736779317 | 1.66E-07 |
| AC007128.1 | 3.845170155 | 1.58E-03 | 4.575496725 | 1.50E-03 |
| HAGLR | 3.743433687 | 2.52E-07 | 5.317978846 | 2.39E-07 |
| AC106772.1 | 3.673823012 | 3.11E-03 | 3.282543367 | 2.95E-03 |
| AL109976.1 | 3.669425208 | 5.71E-11 | 4.07713912 | 5.71E-11 |
| HOXC-AS1 | 3.545177951 | 2.45E-04 | 5.575508029 | 2.33E-04 |
| AP003419.4 | 3.305755578 | 7.06E-11 | 3.673061753 | 7.06E-11 |
| MAFG-AS1 | 3.247134818 | 6.64E-27 | 5.607927575 | 6.64E-27 |
| LINC00992 | 3.186240822 | 7.75E-11 | 3.54026758 | 7.75E-11 |
| CRNDE | 3.07702532 | 1.19E-13 | 8.706040175 | 1.83E-06 |
| AC010247.2 | 3.063646512 | 2.25E-03 | 2.992287442 | 2.14E-03 |
| AC124798.1 | 2.956383928 | 1.04E-05 | 3.016756696 | 9.88E-06 |
| LINC01836 | 2.896229677 | 6.64E-11 | 3.218032974 | 6.64E-11 |
| AC023449.2 | 2.850982236 | 4.24E-04 | 2.683924242 | 4.03E-04 |
| LINC01123 | 2.83057322 | 1.05E-12 | 12.17976465 | 2.68E-10 |
| LINC00622 | 2.76412219 | 3.81E-07 | 3.71767475 | 3.62E-07 |
| AC016773.1 | 2.750682768 | 5.93E-10 | 3.056314187 | 5.93E-10 |
| AP003390.1 | 2.672621827 | 1.13E-04 | 2.585301649 | 1.07E-04 |
| SZT2-AS1 | 2.652719363 | 1.60E-03 | 7.347783224 | 7.88E-06 |
| AC023908.3 | 2.593893476 | 4.80E-10 | 2.882103862 | 4.80E-10 |
| AL117335.1 | 2.55719864 | 6.84E-11 | 2.841331822 | 6.84E-11 |
| AF131215.6 | 2.508122462 | 5.78E-11 | 2.786802735 | 5.78E-11 |
| LINC01106 | 2.487325045 | 1.73E-10 | 9.635747002 | 8.59E-10 |
| AC233280.1 | 2.472281286 | 9.98E-05 | 3.584807865 | 9.48E-05 |
| PAX8-AS1 | 2.452243491 | 3.69E-05 | 8.763288732 | 8.92E-05 |
| AC008764.8 | 2.448864709 | 8.41E-11 | 2.09304676 | 8.41E-11 |
| FAM66C | 2.408846179 | 3.63E-13 | 7.582145905 | 2.18E-07 |
| GAS5 | 2.406641292 | 1.25E-13 | 5.684330671 | 1.19E-13 |
| LINC01133 | 2.354479208 | 3.77E-03 | 3.413994852 | 3.58E-03 |
| VIM-AS1 | 2.335757059 | 6.30E-10 | 2.595285621 | 6.30E-10 |
| AL365203.3 | 2.315731924 | 4.50E-10 | 2.573035471 | 4.50E-10 |
| AL021154.1 | 2.248348879 | 7.54E-04 | 3.060105875 | 7.16E-04 |
| PRR7-AS1 | 2.194724241 | 5.42E-07 | 6.90762319 | 2.66E-06 |
| Z69733.1 | 2.169491911 | 3.96E-05 | 3.580763271 | 3.76E-05 |
| AGAP2-AS1 | 2.097659447 | 2.27E-10 | 7.530289535 | 8.47E-06 |
| AL731569.1 | 2.089432553 | 3.93E-10 | 2.321591725 | 3.93E-10 |
| AL022322.1 | 2.079215345 | 8.02E-11 | 2.572287126 | 8.02E-11 |
| AL391056.1 | 2.064061679 | 2.17E-03 | 4.992889435 | 2.06E-03 |
| FAM201A | 2.020146151 | 4.06E-08 | 9.443322247 | 2.38E-09 |
| AC007319.1 | -2.112000632 | 2.60E-05 | -7.880220032 | 1.26E-04 |
| AF111167.2 | -2.148707669 | 7.94E-11 | -2.387452966 | 7.94E-11 |
| AC024361.2 | -2.175261637 | 4.32E-10 | -2.416957374 | 4.32E-10 |
| AL136040.1 | -2.23804829 | 6.18E-10 | -4.486720322 | 6.18E-10 |
| AC114947.2 | -2.259821714 | 6.29E-11 | -2.510913016 | 6.29E-11 |
| AC025857.2 | -2.364307699 | 4.63E-10 | -2.020775811 | 4.63E-10 |
| AC083900.1 | -2.415674846 | 8.95E-11 | -2.684083162 | 8.95E-11 |
| CTD-3080P12.3 | -2.486702556 | 3.07E-03 | -7.606137454 | 3.52E-07 |
| AC116036.2 | -2.547066215 | 4.78E-10 | -2.830073572 | 4.78E-10 |
| AL137798.1 | -2.606062642 | 3.52E-03 | -3.778790831 | 3.34E-03 |
| LINC02289 | -2.611465169 | 8.70E-13 | -5.786624495 | 8.27E-13 |
| MGC32805 | -2.75836131 | 9.20E-04 | -9.452709575 | 3.16E-07 |
| NADK2-AS1 | -2.87706062 | 2.93E-11 | -8.594249503 | 1.18E-06 |
| LAMA5-AS1 | -2.986733157 | 9.88E-12 | -10.5149019 | 2.23E-05 |
| AC099684.2 | -3.152809076 | 5.29E-10 | -3.503121195 | 5.29E-10 |
| AC012313.9 | -3.24605849 | 3.01E-27 | -3.606731655 | 3.01E-27 |
| AL023581.2 | -3.32975757 | 2.75E-26 | -2.03967676 | 2.75E-26 |
| TMEM220-AS1 | -3.494640959 | 4.49E-15 | -10.50646134 | 4.86E-08 |
| ADORA2A-AS1 | -3.51022343 | 2.42E-06 | -13.58897027 | 3.77E-12 |
| AC137056.1 | -3.807525022 | 5.28E-10 | -4.230583358 | 5.28E-10 |
| AC083841.1 | -3.996170542 | 4.92E-11 | -4.440189491 | 4.92E-11 |
| LINC01146 | -4.159884987 | 9.18E-17 | -14.85611262 | 1.78E-11 |
| HULC | -4.197684921 | 4.26E-07 | -19.22391259 | 6.39E-10 |
| AL354872.2 | -4.288980715 | 9.50E-26 | -5.321089683 | 9.50E-26 |
| TPRG1-AS1 | -4.327413458 | 1.11E-23 | -8.933465275 | 3.17E-06 |
| AL356056.3 | -4.48102279 | 4.13E-26 | -4.978914211 | 4.13E-26 |
| LINC00844 | -4.555333172 | 4.09E-06 | -19.94897572 | 5.04E-16 |
| AC068631.1 | -4.557326869 | 7.56E-28 | -4.619252077 | 7.56E-28 |
| LINC01727 | -4.724296558 | 5.00E-11 | -8.249218398 | 5.00E-11 |
| LINC01485 | -4.953703075 | 2.16E-13 | -18.47199383 | 1.90E-12 |
| HS1BP3-IT1 | -5.082535089 | 2.31E-18 | -11.61633345 | 3.70E-08 |
| LINC00261 | -5.259942704 | 2.72E-10 | -11.76301315 | 9.20E-07 |
| LINC01370 | -5.284546736 | 3.06E-06 | -12.63596716 | 1.02E-10 |
| AP003716.1 | -5.473580024 | 1.13E-26 | -6.081755582 | 1.13E-26 |
| HORMAD2-AS1 | -5.535420208 | 2.50E-25 | -13.71834069 | 1.74E-11 |
| FAM99B | -5.614102498 | 1.20E-07 | -12.40106836 | 4.48E-14 |
| AP001043.1 | -5.725174627 | 3.92E-27 | -5.25019403 | 3.92E-27 |
| FAM99A | -5.989921911 | 3.72E-07 | -14.7405029 | 2.63E-13 |
| HNF4A-AS1 | -6.027438927 | 5.28E-17 | -12.91786531 | 3.13E-10 |
| LINC01554 | -6.212016577 | 1.45E-16 | -20.52184268 | 2.31E-14 |
| AL121827.1 | -6.923176671 | 6.13E-11 | -6.581307412 | 6.13E-11 |
| ITIH4-AS1 | -13.55844 | 4.12E-06 | -16.94634542 | 1.81E-15 |
| H19 | -16.55881 | 8.04E-11 | -15.42593789 | 4.89E-10 |
