# Supplementary material for: Long non-coding RNA LINC00665 promotes gemcitabine resistance of Cholangiocarcinoma cells via regulating EMT and stemness properties through miR-424-5p/BCL9L axis
Source: Cell Death Dis. 2021 Jan 12;12(1):72. doi: 10.1038/s41419-020-03346-4 (PMC7803957; doi:10.1038/s41419-020-03346-4)
Supplement: Supplementary file 13 — Supplementary Table 5 [file 41419_2020_3346_MOESM13_ESM.docx]

**Supplementary Table 5. Conservative microRNA targeting sites for BCL9L predicted by TargetScanHuman 7.2.**

| **miRNA family** | **Conserved sites** | | | | **Poorly conserved sites** | | | | **6mer sites** |
| --- | --- | --- | --- | --- | --- | --- | --- | --- | --- |
|  | **Total** | **8mer** | **7mer-m8** | **7mer-A1** | **Total** | **8mer** | **7mer-m8** | **7mer-A1** |  |
| miR-138-5p | 3 | 1 | 1 | 1 | 1 | 0 | 1 | 0 | 0 |
| miR-22-3p | 2 | 0 | 2 | 0 | 1 | 1 | 0 | 0 | 1 |
| miR-124-3p.1 | 1 | 0 | 1 | 0 | 1 | 0 | 1 | 0 | 1 |
| miR-34-5p/449-5p | 1 | 0 | 1 | 0 | 1 | 0 | 1 | 0 | 3 |
| miR-10-5p | 0 | 0 | 0 | 0 | 1 | 0 | 1 | 0 | 2 |
| miR-184 | 0 | 0 | 0 | 0 | 1 | 0 | 1 | 0 | 1 |
| miR-135-5p | 1 | 1 | 0 | 0 | 0 | 0 | 0 | 0 | 3 |
| miR-101-3p.1 | 1 | 1 | 0 | 0 | 0 | 0 | 0 | 0 | 0 |
| miR-23-3p | 1 | 0 | 0 | 1 | 0 | 0 | 0 | 0 | 0 |
| miR-15-5p/16-5p/195-5p/424-5p/497-5p | 2 | 1 | 1 | 0 | 0 | 0 | 0 | 0 | 4 |
| miR-31-5p | 1 | 0 | 1 | 0 | 0 | 0 | 0 | 0 | 1 |
| miR-124-3p.2/506-3p | 0 | 0 | 0 | 0 | 1 | 0 | 0 | 1 | 0 |
| miR-551-3p | 0 | 0 | 0 | 0 | 1 | 0 | 1 | 0 | 0 |
| miR-29-3p | 1 | 0 | 1 | 0 | 1 | 0 | 1 | 0 | 0 |
| miR-126-3p.2 | 0 | 0 | 0 | 0 | 1 | 0 | 0 | 1 | 0 |
| miR-204-5p/211-5p | 1 | 1 | 0 | 0 | 1 | 0 | 1 | 0 | 2 |
| miR-26-5p | 0 | 0 | 0 | 0 | 1 | 0 | 0 | 1 | 0 |
| miR-125-5p | 0 | 0 | 0 | 0 | 2 | 0 | 1 | 1 | 0 |
| miR-150-5p | 1 | 1 | 0 | 0 | 0 | 0 | 0 | 0 | 3 |
| miR-140-3p.1 | 0 | 0 | 0 | 0 | 2 | 0 | 1 | 1 | 0 |
| miR-205-5p | 0 | 0 | 0 | 0 | 1 | 1 | 0 | 0 | 0 |
| miR-130-3p/301-3p/454-3p | 0 | 0 | 0 | 0 | 2 | 0 | 1 | 1 | 0 |
| miR-137 | 0 | 0 | 0 | 0 | 1 | 1 | 0 | 0 | 0 |
| miR-128-3p | 0 | 0 | 0 | 0 | 1 | 0 | 1 | 0 | 0 |
| miR-146-5p | 0 | 0 | 0 | 0 | 1 | 0 | 1 | 0 | 2 |
| miR-133a-3p.2/133b | 0 | 0 | 0 | 0 | 1 | 0 | 1 | 0 | 0 |
| miR-455-3p.2 | 0 | 0 | 0 | 0 | 1 | 0 | 1 | 0 | 0 |
| miR-101-3p.2 | 1 | 0 | 1 | 0 | 0 | 0 | 0 | 0 | 0 |
| miR-212-5p | 0 | 0 | 0 | 0 | 2 | 0 | 0 | 2 | 2 |
| miR-199-5p | 0 | 0 | 0 | 0 | 1 | 0 | 1 | 0 | 3 |
| miR-208-3p | 0 | 0 | 0 | 0 | 1 | 0 | 1 | 0 | 0 |
| miR-148-3p/152-3p | 0 | 0 | 0 | 0 | 1 | 0 | 1 | 0 | 0 |
| miR-193a-5p | 0 | 0 | 0 | 0 | 1 | 0 | 1 | 0 | 1 |
| miR-140-3p.2 | 1 | 0 | 0 | 1 | 0 | 0 | 0 | 0 | 0 |
| miR-338-3p | 0 | 0 | 0 | 0 | 1 | 0 | 0 | 1 | 2 |
| miR-24-3p | 0 | 0 | 0 | 0 | 1 | 0 | 0 | 1 | 3 |
| miR-144-3p | 1 | 0 | 0 | 1 | 0 | 0 | 0 | 0 | 0 |
| miR-129-5p | 0 | 0 | 0 | 0 | 1 | 0 | 0 | 1 | 0 |
| miR-383-5p.1 | 0 | 0 | 0 | 0 | 1 | 0 | 0 | 1 | 0 |
| miR-217 | 1 | 0 | 0 | 1 | 0 | 0 | 0 | 0 | 1 |
| miR-455-3p.1 | 0 | 0 | 0 | 0 | 1 | 0 | 0 | 1 | 1 |
| miR-103-3p/107 | 1 | 0 | 0 | 1 | 0 | 0 | 0 | 0 | 5 |
| miR-133a-3p.1 | 0 | 0 | 0 | 0 | 1 | 0 | 0 | 1 | 0 |
| miR-296-5p | 0 | 0 | 0 | 0 | 1 | 0 | 1 | 0 | 3 |
| miR-423-5p | 0 | 0 | 0 | 0 | 2 | 0 | 1 | 1 | 4 |
| miR-3064-5p | 1 | 1 | 0 | 0 | 1 | 0 | 0 | 1 | 2 |
| miR-874-3p | 0 | 0 | 0 | 0 | 3 | 1 | 2 | 0 | 3 |
| miR-485-5p | 0 | 0 | 0 | 0 | 3 | 0 | 3 | 0 | 1 |
| miR-151-5p | 0 | 0 | 0 | 0 | 1 | 0 | 1 | 0 | 2 |
| miR-328-3p | 0 | 0 | 0 | 0 | 2 | 0 | 2 | 0 | 3 |
| miR-873-5p.2 | 1 | 0 | 1 | 0 | 3 | 0 | 3 | 0 | 4 |
| miR-487-3p | 0 | 0 | 0 | 0 | 1 | 0 | 0 | 1 | 0 |
| miR-503-5p | 1 | 0 | 0 | 1 | 0 | 0 | 0 | 0 | 5 |
| miR-339-5p | 0 | 0 | 0 | 0 | 2 | 0 | 1 | 1 | 0 |
| miR-760 | 0 | 0 | 0 | 0 | 3 | 1 | 1 | 1 | 0 |
| miR-127-3p | 0 | 0 | 0 | 0 | 1 | 0 | 1 | 0 | 0 |
| miR-154-5p | 0 | 0 | 0 | 0 | 1 | 0 | 1 | 0 | 2 |
| miR-491-5p | 0 | 0 | 0 | 0 | 1 | 0 | 1 | 0 | 5 |
| miR-362-5p/500b-5p | 0 | 0 | 0 | 0 | 1 | 0 | 0 | 1 | 0 |
| miR-28-5p/708-5p | 0 | 0 | 0 | 0 | 2 | 0 | 1 | 1 | 2 |
| miR-1306-5p | 1 | 1 | 0 | 0 | 3 | 0 | 3 | 0 | 1 |
| miR-665 | 0 | 0 | 0 | 0 | 1 | 0 | 0 | 1 | 2 |
| miR-331-3p | 0 | 0 | 0 | 0 | 2 | 0 | 2 | 0 | 1 |
| miR-504-5p.1 | 0 | 0 | 0 | 0 | 2 | 0 | 2 | 0 | 1 |
| miR-149-5p | 0 | 0 | 0 | 0 | 3 | 0 | 1 | 2 | 1 |
| miR-134-5p | 0 | 0 | 0 | 0 | 1 | 0 | 0 | 1 | 0 |
| miR-483-3p.1 | 0 | 0 | 0 | 0 | 1 | 0 | 0 | 1 | 1 |
| miR-483-3p.2 | 0 | 0 | 0 | 0 | 1 | 0 | 0 | 1 | 2 |
| miR-532-3p | 1 | 0 | 0 | 1 | 1 | 0 | 1 | 0 | 2 |
| miR-188-5p | 0 | 0 | 0 | 0 | 1 | 1 | 0 | 0 | 1 |
| miR-668-3p | 0 | 0 | 0 | 0 | 1 | 1 | 0 | 0 | 0 |
| miR-496.2 | 0 | 0 | 0 | 0 | 1 | 1 | 0 | 0 | 0 |
| miR-409-5p | 0 | 0 | 0 | 0 | 1 | 0 | 1 | 0 | 0 |
| miR-877-5p | 0 | 0 | 0 | 0 | 1 | 0 | 1 | 0 | 0 |
| miR-876-5p | 0 | 0 | 0 | 0 | 1 | 0 | 0 | 1 | 0 |
| miR-136-5p | 1 | 0 | 0 | 1 | 0 | 0 | 0 | 0 | 2 |
| miR-410-3p | 1 | 0 | 0 | 1 | 0 | 0 | 0 | 0 | 0 |
| miR-496.1 | 0 | 0 | 0 | 0 | 1 | 0 | 0 | 1 | 1 |
| miR-379-5p | 0 | 0 | 0 | 0 | 1 | 0 | 0 | 1 | 0 |
| miR-325-3p | 0 | 0 | 0 | 0 | 1 | 0 | 0 | 1 | 0 |
