# Supplementary material for: Long non-coding RNA LINC00665 promotes gemcitabine resistance of Cholangiocarcinoma cells via regulating EMT and stemness properties through miR-424-5p/BCL9L axis
Source: Cell Death Dis. 2021 Jan 12;12(1):72. doi: 10.1038/s41419-020-03346-4 (PMC7803957; doi:10.1038/s41419-020-03346-4)
Supplement: Supplementary file 14 — Supplementary Table 6 [file 41419_2020_3346_MOESM14_ESM.docx]

**Supplementary Table 6. MicroRNAs predicted to interact with LINC00665 and BCL9L both.**

| **#Transcript_ID** | **Gene_ID(Gene_Name)** | **Mirna_Name(miRBase_version)** | **Score** |
| --- | --- | --- | --- |
| >ENST00000590657 | ENSG00000232677(LINC00665) | hsa-miR-424-5p | 0.983 |
| 19:319-336 | 8mer | 0.069 |  |
| 19:1858-1877 | 6mer | 0.005 |  |
| 19:1794-1798 | 6mer | 0.004 |  |
| >ENST00000585356 | ENSG00000232677(LINC00665) | hsa-miR-424-5p | 0.98 |
| 19:319-336 | 8mer | 0.069 |  |
| 19:1071-1095 | 6mer | 0.006 |  |
| >ENST00000438368 | ENSG00000232677(LINC00665) | hsa-miR-129-5p | 0.977 |
| 19:74-92 | 6mer | 0.031 |  |
| 19:361-383 | 6mer | 0.02 |  |
| 19:64-90 | 6mer | 0.01 |  |
| 19:229-251 | 6mer | 0.006 |  |
| 19:1402-1411 | 6mer | 0.005 |  |
| >ENST00000590622 | ENSG00000232677(LINC00665) | hsa-miR-129-5p | 0.973 |
| 19:109-127 | 6mer | 0.03 |  |
| 19:396-418 | 6mer | 0.019 |  |
| 19:99-125 | 6mer | 0.01 |  |
| 19:264-286 | 6mer | 0.006 |  |
| 19:1278-1287 | 6mer | 0.005 |  |
| >ENST00000591372 | ENSG00000232677(LINC00665) | hsa-miR-3064-5p | 0.985 |
| 19:1814-1840 | 9mer | 0.042 |  |
| 19:2434-2455 | 8mer | 0.033 |  |
| 19:384-411 | 6mer | 0.005 |  |
| >ENST00000585356 | ENSG00000232677(LINC00665) | hsa-miR-485-5p | 0.953 |
| 19:573-592 | 8mer | 0.06 |  |
| >ENST00000590657 | ENSG00000232677(LINC00665) | hsa-miR-485-5p | 0.988 |
| 19:573-592 | 8mer | 0.057 |  |
| 19:1318-1336 | 7mer | 0.016 |  |
| >ENST00000585356 | ENSG00000232677(LINC00665) | hsa-miR-28-5p | 0.987 |
| 19:327-351 | 7mer | 0.029 |  |
| 19:89-109 | 7mer | 0.028 |  |
| 19:105-126 | 6mer | 0.024 |  |
| >ENST00000449434 | ENSG00000232677(LINC00665) | hsa-miR-28-5p | 0.928 |
| 19:79-99 | 7mer | 0.029 |  |
| 19:95-116 | 6mer | 0.024 |  |
| >ENST00000590657 | ENSG00000232677(LINC00665) | hsa-miR-28-5p | 0.995 |
| 19:327-351 | 7mer | 0.029 |  |
| 19:89-109 | 7mer | 0.028 |  |
| 19:105-126 | 6mer | 0.024 |  |
| 19:1548-1570 | 6mer | 0.009 |  |
| 19:1862-1882 | 7mer | 0.007 |  |
| >ENST00000585356 | ENSG00000232677(LINC00665) | hsa-miR-708-5p | 0.988 |
| 19:89-111 | 7mer | 0.031 |  |
| 19:327-343 | 7mer | 0.029 |  |
| 19:105-131 | 6mer | 0.023 |  |
| >ENST00000449434 | ENSG00000232677(LINC00665) | hsa-miR-708-5p | 0.938 |
| 19:79-101 | 7mer | 0.032 |  |
| 19:95-121 | 6mer | 0.023 |  |
| >ENST00000590657 | ENSG00000232677(LINC00665) | hsa-miR-708-5p | 0.996 |
| 19:89-111 | 7mer | 0.031 |  |
| 19:327-343 | 7mer | 0.029 |  |
| 19:105-131 | 6mer | 0.023 |  |
| 19:1548-1571 | 6mer | 0.009 |  |
| 19:1862-1882 | 7mer | 0.007 |  |
| >ENST00000412740 | ENSG00000232677(LINC00665) | hsa-miR-665 | 0.994 |
| 19:267-283 | 8mer | 0.073 |  |
| 19:80-95 | 8mer+wobble | 0.02 |  |
| >ENST00000427868 | ENSG00000232677(LINC00665) | hsa-miR-665 | 0.986 |
| 19:479-495 | 8mer | 0.08 |  |
| >ENST00000449434 | ENSG00000232677(LINC00665) | hsa-miR-665 | 0.98 |
| 19:344-360 | 8mer | 0.074 |  |
| >ENST00000585356 | ENSG00000232677(LINC00665) | hsa-miR-665 | 0.948 |
| 19:944-960 | 8mer | 0.054 |  |
| 19:1459-1483 | 6mer | 0.005 |  |
| >ENST00000438368 | ENSG00000232677(LINC00665) | hsa-miR-665 | 0.928 |
| 19:557-583 | 7mer | 0.032 |  |
| 19:1293-1305 | 7mer | 0.014 |  |
| 19:1764-1788 | 6mer | 0.005 |  |
| 19:269-293 | 6mer | 0.001 |  |
| >ENST00000590657 | ENSG00000232677(LINC00665) | hsa-miR-136-5p | 0.904 |
| 19:670-693 | 7mer | 0.018 |  |
| 19:342-368 | 6mer | 0.016 |  |
| 19:2236-2260 | 6mer | 0.013 |  |
| >ENST00000590657 | ENSG00000232677(LINC00665) | hsa-miR-410-3p | 0.985 |
| 19:276-296 | 9mer | 0.06 |  |
| 19:1126-1141 | 6mer | 0.012 |  |
| 19:981-1001 | 6mer | 0.007 |  |
| >ENST00000585356 | ENSG00000232677(LINC00665) | hsa-miR-410-3p | 0.955 |
| 19:276-296 | 9mer | 0.061 |  |
